# Whole-genome sequencing revealed genetic diversity, structure and patterns of selection in Guizhou indigenous chickens

**DOI:** 10.1186/s12864-023-09621-w

**Published:** 2023-09-26

**Authors:** Dan Xu, Wei Zhu, Youhao Wu, Shuo Wei, Gang Shu, Yaofu Tian, Xiaohui Du, Jigao Tang, Yulong Feng, Gemin Wu, Xue Han, Xiaoling Zhao

**Affiliations:** 1 Farm Animal Genetic Resources Exploration and Innovation Key Laboratory of Sichuan Province, Sichuan Agricultural University, Ya’an, P. R. China; 2 Key Laboratory of Livestock and Poultry Multi-Omics, MinistryofAgricultureandRuralAffairs, College of Animal Science and Technology(Institute of Animal Genetics and Breeding), Sichuan Agricultural University, Ya’an, P. R. China; 3 Department of Basic Veterinary Medicine, Sichuan Agricultural University, Chengdu, Sichuan China; 4 Institute of Animal Husbandry and Veterinary Medicine, Guizhou Academy of Agricultural Sciences, Guiyang, Guizhou Province China

**Keywords:** Guizhou indigenous chickens, Genetic diversity, Population structure, Selection signature, Whole-genome sequencing

## Abstract

**Background:**

The eight phenotypically distinguishable indigenous chicken breeds in Guizhou province of China are great resources for high-quality development of the poultry industry in China. However, their full value and potential have yet to be understood in depth. To illustrate the genetic diversity, the relationship and population structure, and the genetic variation patterns shaped by selection in Guizhou indigenous chickens, we performed a genome-wide analysis of 240 chickens from 8 phenotypically and geographically representative Guizhou chicken breeds and 60 chickens from 2 commercial chicken breeds (one broiler and one layer), together with 10 red jungle fowls (RJF) genomes available from previous studies.

**Results:**

The results obtained in this present study showed that Guizhou chicken breed populations harbored higher genetic diversity as compared to commercial chicken breeds, however unequal polymorphisms were present within Guizhou indigenous chicken breeds. The results from the population structure analysis markedly reflected the breeding history and the geographical distribution of Guizhou indigenous chickens, whereas, some breeds with complex genetic structure were ungrouped into one cluster. In addition, we confirmed mutual introgression within Guizhou indigenous chicken breeds and from commercial chicken breeds. Furthermore, selective sweep analysis revealed candidate genes which were associated with specific and common phenotypic characteristics evolved rapidly after domestication of Guizhou local chicken breeds and economic traits such as egg production performance, growth performance, and body size.

**Conclusion:**

Taken together, the results obtained from the comprehensive analysis of the genetic diversity, genetic relationships and population structures in this study showed that Guizhou indigenous chicken breeds harbor great potential for commercial utilization, however effective conservation measures are currently needed. Additionally, the present study drew a genome-wide selection signature draft for eight Guizhou indigenous chicken breeds and two commercial breeds, as well as established a resource that can be exploited in chicken breeding programs to manipulate the genes associated with desired phenotypes. Therefore, this study will provide an essential genetic basis for further research, conservation, and breeding of Guizhou indigenous chickens.

**Supplementary Information:**

The online version contains supplementary material available at 10.1186/s12864-023-09621-w.

## Background

Chicken (*Gallus gallus*), the most widely distributed livestock species globally, is valuable for providing animal protein to the increasing human populace and also can be used as an excellent animal model for scientific research [1]. Since the domestication of the red jungle fowl, hundreds of phenotypically distinguishable domestic chicken breeds have diverged under the combined effects of natural and artificial selection which were subjected to wide variety of conditions [2]. These are important factors that shaped strong genome diversity, leading to significant phenotypic changes and distinct features, such as differentiation in body size, plumage color, comb type, flying ability, and environmental adaptability among chicken breeds [3]. Analysis of genetic data and patterns enables identification of genetic variants in relation to specific features within the chicken breeds [4]. In the past decades, several genomic studies have investigated the genetic variation that underline the characterization of specific traits in various chicken breeds [5–7].

China is one of the largest producers of domestic chickens worldwide, and has accumulated multiplex indigenous chicken sources based on its multiplex geography and culture [8, 9]. During a long period of domestication and selection, Chinese indigenous chickens have developed considerable genetic variation and phenotypic diversity in morphology and physiology [10]. Therefore, a comprehensive and detailed understanding of the genome diversity and structure of these chicken breeds could help to reveal their population dynamics and the genetic mechanisms underlying several characteristic features of these chicken breed, thereby providing theoretical basis to facilitate effective poultry breeding programs.

Guizhou province is located in the mountainous terrain of Southern China, and is characterized by a special climate and varied ethnic cultures making it a favorable geographical location for developing unique and rich domestic chicken resources, which can be good resources for new chicken breeds. The current version of a Chinese official survey – China National Commission of Animal Genetic Resources, 2021 – showed that eight indigenous chicken breeds originating from Guizhou province exhibited desirable characteristics, such as environmental adaptability, high disease resistance, high meat quality, and high genetic diversity [11]. Among these indigenous chicken breeds, Qiandongnan xiaoxiang chickens (QD) and Xingyi aijiao chickens (XY) exhibit desirable characteristics such as compact bodies, good foraging ability and environmental adaptability. Furthermore, the characteristics features of the Wumeng black-bone chickens (WM) and Chishui black-bone chickens (CSWG) are black feathers, high quality meat, and good value for pharmaceutical purposes. Puding gaojiao chickens (PD) are characterized by large body size, higher fat deposition and good quality meat. Changshun green egg chickens (CS) are famous for high quality green egg production. Yaoshan chickens (YS) are characterized by large body size and tender meat. Weining chickens (WN) are well known to be adapted favorably to high-latitude zones and cold winters. Taken together, these native chicken breeds show similar desirable features, such as good environmental adaptability, high disease resistance, good meat quality, and high genetic diversity. These desirable features make these native chicken breeds valuable experimental models for exploring the genetics mechanisms underlying their domestication, adaptation, as well as their unique characteristics, as it involves the transformation of the ancestral red jungle fowl into a domesticated chicken [12]. Furthermore, parallel populations of commercial strains were independently established from the earlier multipurpose domestic populations to meet the increasing demand for poultry meat and eggs [13], however this has threatened some native breeds, including some Guizhou local chicken breeds [14]. Therefore, it is urgent to formulate and implement exploitation plans, as well as effective conservation measures for these Guizhou local chicken breeds.

Effective utilization and conservation of these domestic chicken resources depend on accurate assessment of their genetic diversity and structure [15]. Several previous studies have reported the the genetic diversity and structure of Guizhou native chickens [11, 16, 17], however their specific phenotypic characteristics and diverse genetic resources require further elucidation. Therefore, in this present study, whole genome re-sequencing was conducted and genome-wide single nucleotide polymorphisms (SNPs) of 300 domestic chickens (including 240 domestic chickens from eight Guizhou local chicken breeds, and 60 commercial chickens from two common broiler and layer breeds) were generated. The genomic data of 10 red jungle fowls (RJF) in a previous study were included in this analysis. This present study will provide deep insights into the genetic diversity, relationships and population structures, and signature selections within the Guizhou nationwide indigenous chicken breeds. Furthermore, it will furnish useful genetic information for establishing further breeding strategies, the genetic basis of important economic traits, and information on formulating effective conservation programs of domestic chicken resources.

## Results

### Sequencing variants among different chicken breeds

In this study, 300 chickens from eight domestic breeds across Guizhou province and two commercial breeds were individually sequenced, corresponding to an average of 12.89 × coverage per individual. A total of 4201.19 Gb of high-quality genomic data were acquired with an overall mapping rate of 99.15% (Tables S1 and S2). After the accurate quality filtering of the identified SNPs, 8,145,040 high-quality SNPs were identified in the 300 individuals with the transition/transversion ratios (Ts/Tv) of 2.667 to 2.695 in each population, proving the high quality and validity of the SNP data. From the total SNPs, 84.24% were aligned to intronic and intergenic regions, whereas only 2.37% were located in the exons’ regions, and most of these SNPs were homozygous SNPs (69.23%). In addition, among the coding SNPs, 79,045 synonymous SNPs and 30,825 nonsynonymous SNPs were observed, representing a nonsynonymous/synonymous ratio of 0.39. Moreover, it was observed that all the SNPs were distributed evenly on most of the chromosomes except on chromosomes Z and 33 (Figure S1). For each population, the Guizhou indigenous chickens (except for XY) contained higher number of SNPs than that of RJF, whereas Shanghai xinyang layers (SHXY) exhibited the lowest number of SNPs (Table S3). Furthermore, compared with the chicken SNP data from the Ensembl database (http://ftp.ensembl.org/pub/release-104/variation/gvf/gallus_gallus/), 4% to 7% novel SNPs were identified in different populations, with the lowest number found in the commercial populations. Consistently, most of the novel SNPs in each population were located in the intronic and intergenic regions (Table S4).

### Genetic diversity among different chicken breeds

Table 1 showed the summary of the genetic diversity parameters for the different chicken breeds. It was observed that the Na values ranged from 2.23 for XY to 2.41 for CS, whereas, the Ne values ranged from 1.60 for SHXY to 1.73 for CS. And the range of PIC values in all chicken breeds was 0.28 ~ 0.32.

**Table 1:** Polymorphism for ten chicken breed populations

Group	Na	Ne	PIC	Pi	Ho	He	Nei	MAF
TF	2.247234	1.608266	0.309552	0.002644	0.307283	0.286166	0.301296	0.264328
SHXY	2.248514	1.604268	0.301969	0.002670	0.302881	0.242342	0.269269	0.274423
WN	2.314859	1.710332	0.314031	0.003101	0.404421	0.316083	0.351204	0.255877
CS	2.414526	1.730915	0.324503	0.003068	0.364314	0.303781	0.337535	0.25346
QD	2.283256	1.640861	0.293192	0.002788	0.325442	0.284663	0.316292	0.248098
CSWG	2.31912	1.666906	0.303326	0.002886	0.341856	0.294423	0.327136	0.246335
XY	2.226753	1.607901	0.280376	0.002673	0.303072	0.272306	0.302562	0.253973
PD	2.29088	1.639407	0.283499	0.002713	0.371321	0.287464	0.319405	0.259113
YS	2.385342	1.684525	0.311457	0.002941	0.308378	0.285591	0.317323	0.247533
WM	2.290651	1.642329	0.293856	0.002797	0.311491	0.281436	0.312707	0.251296
RJF	2.335512	1.672581	0.30381	0.002973	0.275839	0.277649	0.308498	0.238092

*Na* Observed number of alleles, *Ne* Effective number of alleles, *PIC* Polymorphism information content values, *Pi* Nucleotide variability, *Ho* Observed heterozygosity, He Expected heterozygosity, *Nei* Nei’s genetic distance, *MAF* Minor allele frequency

The Pi values of commercial chicken breeds such as Tianfu broilers (TF) and SHXY were lower compared to indigenous and wild type chicken breeds, and the highest Pi value was found in the WN. The Ho and He levels in the range of 0.24 ~ 0.40 indicated that all the breeds showed heterozygosity. Furthermore, it was observed that the Nei values ranged from a low of 0.27 in SHXY to a high of 0.35 in WN, whereas the MAF levels obtained in the commercial chicken breeds were higher than the other chicken breeds, with MAF of 0.26 (TF) and 0.27 (SHXY).

**Fig. 1:**
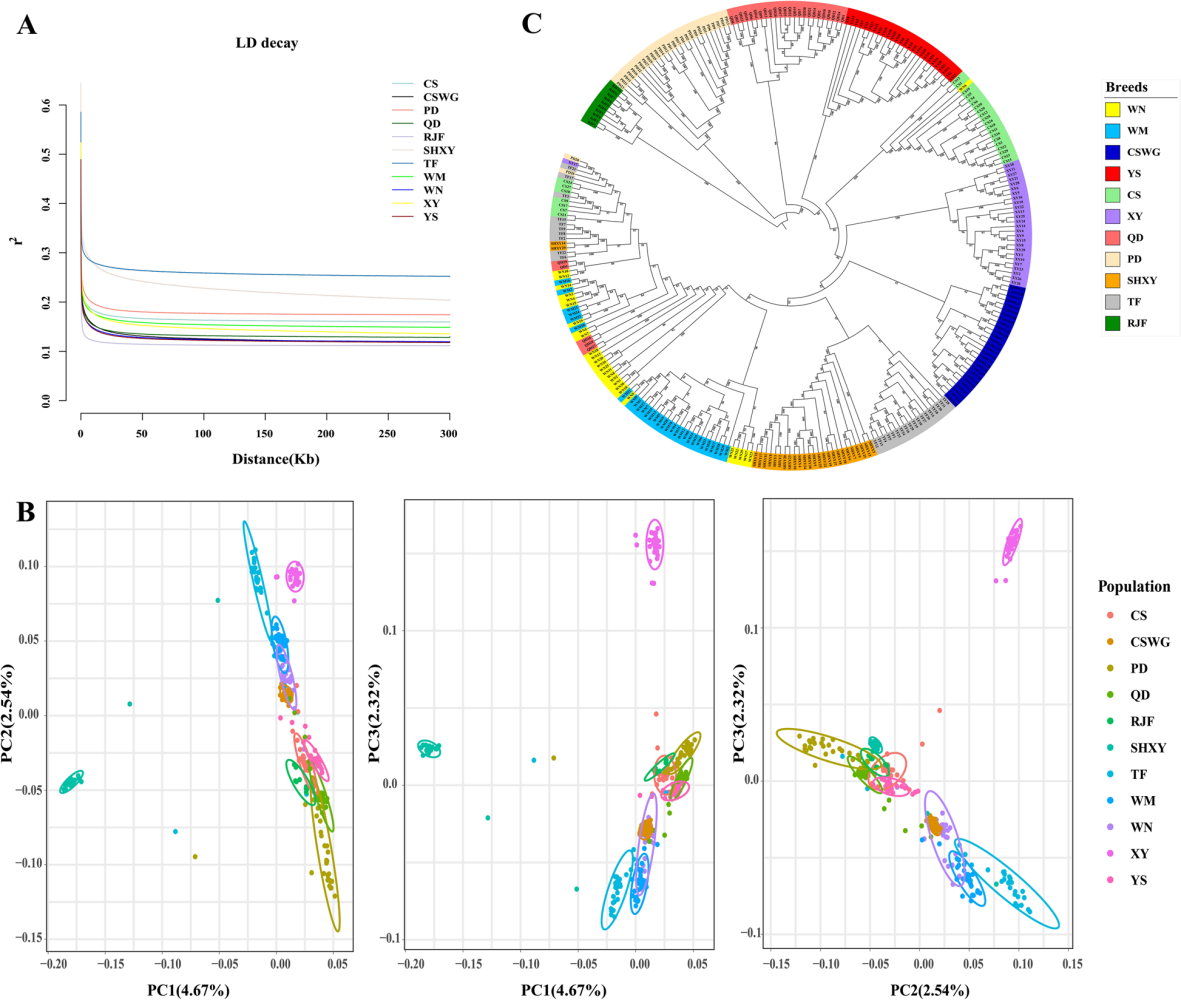
Linkage disequilibrium (LD) decay and population structure analyses. **A** LD decay of 11 chicken breed populations, denoted with one line for each population. **B** Principal component analysis (PCA), with 4.7%, 2.5%, and 2.3% variances explained in PC_1_, PC_2_ and PC_3_, respectively. **C** Neighbor-joining tree of 310 chickens

Furthermore, all the indigenous chickens obtained slower LD decay rates than those in the commercial chicken populations (Fig. 1A). The wild chicken breed (RJF) showed the slowest LD decay rate. In addition, the Guizhou native chicken breeds were divided into two groups according to the LD pattern rank, including the first cluster with faster LD decay for PD, CS, WM, and XY breeds, and the second cluster with the slower LD decay for QD, CSWG, WN, and YS breeds.

### Population genetics structure among different chicken breeds

The PCA analysis revealed that the top three PCs displayed the genetic differentiation between the commercial chicken populations (TF and SHXY) and the other chicken breeds (CS, QD, PD, XY, CSWG, WN, WM, YS and RJF) (Fig. 1B). The PC1 (4.7% variances explained totally), PC2 (2.5% variances explained totally) and PC3 (2.3% variances explained totally) divided all the chicken breed populations into five clusters. Thus, WN, WM, and CSWG were genetically similar to each other, however XY and PD were identified as two separate clusters. QD, YS, and CS were geographically close and tend to get more relative to each other, meanwhile the commercial chicken breed populations (SHXY and TF) formed an independent cluster from other breeds. In addition, the 10 RJF gathered with Guizhou indigenous chickens and away from commercial chickens. It was noteworthy that QD, PD, TF, WN, SHXY and CS have significant variations within the breed, since some individuals from these populations did not cluster together.

Additionally, the phylogenetic analysis was consistent with the PCA results. As shown in Fig. 1C, all the 300 chickens from the 10 chicken breed populations were separated into seven clusters. Taking the RJF as the root, the near cluster 1 and cluster 2 were PD and QD, whereas CS and YS were grouped as cluster 3. Here, an individual from the WN was grouped with CS. Then XY and CSWG branched into cluster 4 and cluster 5, respectively. Subsequently, the commercial populations SHXY and part of TF were arranged in the tree, forming a visually distinct cluster. Cluster 7 consisted of WN and WM, and it was difficult to separate these two breeds into their own clade. Moreover, parts of QD were belonged to the cluster 7. Consistent with the analysis of PCA, PD, TF, SHXY, XY and CS chickens were not well-grouped in the tree, as some of the individuals from these breeds were clustered together at the end of the tree.

**Fig. 2:**
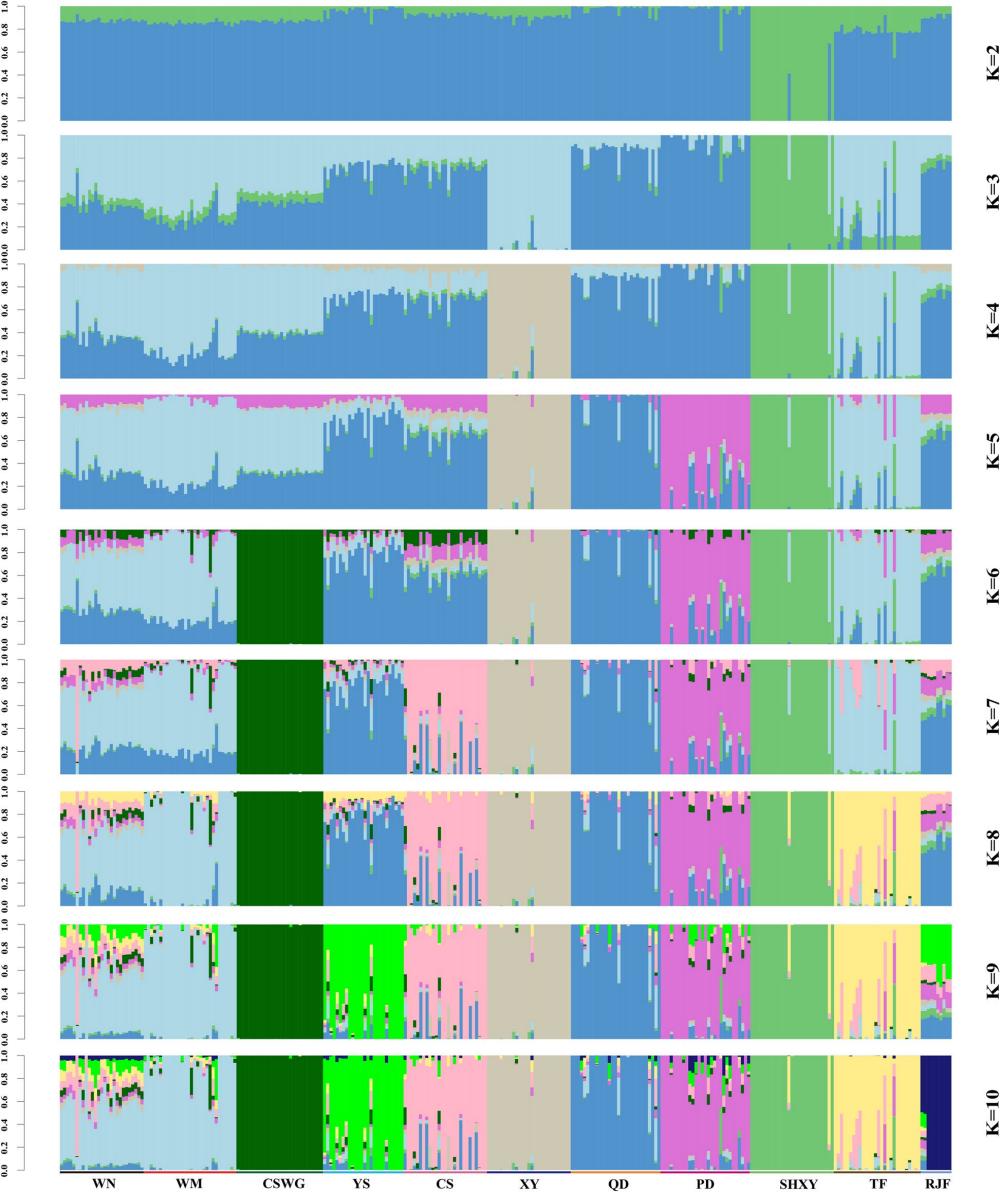
Admixture analysis with K values running from 2 to 10

The ADMIXTURE analysis with the K values running from 2 to 10 is showed in Fig. 2. At K = 3, genetic divergency first occurred between commercial breeds and indigenous ones, and we observed that the Guizhou indigenous breeds shared the same ancestral lineage with the RJF and SHXY chicken breeds. When the K ranged from 3 to 4, a potential widespread genetic exchange occurred from the commercial populations to the Guizhou indigenous ones, except PD and XY. The indigenous chickens were gradually separated from each other when K ranged from 5 to 10. Moreover, a potential widespread of genetic exchange between the Guizhou indigenous chickens during the population evolutionary process were clearly observed, however WM and WN chickens as well as YS and QD chickens showed the same ancestral components. At the best fit (K = 7), Guizhou indigenous chicken breeds showed six ancestral components that were different from the other chicken breeds, which corroborated the findings of both PCA and the neighbor-joining tree.

#### Genome wide genetic differentiation among different chicken breeds

The results of the genetic differentiation for the different chicken breeds are showed in Table S5. The pairwise Fst values ranged from 0.01 (PD-WN) to 0.18 (YS-SHXY). Moreover, the gene flow (Nm) among all the breeds in the present study ranged from 1.16 between PD and WN to 24.23 between YS and SHXY, which is consistent with the results of pairwise Fst. Notably, most of the pairwise Fst values between the commercial and indigenous breeds were in the range of 0.15 ~ 0.25. Among the 8 native chicken breeds, the pairwise Fst values were observed in relatively high levels between the YS breed and some other breeds, implying a relatively high genetic differentiation between these chicken breeds. On the contrary, these values were less than 0.05 between PD and WN breeds with some other native breeds. In addition, the pairwise DR values were in the range of 0.018147 (WM-WN) to 0.123142 (SHXY-RJF). Similarly, the pairwise DR values between the commercial and indigenous breeds were relatively high, especially for SHXY chickens.

#### Gene flow among different chicken breeds

**Fig. 3:**
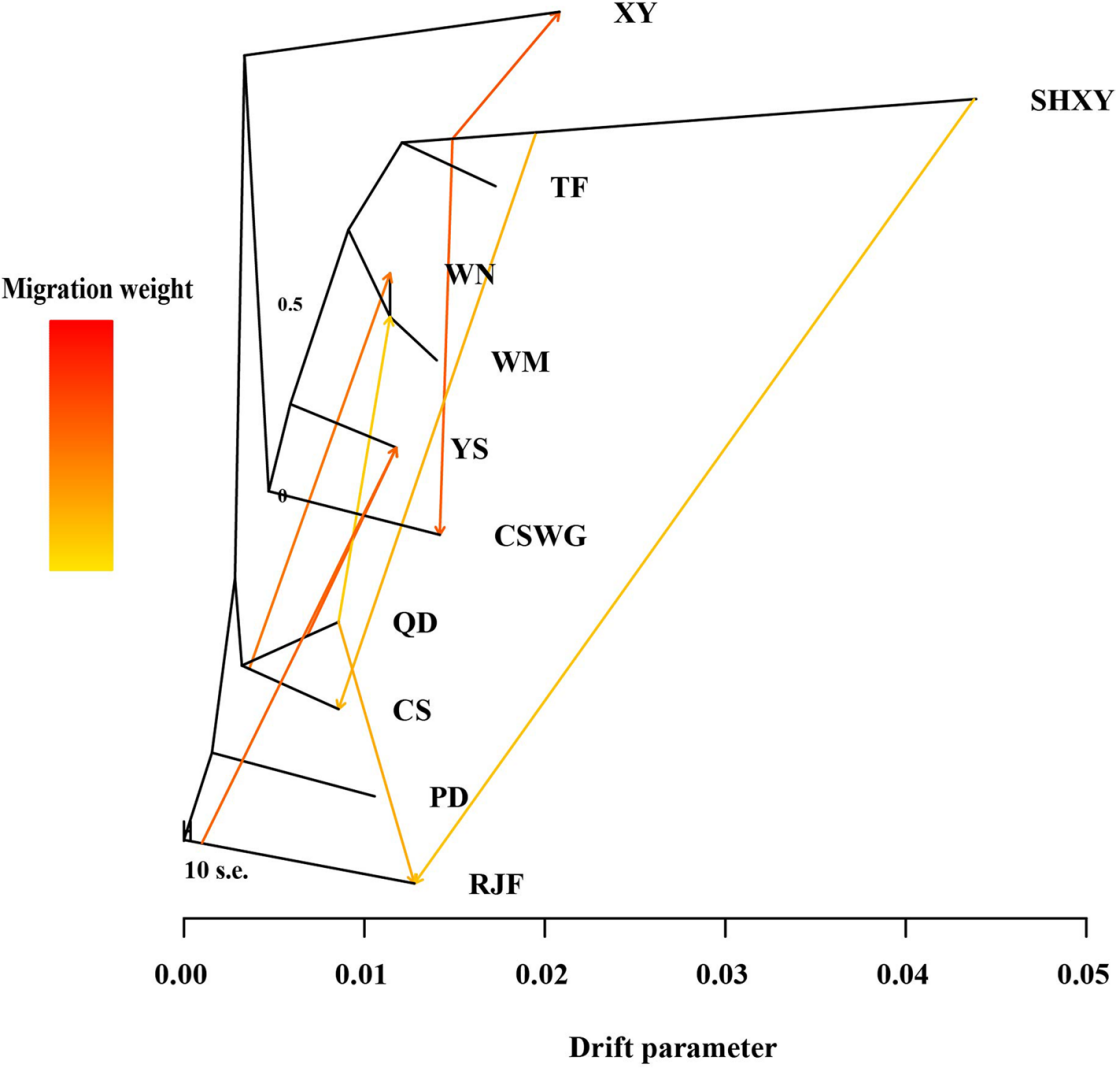
TreeMix analysis of Guizhou indigenous and commercial chickens. This maximum likelihood tree explained 99.5% variance after adding nine migrations. Migration weight was given according to the color of the arrows. The scale bar denoted 10 times the average standard error of the entries in the sample covariance matrix

The maximum likelihood (ML) tree reconstructed by TreeMix further revealed the historical relationship within the 11 populations, and it allowed both split and migration events. The smallest residual was returned at 9 inferred migration edges (Figure S2). The ML tree in Fig. 3 showed that three gene flows from SHXY chickens into three Guizhou indigenous breeds, including XY, CSWG, and CS chickens, which confirmed with the Admixture results of a potential widespread exchange from the commercial chicken breeds into indigenous chicken breeds. In addition, three gene flows were observed within the Guizhou chickens, including two gene flows from QD into WN and WM, and a gene flow from CS into WN. It was revealed that among the three gene flows observed between RJF and other populations, one of them showed the gene flow from RJF into YS, whereas two of them indicated that the gene flows from other populations into RJF.

#### Genetic relationships among different chicken breeds

**Fig. 4:**
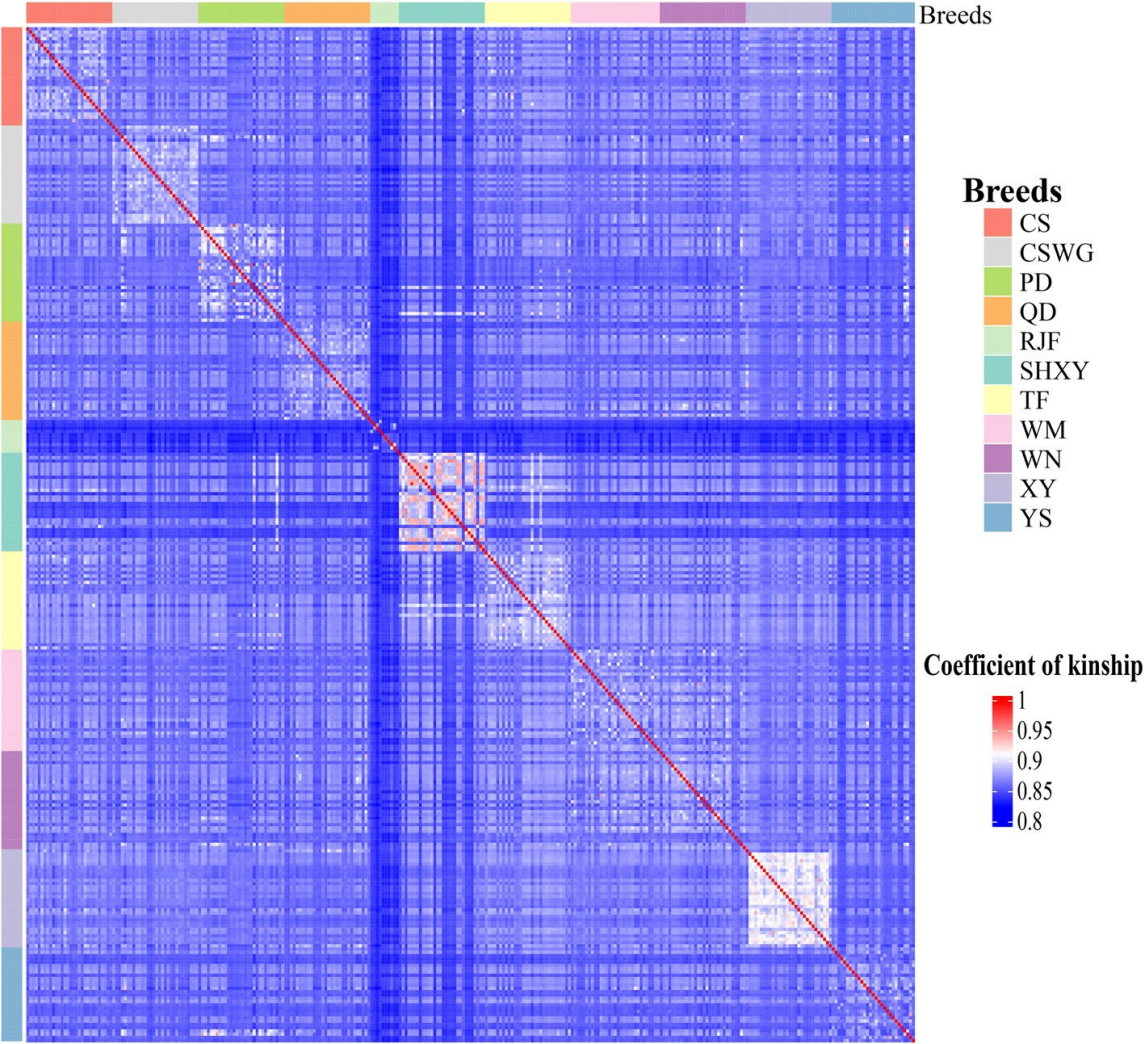
Identity-by-state analysis of 11 chicken populations. Genetic relationships were given according to the color of the squares

The results obtained from the identity-by-state (IBS) analysis showed the genetic relationships among the different chicken breeds. As shown in Fig. 4, among all the Guizhou indigenous chicken breeds, close genetic relationship was only found in partial individuals of XY, PD and CSWG populations and a minority of the individuals in YS, CS, WN, WM, and QD populations, whereas most of the individuals in the commercial populations showed close genetic relationships. Consistent with the results of the TreeMix analysis, YS showed the closest genetic relationship with RJF. Moreover, compared with the relationships within the Guizhou native chicken breeds, we observed relatively far relationships between SHXY and Guizhou indigenous chicken breeds.

#### Genome-wide selective sweep signals for domestication traits in Guizhou indigenous chicken breeds

**Fig. 5:**
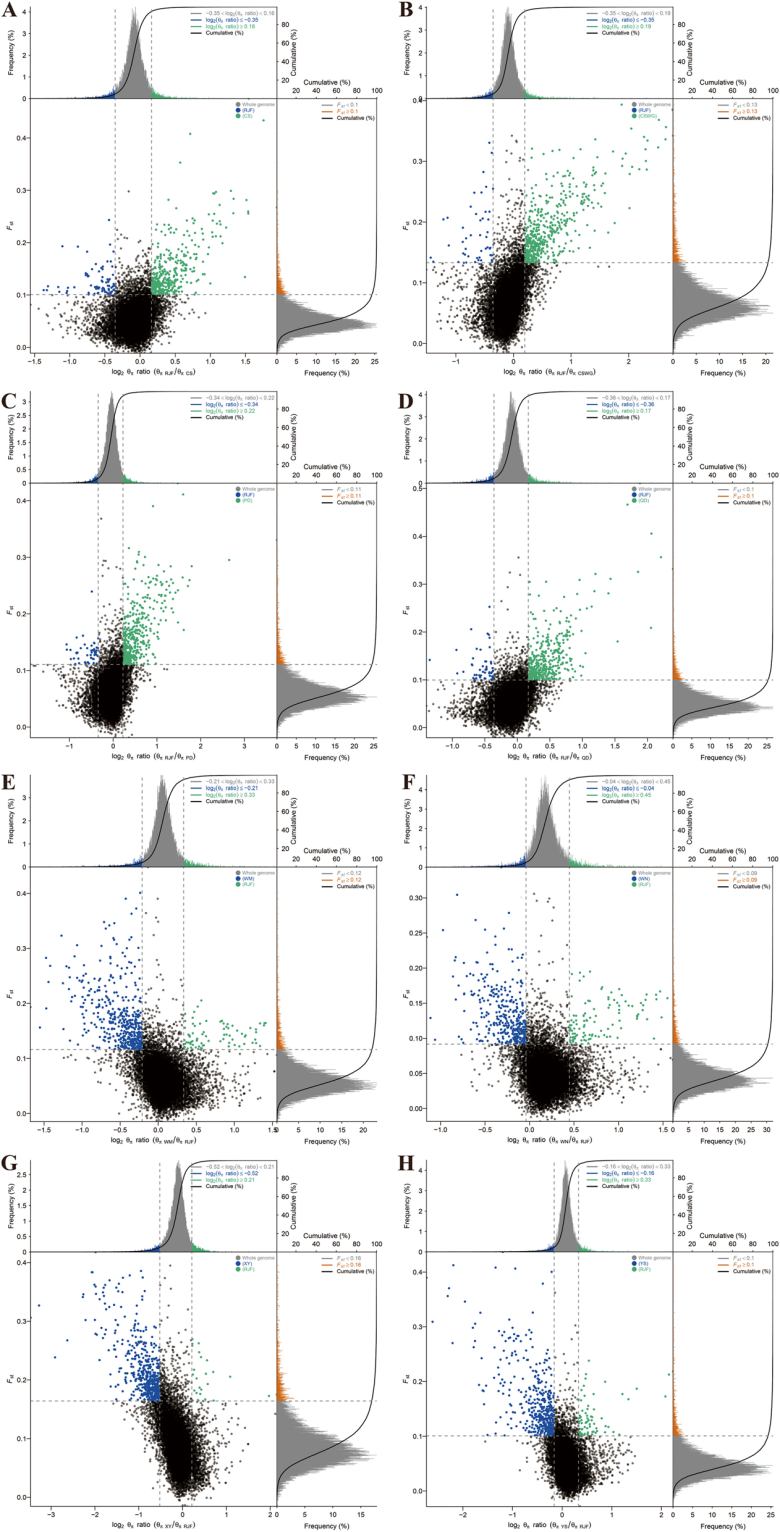
Selected regions in Guizhou indigenous chicken breeds screened by Fst and θπ method. The abscissa corresponded to the frequency distribution map above (the ratio of π_RJF_/π_object breed_), and the ordinate corresponded to the frequency distribution map on the right (Fst value). Moreover, the middle parts represented the corresponding Fst and θπ ratio in different windows, and the blue and green areas were top 5% areas selected by θπ while the areas in orange were the top 5% areas selected by Fst. **A** is for CS, **B** is for CSWG, **C** is for PD, **D** is for QD, **E** is for WM, **F** is for WN, **G** is for XY, and **H** is for YS

**Fig. 6:**
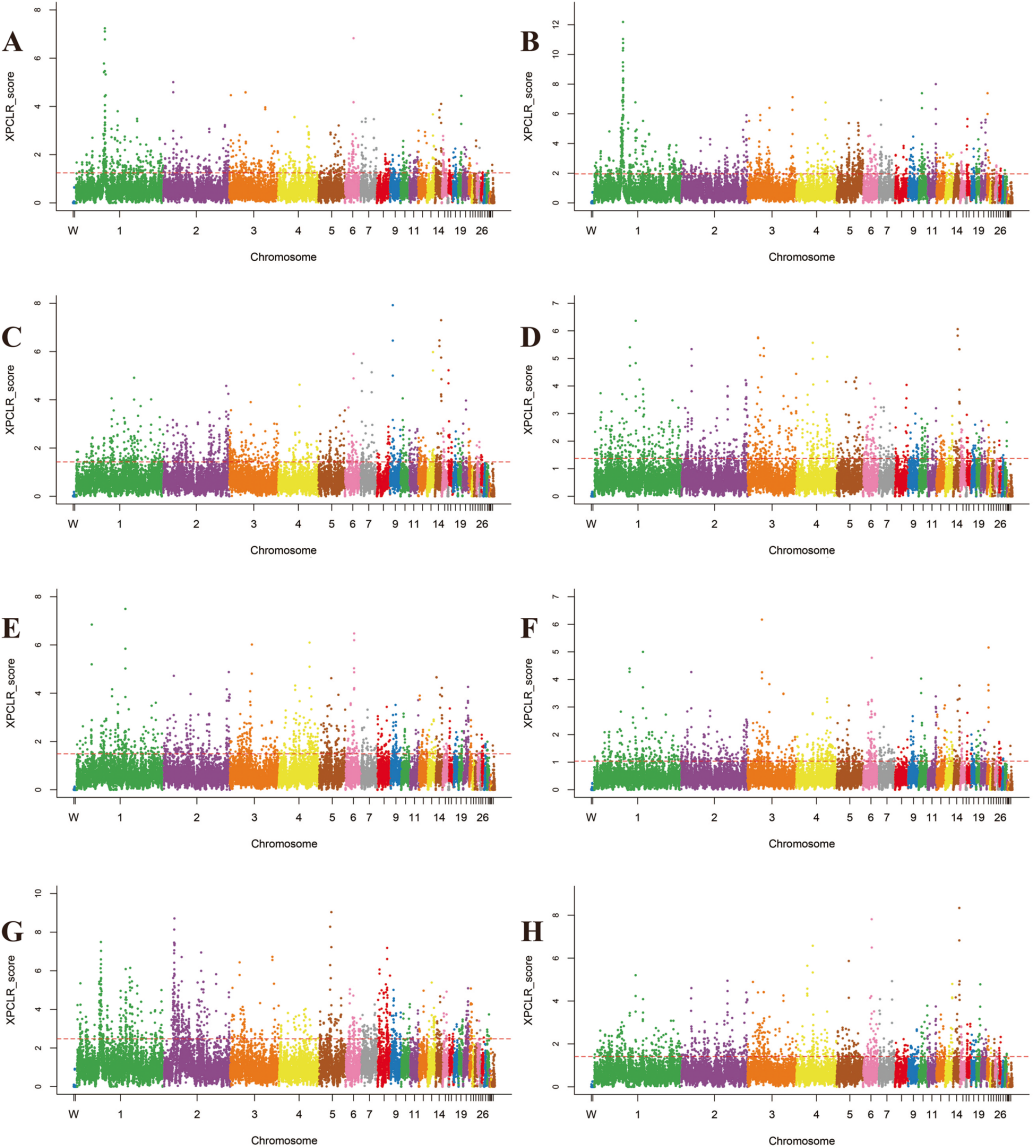
Selected regions in Guizhou indigenous chicken breeds screened by XP-CLR method. The top 5% positive selective areas by XP-CLR method. **A** is for CS, **B** is for CSWG, **C** is for PD, **D** is for QD, **E** is for WM, **F** is for WN, **G** is for XY, and **H** is for YS

**Fig. 7:**
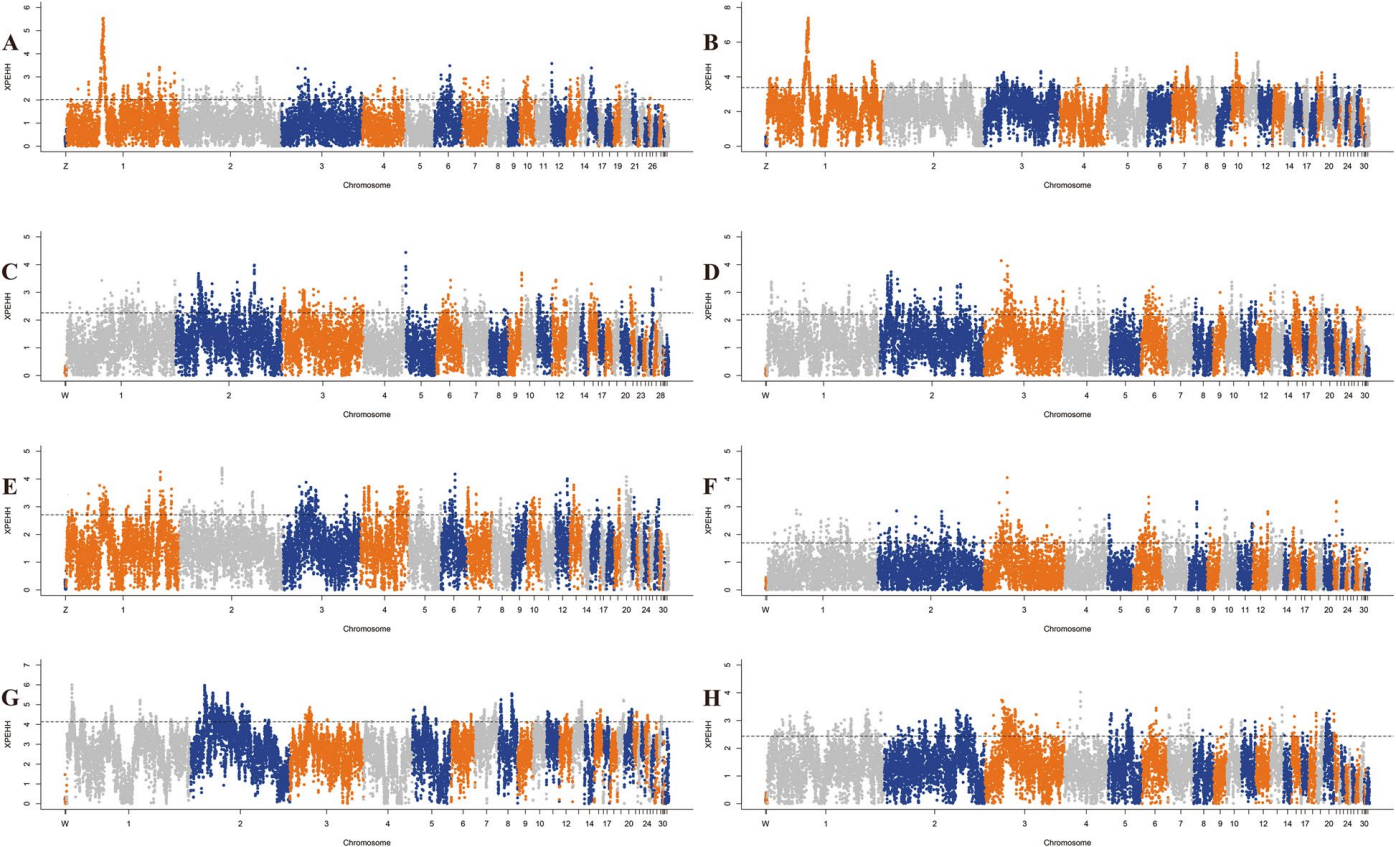
Selected regions in Guizhou indigenous chicken breeds screened by XP-EHH method. The top 5% positive selective areas by XP-EHH method. **A** is for CS, **B** is for CSWG, **C** is for PD, **D** is for QD, **E** is for WM, **F** is for WN, **G** is for XY, and **H** is for YS

To accurately detect the genomic footprints left by both intense natural and artificial selection pressure during domestication, we performed pairwise comparisons of the genome-wide variation between the wild chicken breed (RJF) and eight geographically close but phenotypically diverse Guizhou indigenous chicken breeds (CS, QD, PD, XY, CSWG, WN, WM, and YS). For CS, the inferred genome variation that was subject to a selective sweep consisted of 1106, 2515, and 3353 genes by Fst&θπ (Fig. 5), XP-CLR (Fig. 6), and XP-EHH (Fig. 7) methods, respectively. Among them, a total of 391 genes were shared by all the methods, therefore, they were regarded as the candidate genes for further analysis. Consistently, it was observed that in other Guizhou indigenous chicken populations, unequal numbers of overlapped positive selective genes (332 to 488) (Table S6) located in both the top 5% Fst&θπ selective regions, XP-CLR selective regions, and XP-EHH selective regions (Fig. 4). Additionally, further gene function annotation analysis (GO and KEGG) revealed that many genes in the genomic regions with strong selective sweep signals were involved in growth, metabolism, immunity, disease resistance and biosynthesis and may potentially contribute causally to their specific phenotypic characteristics formed during domestication.

#### Selection in the CS breed

The putative candidate genes found in CS were significantly enriched in 58 terms (*P* < 0.05), and 21 terms had extreme significance (*P* < 0.01), encompassing terms of biological processes (BPs), molecular function (MFs), and cellular component (CCs) (Figure S3). Among them, most of the BPs were associated with apoptosis, I-kappaB kinase/NF-kappaB cascade, and DNA catabolic or protection processes, containing *MUSTN1*, *CD2AP*, *DDX60*, *GLRA2*, *LCP2* and *RBM48*. And the MFs with enrichment of *LICH*, *RBM48*, *CD2AP*, *KS6B1*, *TCPE*, *BAZ1A*, *LRRF2*, *AKIB1*, *ACHA7*, *LEG8*, *CHST9*, *CXAR* and *RBM48* genes were mainly associated with some bio-binding processes and oxidoreductase activity. Moreover, those candidate genes in CS were significantly enriched in 13 KEGG pathways (*P* < 0.05), which were mainly related to glycometabolism, energy metabolism, amino acids metabolism, immunity, and smooth muscle contraction (Figure S4 and Table S7). The selected gene (*SLCO1B3*) was observed in the top ten selected regions of both XP-CLR and XP-EHH analysis. This therefore explained its phenotypic green eggshell eggs characteristic.

#### Selection in CSWG breed

For CSWG, selected candidate genes were significantly enriched in 44 GO terms (*P* < 0.05), and among which, 12 terms were extremely significant (*P* < 0.01) (Figure S3). It also found that some genes were significantly enriched in the BPs which were associated with ion transportation and acyl-CoA metabolic processes, including ion transport, iron ion transport, acetyl-CoA metabolic process, and acyl-CoA metabolic process. Furthermore, the KEGG analysis showed 18 highly enriched pathways (*P* < 0.05), which were mainly related to growth, energy metabolism, biological metabolism, disease-resistant and immunity. Notably, 5 genes were enriched in vascular smooth muscle contraction pathway (Table S8 and Figure S4).

#### Selection in the PD breed

Further GO analysis on the candidate genes of PD only showed 29 significantly enriched terms (*P* < 0.05), and some BPs and MFs were connected with disease-resistant and immune processes, for instance, autophagic vacuole assembly, macroautophagy, vacuole organization, hedgehog receptor activity, and interleukin-12 receptor binding (Figure S3). Furthermore, significantly enriched KEGG pathways (a total of 10) were divided into two categories, among which one was mainly related to growth and biological metabolism, whereas the other was associated with disease-resistance and immunity (Table S9 and Figure S4).

#### Selection in the QD breed

Additionally, we found that selected genes of QD were significantly enriched in 80 terms (*P* < 0.05), containing 9 extremely significant terms (*P* < 0.01). Among them, several BPs associated with I-kappaB kinase/NF-kappaB cascade and calcium ion transport attracted our concerns, including genes such as *CD2AP*, *RB27A*, *KCIP4* and *FSTL4* (Figure S3). In total, 7 significantly enriched KEGG pathways were found (*P* < 0.05), which were mainly associated with biological metabolism and immunity. In particular, *COX7R* and *RYR2* genes significantly enriched in cardiac muscle contraction pathway (Table S10 and Figure S4).

#### Selection in the WM breed

Based on the candidate genes of WM, 28 significant enrichment GO terms were found (*P* < 0.05), including BPs which were related to iron ion transport and homeostasis, regulation of various cell signals, and protein localization, as well as MFs which were connected with various enzyme activity, metal ion transporter activity, and immune pathway-related receptor activity (Figure S3). Furthermore, 11 significantly enriched KEGG pathways could be divided into four categories, including growth, biosynthesis and metabolism, function of myocardial and vascular smooth muscle, and disease resistance and immunity (Table S11 and Figure S4).

#### Selection in the WN breed

Furthermore, we performed GO and KEGG analysis on candidate genes of WN, and the results showed 64 significantly enriched GO terms (*P* < 0.05). Among them, 13 GO terms were extremely significant (*P* < 0.01), including BPs that were related to biosynthesis and metabolism, including phosphatidylserine metabolic process, phosphatidylserine biosynthetic process, organic substance metabolic process, NAD biosynthetic process, and NAD metabolic process (Figure S3). Similarly, among 9 significantly enriched KEGG pathways (*P* < 0.05), 2 pathways were associated with biosynthesis. Notably, 6 highly enriched pathways (*P* < 0.05) were connected with disease-resistance and immunity, including *DAAM2, CNBP1, GSK3B*, *KCC2G*, *PARD3*, *CADH1*, *CTNA2*, *GSK3β*, *GLI3*, *FAN1*, *FANCA*, *CENPS*, *SDF1*, *TNFSF13B*, *IGFBP3*, *CDK6*, and *TNR6* genes (Table S12 and Figure S4).

#### Selection in the XY breed

In total, 29 significantly enriched GO terms were observed based on the candidate genes of XY (*P* < 0.05), and among them nine enriched GO terms were extremely significant (*P* < 0.01). Notably, several BPs, MFs, and CCs were connected to DNA-related processes. In addition, some MFs which were related to oxidoreductase activity were of great importance (Figure S3). For KEGG analysis, candidate genes significantly enriched in 11 KEGG pathways, and most of them were associated with glycolipid energy metabolism and disease-resistance and immunity, especially the pathway of salmonella infection (Table S13 and Figure S4).

#### Selection in the YS breed

GO terms analysis for selected genes of YS showed that a total of 29 enriched GO terms were significant (*P* < 0.05), and eight terms were extremely significant (*P* < 0.01). Interestingly, some BPs and CCs were connected with lipids, meanwhile, some MFs were associated with calcium channel regulator activity and ATPase inhibitor activity (Figure S3). Consistently, it was observed that significantly enriched KEGG pathways (a total of 7 pathways) consisted of fatty acid biosynthesis pathway and calcium signaling pathway, including *ACSL1*, *IP3KB*, *RYR2*, *ACM3*, and *CAC1G* genes. Disease-resistant and immunity-related and nucleotides related KEGG pathways were also important (Table S14 and Figure S4).

### Genome-wide selective sweep signals for phenotypic traits

#### Selection for egg production

**Fig. 8:**
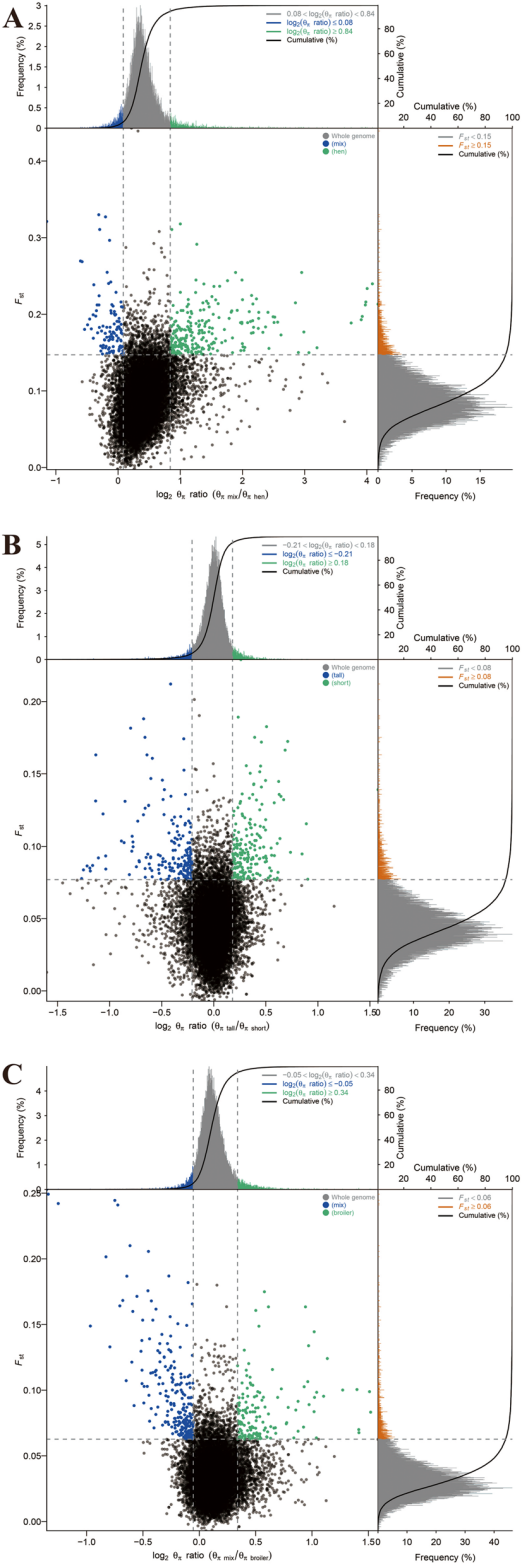
Selected regions for different traits screened by Fst and θπ method. The abscissa corresponded to the frequency distribution map above (the ratio of π_reference_/π_object breed_), and the ordinate corresponded to the frequency distribution map on the right (Fst value). Moreover, the middle parts represented the corresponding Fst and θπ ratio in different windows, and the blue and green areas were the top 5% areas selected by θπ while the areas in orange were the top 5% areas selected by Fst. **A** is for egg production, **B** is for body size, and **C** is for growth performance

**Fig. 9:**
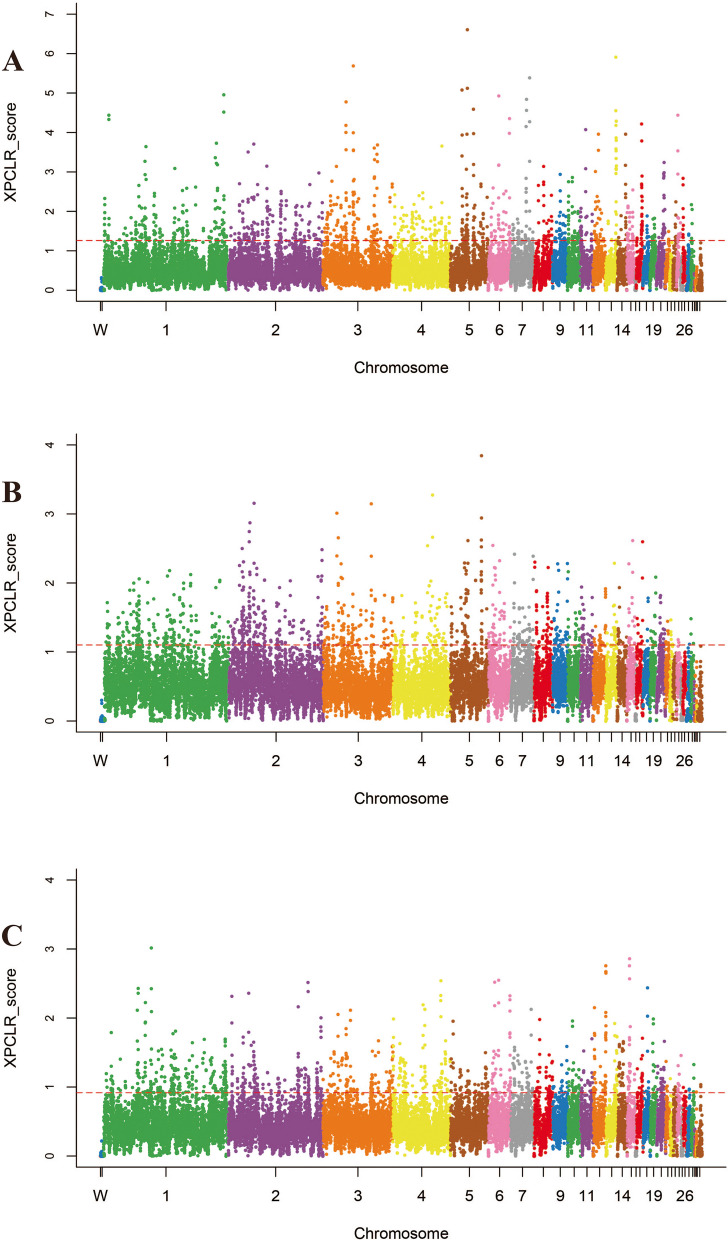
Selected regions for different traits screened by XP-CLR method. The top 5% positive selective areas by XP-CLR method. **A** is for egg production, **B** is for body size, and **C** is for growth performance

**Fig. 10:**
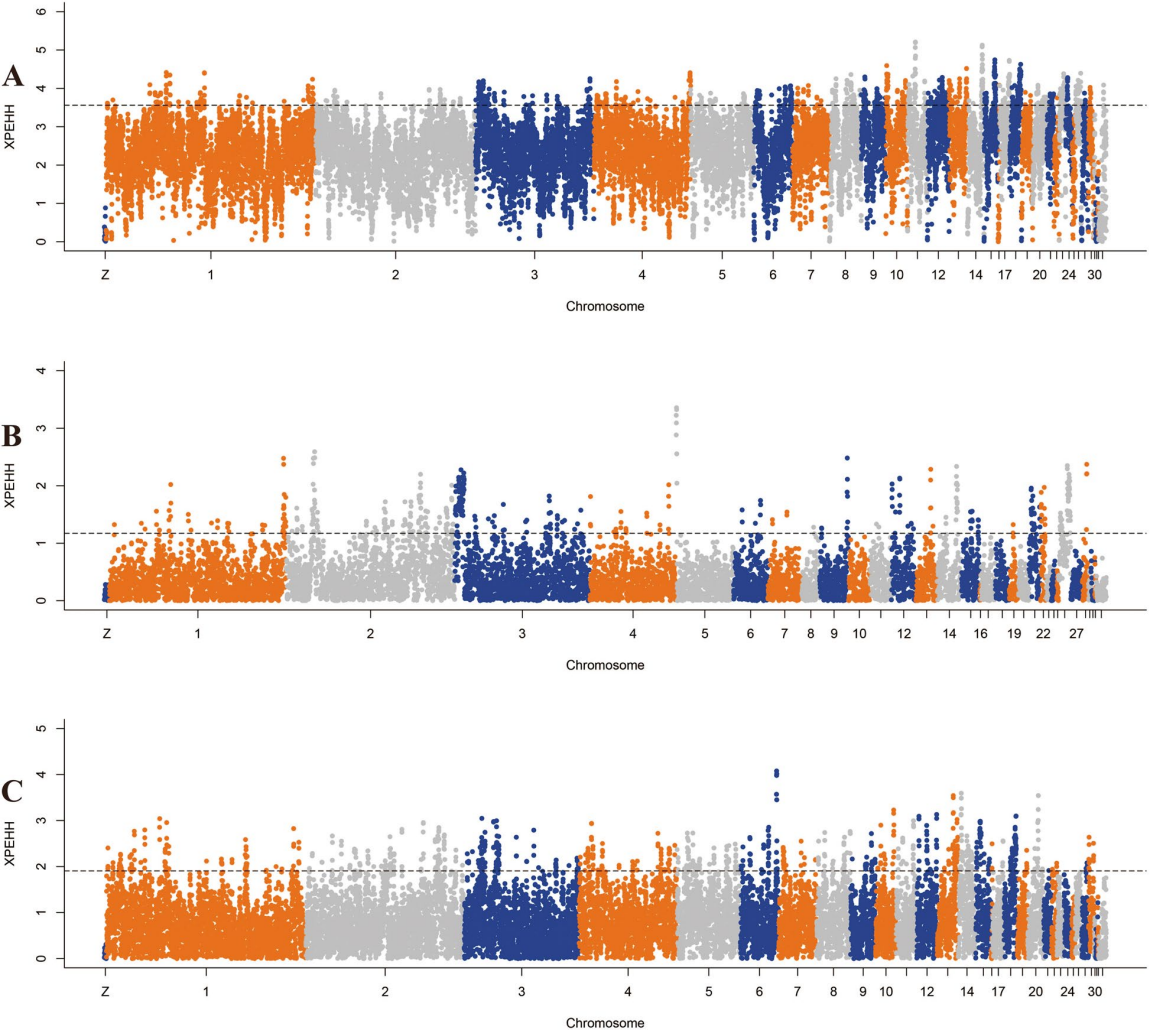
Selected regions for different traits screened by XP-EHH method. The top 5% positive selective areas by XP-EHH method. **A** is for egg production, **B** is for body size, and **C** is for growth performance

The genomic landscape of population differentiation between Guizhou indigenous chickens (reference) and commercial layer chickens (object) were explored to identify candidate genes that potentially control laying traits. Selected regions observed by Fst&θπ (Fig. 8), XP-CLR (Fig. 9), and XP-EHH (Fig. 10) methods contained 1369, 3868, and 3445 genes, respectively. Among them, a total of 435 candidate genes were shared by all methods.

Further gene function annotation on the putative candidate genes were extremely significantly enriched in 31 GO terms (*P* < 0.01), including some BPs and MFs (Figure S5). In particular, numerous biosynthetic processes and metabolic processes were significantly enriched by some candidate genes, including *SETD6*, *PSME4*, *LHX6*, *PP2BB*, and *3HAO*. Most candidate selective genes were enriched in MFs, especially on transferase activity. The results from the KEGG pathways analysis showed 7 significantly enriched pathways (*P* < 0.05), and importantly, candidate selective genes, including *AGRP*, *ACSL6*, and *PRKAB1* were significantly enriched in the adipocytokine signaling pathway (Table S15 and Figure S6).

#### Selection for growth performance

To improve our understanding of the biological mechanisms controlling important traits in modern commercial broiler chickens, we detected selected regions between Guizhou indigenous chickens (reference, except in PD which is a meat type chicken breed) and commercial broiler chickens (object). In total, 941, 2739 and 3326 positive selective genes were identified in terms of Fst&θπ, XP-CLR, and XP-EHH methods (Fig. 5), respectively, encompassing 324 candidate genes.

Further analysis showed a total of 66 significantly enriched GO terms (*P* < 0.05), whereas, only 19 enriched terms were extremely significant (*P* < 0.01), including 13 terms of BPs, 2 terms of MFs, and 4 terms of CCs (Figure S5). Importantly, most BPs connected to positive regulation of metabolic or biosynthetic processes, containing *TXD12* and *STRAB* genes. For KEGG pathways analysis, candidate genes were significantly enriched in 10 pathways (*P* < 0.05) (Table S16 and Figure S6). In particular, some pathways related to growth and bioactive substance synthesis and metabolism, such as ubiquitin mediated proteolysis, selenocompound metabolism, and glycosaminoglycan biosynthesis—heparan sulfate/heparin, containing candidate genes, including *RHBT1*, *VHL*, *SMUF2*, *FBXW8*, *TRXR1*, *XYLT1*, *CHSTB*, *CDK1*, *MD1L1*, and *ADCY8*.

#### Selection for body size

Among all the chicken breeds examined in this present study, XY and QD showed similar appearance in terms of compact bodies, whereas PD is famous for large body size and high fat deposition. Thus, we compared XY and QD (reference) with PD (object) and scanned the whole genome for selected regions related to fat deposition and body size. The results showed that a total of 1233, 2694 and 3741 genes with selection signals were identified using Fst&θπ, XP-CLR, and XP-EHH methods, respectively. Strikingly, only 116 overlapped genes were recorded as candidate genes (Fig. 5).

GO terms analysis for the above candidate genes showed 60 significantly enriched GO terms (*P* < 0.05), containing 14 terms with extreme significance (*P* < 0.01) (Figure S5). Among them, extensive BPs related to metabolic and biosynthetic processes and numerous MFs connected to various enzyme activities were of great importance, including *PTSS2*, *RET7*, *KIF1A*, *AKIB1*, and *3HAO*. In addition, a total of 8 significantly enriched KEGG pathways were found, among which some were associated with protein synthesis and hydrolysis with *UBE4B*, *BIRC6* and *STT3B* genes which attracted our concern (Table S17 and Figure S6).

## Discussion

Chinese indigenous chickens with considerable genetic variation and phenotypic diversity are important resources for the development of new breeds, and deep comprehension of genetic trajectories of chicken domestication. Thus, in this study, we estimated the genetic diversity, phylogenetic relationships and signature selections of Guizhou indigenous chicken breeds using SNPs generated from whole-genome re-sequencing. Meanwhile, the genomic data of two commercial chicken breeds and wild RJF were also included in the analysis of this study, aiming to identify potential candidate genes for important economic traits in chicken. In addition, this study was the first to draw genetic trajectories of Guizhou domesticate chicken breeds in China.

In this study, a total of ~ 8.15 million SNPs were identified based on the vast genome data obtained, which helped us to uncover the genetic architecture of these Chinese indigenous chicken breeds. Compared with the previous study using 50 K single nucleotide polymorphisms in Guizhou indigenous chickens [11], the number of SNPs obtained in this study was approximately large dozens of times or more, perhaps because of the development of the genome sequencing and multiple sampling populations. However, the advancement in technology along with comprehensive databases may contribute to gradually decreasing the proportion of novel SNPs. Consistently, our study observed less novel SNPs (4% to 7%) than the previous reports [18, 19]. The lowest levels of novel SNPs were found in the two commercial populations in this present study, reflecting less genomic diversity. These results were supported by further genomic diversity analysis.

In the present study, we found that Guizhou indigenous chicken breeds contained higher genetic diversity compared to the commercial ones, presumably due to a low level of artificial inbreeding and diversified geographical environments, which were also evidenced in previous studies [12, 20]. However, the Guizhou indigenous chicken breeds also presented unequal polymorphisms among themselves, which was consistent with previous studies [10]. PIC was widely used to evaluate polymorphism in gene loci, and the PIC values in all populations were in the range of 0.25 ~ 0.5, suggesting a moderate polymorphism [21]. Genetic heterozygosity indices (Ho, He) were good measures for genetic structure differentiation, and all the populations in this study harbored a relatively high level of heterozygosity (> 0.1), indicating moderate polymorphism [12]. Furthermore, relatively higher values of Pi were recorded in the Guizhou indigenous chicken breeds compared to the commercial populations, whereas some breeds have values higher than that observed in the RJF chicken breeds, consistent with both a low degree of intensification and less intensive selection than experienced in commercial breeds [22]. The medium nucleotide diversity of RJF (0.29%) may presumably be related to the limited sample size [18]. Among all the Guizhou indigenous chicken breeds, XY and QD showed relatively low genetic diversity, and this low genetic diversity was further evidenced by the close genetic relationship within the breed detected in the IBS analysis, indicating an effective conservation of these breeds [23]. The overall level of diversity of each population was further elucidated by performing the pattern of LD decay, which proved that Chinese indigenous chicken breeds have higher genetic diversity than the commercial lines, as observed in RJF [24].

To consider the possible genetic admixture among the populations, we also performed population structure analysis. The results showed that the clustering pattern in both the PCA, neighbor-joining phylogenetic tree and ADMIXTURE analysis remarkably mirrored the breeding history and geographical distribution of Guizhou indigenous chickens, meanwhile the results may be reflective of an overall distinctive genomic architecture [13]. Compared with the commercial chicken breeds, Guizhou indigenous fowls were closely related to RJF chickens [2]. However, the structures of the particular Guizhou breeds evidenced mixture with the ancient composition of SHXY and TF, indicating that they may have been selected for improving production performance in the breeding process, which was in accordance with the previous study [19]. Additionally, the QD, PD, TF, WN, SHXY and CS chickens were ungrouped into one cluster, suggesting their complex genetic structure. This result revealed that societal development and convenient transportation may break up the correlation between the genetic and geographical distances, leading to the gene flow between individuals from different regions and breeds [25]. However, it was difficult to separate the populations of WM and WN from each other, and this sub-structuring may be attributed to their close proximity, the same breeding histories, or the common ancestor before their evolution, as described in previous reports [26, 27]. This was also evidenced by the same ancestral components in the ADMIXTURE analysis in this present study.

In addition, mutual introgression within the Guizhou chicken breeds was also suggested by the results obtained from the ADMIXTURE analysis. Furthermore, the genome wide genetic differentiation analysis was carried out to detect the admixture or migrant events happening between the breeds. Consistently, relatively low levels of Fst along with high values of Nm were apparently found between WN or PD and some other breeds, implying high homogeneity and low genetic differentiation between these breeds [28]. Moreover, the DR values between WM and WN was the lowest (0.01814), implying that these two breeds had a close genetic relationship. It is important to note that the pairwise high level of DR and low value of Fst between YS and some other breeds supported the distinctiveness of the YS breed. In comparison with the relationship within the Guizhou breeds, the genetic differentiation analysis implied that commercial breeds generally have relatively distant genetic relationships with Guizhou indigenous chickens and wild chickens [2]. The analysis of IBS provided further evidence.

Given that a potential widespread introgression from the commercial chickens to particular Guizhou indigenous breeds had been suggested by the above analysis, the ML tree induced by TreeMix analysis helped us to better understand the historical relationship within these populations. It is well-known that the common disadvantage of Chinese indigenous chicken breeds is low production performance. Thus, predictably, Chinese breeders were introducing nonnative variants to increase egg production [29]. The migration pattern from SHXY to some Guizhou indigenous chickens supported an admixture history with commercial laying hens, and this gene flow hypothesis was clearly evidenced by the above admixture analysis [10]. Furthermore, consistent with the above results, the gene flows obtained within the Guizhou indigenous chicken breeds clearly showed the genetic admixture of WN. Taken together, it was suggested that WN was a mixed breed which may have been hybridized with other Guizhou chicken breeds in recent breeding. In addition, the gene flow from RJF to YS, along with the closest genetic relationship between them as shown in the IBS analysis indicated the effectiveness of the breed protection of YS.

Variants or genes underlying phenotypic changes in the domestic chicken may likely evolve rapidly after domestication, forming specific phenotypic characteristics of different breeds. Furthermore, the regions or loci which had experienced selection will show specific features, including high population differentiation, significantly reduced nucleotide diversity levels and long-range haplotype homozygosity [30]. Therefore, based on these principles, different parameters were examined by Fst&θπ, XP-EHH and XP-CLR methods to comprehensively identify footprints of both nature and artificial selection associated with domestication of chickens in the present study [31]. The pairwise analysis of the genome sequences of the Guizhou indigenous chicken breeds and RJF showed that genes related to disease resistance and immunity were found to overlap in all the Guizhou populations, which is synonymous with a previous study on Sichuan indigenous chickens [32]. In particular, some genes such as *LCP2, GSK-3β, DAAM2, GLI3, FBX25, ITAV, ACTN2*, *PARD3* and *TNFSF13B*, *SDF-1*(*CXCL12*), *TGF-β2, NFATC1, MKNK1, FGF9*, were regarded as important immune markers of tumorigenesis in humans, which may constitute the main genetic contributors to immunity. Collectively, these potential candidate genes may be responsible for the dramatic phenotypic change of immune ability, as it had been naturally and artificially selected to improve environmental adaptability and economic value in domestic chickens. Evolution of growth traits from wild to domestic was another striking change during domestication [22]. Consistently, we realized a group of selective genes shared among most of the chicken breeds were involved in glycolipid energy metabolism, protein and amino acid biosynthesis and hydrolysis, folate biosynthesis, and dorsoventral axis formation functional categories. These genes provided potential evidence for selection of growth performance in domesticated chickens [32]. The *BCAT1* played an important role in cytoplasmic amino and keto acid metabolism, and a recent study has reported its important role in muscle development [33]. *GLI3* which is a core gene in the Wnt signaling Pathway and *IGFBP1* was reported to promote growth rate in chickens [34, 35], whereas *DHPR* and *MOCS1* genes promote neural development [36], and mutations in *MOCS1* leads to brain injury [37]. The prominent gene of dorsoventral axis formation pathway, *SPIRE2*, plays a crucial role in the development of the nervous system and intestinal tract [38] and fecundity traits in goats [39]. *ACSL1* and *AKTIP* genes may contribute causally to the balance of lipid synthesis and metabolism [40, 41].

Noticeably, trails associated with specific phenotypic characteristics in domestic chickens had been selected as trademarks establishing distinct breeds. For instance, the strong ultraviolet radiation in southern Guizhou may easily cause DNA damage to CS, the local breed there. Thus, it was speculated that the genes enriched in DNA protection, double-stranded DNA binding, and oxidoreductase activity terms may be the key factors for the strong ultraviolet and roughage resistance of CS [42]. Similarly, the extreme temperature of the distribution area of CSWG, reaching above 40 ℃, and the heavy thermal radiation may trigger misfolded proteins, further leading to serious damage to CSWG. Selected genes, such as *WWP2*, *UBE2F* and *NEDD4* play essential roles in regulating ubiquitin degradation of misfolded proteins [43, 44], thus explaining the great thermal radiation resistance in CSWG. Moreover, the homeostasis of the internal environment was also important for thermal radiation, so that the selected genes enriched in the ion transport related terms may potentially be effective in thermal radiation resistance by preventing electrolyte disorder. PD is famous for its large body size, well meat production performance and fat deposition ability, and the selected candidate genes associated with lipid synthesis and deposition, such as *PPARG* and *MARH6* [45], may be the potential factors. It is noteworthy that numerous selected genes in QD were found to enrich in GO and KEGG terms related to myocardial function, which was indispensable for locomotor behavior. The landform of QD’s regional distribution is mountainous, meanwhile breeders reared QD mainly in free-range environments, prompting the evolution of its myocardial function and locomotor behavior during domestication. The candidate selective genes *COX7R* and *FSTL4* were considered the main genetic contributors. The same selective signals were also observed in YS, which may be related to its frequent exercise in free-range environments. Some candidate genes presented in WM were involved in myocardial and vascular smooth muscle-related categories, and these genes can potentially explain the high-altitude specific adaptation of WM. Particularly, several candidate genes involved in the calcium-signaling pathway are primarily utilized in response to hypoxia adaptation of Tibetan chickens [46], Among which, *RYR2* and *CAC1S* were two important components of the calcium ion channel, exerted heart protection effects against hypoxia [47, 48]. Furthermore, numerous positive selected genes associated with energy metabolism were observed in WM, suggesting the importance of energy metabolism for maintenance of proper body temperature in the adaptation to plateau environments [32]. WN is located in chilly highlands. In the same way, positive selective genes enriched in the terms about vascular smooth muscle contraction and energy metabolism may be conducive to WN's adaptation to cold and hypoxia environments. Essentially, selected gene *ADCY1* is responsible for regulating stress and immunity in animals [49]. Surprisingly, the folate biosynthesis process which was identified to be responsible for environmental adaptation in tetraploid fish [50], was shared in both functional enrichment analysis of WM and WN, providing a new insight into the plateau adaptation of chickens. However, further biotechnological and physiological experiments are needed. For XY, massive selected genes were enriched in terms associated with lipid biosynthesis and metabolism, and this process was closely related to meat quality and egg production in chickens. Therefore, these genes may be key factors for the high egg production performance and high meat quality of XY, especially the core genes (*FABD* and *SAST*) which were associated with fatty acid biosynthesis process [51, 52]. In addition, the candidate genes enriched in lipid biosynthesis and metabolic pathways may contribute to high egg production performance and high meat quality of YS. In addition, several functional enrichment analyses exhibited that many of the genes identified in YS were functionally involved in calcium signaling categories. Since the calcium ion pathway was associated with various physiological activities, comprising bone formation, muscle contraction, and nerve conduction, the relative selection in YS may be the explanation of its comparatively strong bones and excellent locomotor ability.

Genetic variants of specific traits, especially those with commercial value, like egg production, growth performance of broilers, body size, and reproductive performance, may encourage animal breeders and even biologists, as they play major roles in both research and breeding [53]. In the present study, we employed genome-wide selective sweep analysis to study the genetic basis underlying the commercial phenotypic traits of modern chickens, including egg production, growth performance and body size. Most of the selected genes for laying traits were mainly enriched in the terms associated with glycolipid carbohydrate energy metabolism and bioactive substances synthesis, and this was not surprising, as sufficient energy and bioactive substance are key factors that promote egg production performance. The lipid synthesis and metabolism related genes *ACSL6* [40], and *AGRP* [54] and energy homeostasis related gene *PRKAB1* [55] showed great importance in improving egg laying performance of laying hens. In assessing the genetic basis underlying egg production of commercial layer breeds, we identified several selected genes associated with synthesis of immune-related substances. This may offer an interpretation for the improved bone endurance and immune performance in layer breeding, as laying chickens were commonly raised in cages with high energy feeds, becoming susceptible to lipid metabolism disorders. Furthermore, the analysis underlying growth performance of broilers suggested that genes close to synthesis and metabolism of bioactive substances terms are associated with the growth properties of commercial broilers. Animal growth is a complex life process, jointly supported by various bioactive substances and sufficient energy. The glycosaminoglycan biosynthesis—chondroitin sulfate/dermatan sulfate pathway was the prominent KEGG pathway in the analysis of broilers. In the modern broiler industry, rapid growth rate of broilers is closely related to higher incidences of leg weakness and lameness, especially tibial chondrodysplasia [56]. Given that chondroitin sulfate exerted crucial roles in the development and protection of cartilage, the genes enriched in those pathways may be responsible for regulating the balance between rapid growth and bone development in broiler breeding. In particular, *XYLT1* and *CHST11* genes are closely related to bone health [57, 58]. Body size is influenced by many genes involved in similar functional pathways. In this present study, it was revealed that selected genes related to amino acid and protein synthesis and metabolism may potentially control the variation in body size of domestic chickens. It was reported that candidate genes such as *PTSS2* [59], *RET7* [60], and *KIF1A* [61] are indispensable in animal growth.

Although this study provided useful genome-wide selection signature drafts for eight Guizhou indigenous chicken breeds and two commercial breeds, studies involving comprehensive and deep molecular biology are necessary to explore in-depth future genetic breeding of indigenous chickens. Additionally, deep average raw read sequence coverage is needed for further whole-genome sequencing analysis to reveal specialized phenotypic traits of the Guizhou indigenous chickens from the perspective of structural variations.

## Conclusion

Taken together, in this present study, a comprehensive analysis of the genetic diversity, genetic relationships and population structures, and selection signatures were characterized across eight Guizhou indigenous chicken breeds (CS, QD, PD, CSWG, WM, WN, XY, and YS), two commercial breeds (SHXY and TF) and a wild type chicken breed (RJF). The results obtained in this study revealed that Guizhou indigenous chicken breeds showed abundant genetic diversity and a great potential for utilization, however effective conservation measures are currently needed. Additionally, genome-wide selective sweep analysis provided insights into the distinct evolutionary scenarios occurring under natural and artificial selections. Various selected genes were identified as candidates for specialized phenotypic traits of Guizhou indigenous chickens and chicken economic traits. Therefore, this study will provide an essential genetic basis for further research, conservation, and breeding of Guizhou indigenous chickens.

## Methods

### Sampling and whole-genome resequencing

**Fig. 11:**
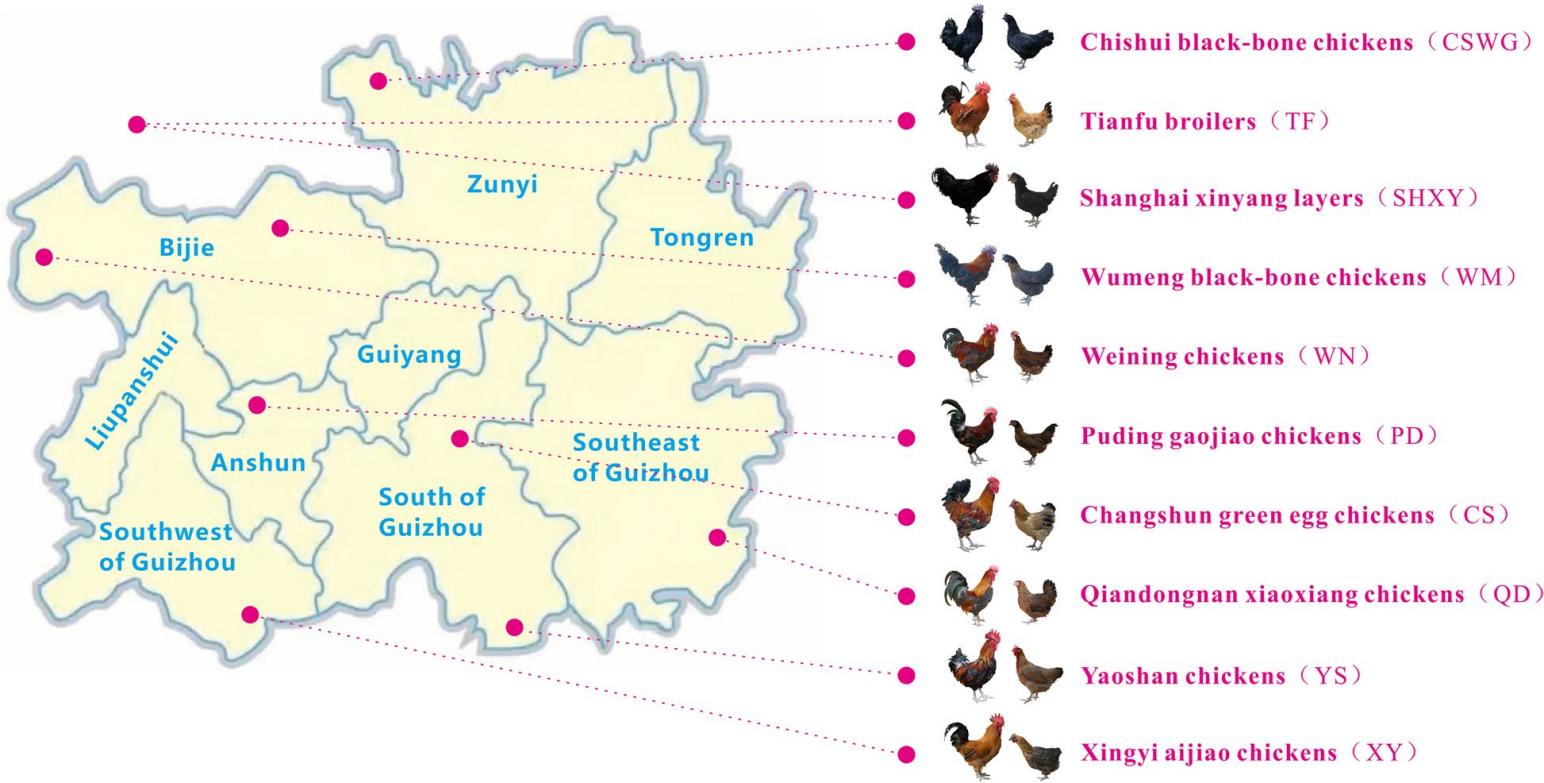
Sampling places and pictures of eight native breeds in Guizhou province and two commercial breeds

A total of 300 blood samples were collected from conservation centers or breeding farms of 10 chicken breed populations, namely eight Chinese nationwide indigenous chicken breeds from Guizhou and two typical commercial breeds, including 30 Qiandongnan xiaoxiang chickens (QD), 30 Xingyi aijiao chickens (XY), 31 Wumeng black-bone chickens (WM), 30 Chishui black-bone chickens (CSWG), 30 Puding gaojiao chickens (PD), 30 Changshun green egg chickens (CS), 29 Yaoshan chickens (YS), 30 Weining chickens (WN), 30 Tianfu broilers (TF), and 30 Shanghai xinyang layers (SHXY) (Fig. 11 and Table S18). SHXY is a commercial chicken breed mainly used for commercial egg production in China, whereas TF is a famous commercial broiler breed in the southwestern part of China, and is a three-line hybrid of a Chinese indigenous chicken source and commercial broiler breeds. Genomic DNA were extracted by a conventional phenol–chloroform method, and the quality and quantity of the DNA were determined using a Nanodrop ND-2000 spectrophotometer at 260/280 nm ratio (Thermo Fisher Scientific, Waltham, MA, USA) and by agarose gel electrophoresis. Then Paired-end (PE) sequencing libraries were constructed according to the MGIEasy Universal DNA Library Prep Set and whole-genome sequenced using MGISEQ-2000 with PE100 developed by BGI Genomics Co., Ltd. to an average raw read sequence coverage of 12.89-fold. The sequencing data of another ten red jungle fowls (RJF) were downloaded and incorporated (GenBank accession number PRJNA241474 and PRJEB30270). Description and quantification of the phenotypic diversity of each breed are shown in Table S19.

### Variant calling and annotation

After the removal of the adapter sequences (> 10 nt aligned to the adapter, allowing ≤ 10% mismatches), low-quality reads (unidentified nucleotides content ratio ≥ 10% or base ratio (Q ≤ 5) > 50%), and duplicated reads, the clean reads were retained for subsequent genome mapping and variant calling. The high quality paired-end reads were mapped onto the *Gallus gallus* GRCg6a (http://ftp.ensembl.org/pub/release-106/fasta/gallus_gallus/dna) using BWA version 0.7.15 with the command ‘mem -t 10 -k 32’ [62]. Then, the BAM alignment files were generated from the obtained SAM format files using SAMtools (version 1.3), following the command “rmdup” [63]. Then the Picard MarkDuplicates version 2.9.0 was employed to mark duplicate reads and SortSam was used to index BAM files. Then, GATK (version 4.0) [64] were utilized to perform SNP calling. The filter command 'VariantFiltration' was applied to exclude potential false-positive variant calls. In addition, SNPs with adjacency distance ≤ 5 were further removed. Meanwhile, VCFtools (version 0.1.15) [65] with the option ‘–max-missing 0.9 –maf 0.05 –min-alleles 2 –max-alleles 2’ was employed to obtain double-allelic variants. Finally, the highly accurate SNPs sites with missing data 0.05, quality value > 20, and mean depth values > 5 were kept for downstream analysis. Then, 8,145,040 SNPs were reserved after filtration from 36,003,961 raw SNPs.

Furthermore, the package ANNOVAR was employed to annotate genetic variants, classing SNPs into synonymous or nonsynonymous mutations [66]. Since the samples consisted of half males and females, we further extracted the autosomal SNPs for subsequent analyses at the genome-wide level to avoid non-stochastic effects [1].

### Genomic diversity analysis

PLINK (version 1.90) with the default parameters was used to calculate the expected heterozygosity (He), observed heterozygosity (Ho), minor allele frequency (MAF), polymorphism information content values (PIC), and Nei’s genetic distance (Nei) [67]. The effective number of alleles (Ne) and observed number of alleles (Na) for each breed was calculated using the default parameters in N_e_ESTIMATOR software (version 1.3) [68]. Then the genome-wide nucleotide diversity (Pi) was performed using VCFtools (version 0.1.13) [65]. Thereafter, PopLDdecay (version 3.40) was conducted to calculate the squared correlation coefficient (r^2^) to estimate patterns in the extent of linkage disequilibrium (LD) [69].

### Population genetics analysis

Based on the genome-wide SNPs, we performed the population structure analysis using several methods. First, the neighbour-joining (NJ) tree (bootstrap values = 1,000) was constructed to show the phylogenetic relationship under the p-distances model of the software TreeBeST (version 1.9.2) [70]. Second, the principal component analysis (PCA) was performed using GCTA (version 1.24.2) software [71]. Third, to investigate the genetic structure of all populations, ADMIXTURE (version 1.30) [72] was used to run with the group numbers from K = 2 to K = 10. The optimal K was determined by the parameter standard errors for each possible group number [73]. Here, the lowest mean CV error obtained was for K = 7 (Figure S7). Then, before the principal component analysis (PCA) and Admixture analysis were performed, all population autosomal SNPs must be LD-based pruned using PLINK (version 1.90) with the command “indep-pairwise 50 5 0.5” [1].

### Genome wide genetic differentiation analysis

The VCFtools (version 0.1.13) with parameters 100 kb sliding window and 50 kb step size was used to perform genome wide genetic differentiation analysis, including genetic differentiation (Fst), Gene flow (Nm), and genetic distance (DR) [65].

### TreeMix analysis

To further infer the historical relationships of the chicken populations included in the present study, TreeMix software (version 1.12) [74] was used with the migration events given from 1 to 10, along with their corresponding residual matrix generated following the command “-noss” and “-k 500”. Migration edge with both smallest residuals and highest value of “variance of relatedness between populations explained by the model” was maintained to construct a tree. Here, the migration event of 9 was the best fit (Table S20).

### Genetic relationship analysis

The PLINK (version 1.90) was utilized to construct an identity-by-state (IBS) matrix that was used to quantified the genetic similarity between the individuals.

### Genome-wide selective sweep analysis and enrichment analysis

To identify the genomic regions harboring the footprints of positive selection in each group of indigenous or commercial chickens, multiple selective sweep methods were used to investigate selection in the both chicken breed populations and in a single population, including coefficient of nucleotide differentiation (*Fst*), nucleotide diversity (*θ*_*π*_), cross-population extended haplotype homozygosity (XP-EHH) and cross-population composite likelihood ratio test (XP-CLR) statistical methods [31]. Here, we performed selective sweep tests for four strategies, first, RJF (reference population) and eight Guizhou indigenous chicken breeds (object population), second, Guizhou indigenous chicken breeds and layer population (SHXY), third, Guizhou indigenous chicken breeds (except for PD) and broiler population (TF), fourth, chicken breeds with small body size (XY and QD) and a chicken breed with big body size (PD) (Table S21). Fst and π values were estimated by screening the chicken genomes with a sliding window approach (a window size of 100 kb and a step size of 50 kb) [75]. *θ*_*π*_ was calculated as π_reference_/π_object_. Then the XP-EHH was performed as previously described [76]. XP-CLR test was conducted using the XP-CLR software with parameters “sliding window size 0.1 cM, grid size 10 kb, maximum number of SNPs within a window 300, the absolute pairwise correlation coefficient for two SNPs with a cut-off level of 0.99” [77].

The significance threshold was set to the top 5% for each method, and the top 5% outliers of the bins were regarded as the candidate selective regions under strong selective sweep and subsequently examined for potential candidate genes. The intersection of candidate genes associated with selective sweeps by all methods were used for further functional enrichment analysis [78], including Gene Ontology (GO) [79] and Kyoto Encyclopedia of Genes and Genomes (KEGG) pathways [80]. We received permission to use the KEGG software from the Kanehisa laboratory (http://www.kegg.jp/kegg/kegg1.html) [81]. GO terms and KEGG pathways with Bonferroni *P*-values < 0.05 and < 0.01 were considered significant and extremely significant, respectively.

### Supplementary Information

**Additional file 1. **This file includes Tables S1 to S21.

**Additional file 2. **This file includes Figures S1 to S7.

## Data Availability

The datasets presented in this study can be found in online repositories. The names of the repository/repositories and accession number (s) can be found at: https://www.ncbi.nlm.nih.gov/Traces/study/?acc=PRJNA947391&o=acc_s%3Aa (BioProject accession number: PRJNA947391). The original contributions presented in the study are included in the article/Additional files.

## Abbreviations

SNP: Single nucleotide polymorphism
QD: Qiandongnan xiaoxiang chickens
XY: Xingyi aijiao chickens
WM: Wumeng black-bone chickens
CSWG: Chishui black-bone chickens
PD: Puding gaojiao chickens
CS: Changshun green egg chickens
YS: Yaoshan chickens
WN: Weining chickens
TF: Tianfu broilers
SHXY: Commercial layers
RJF: Red jungle fowl
Na: Observed number of alleles
Ne: Effective number of alleles
PIC: Polymorphism information content values
Pi: Nucleotide variability
Ho: Observed heterozygosity
He: Expected heterozygosity
Nei: Nei’s genetic distance
MAF: Minor allele frequency
LD: Linkage disequilibrium
m: Meter
IBS: Identity-by-state
GO: Gene ontology
KEGG: Kyoto Encyclopedia of Genes and Genomes
NCBI: National Center for Biotechnology Information
