# Supplementary material for: Whole-genome sequencing revealed genetic diversity, structure and patterns of selection in Guizhou indigenous chickens
Source: BMC Genomics. 2023 Sep 26;24:570. doi: 10.1186/s12864-023-09621-w (PMC10521574; doi:10.1186/s12864-023-09621-w)
Supplement: Supplementary file 1 — Additional file 1. This file includes Tables S1 to S21. [file 12864_2023_9621_MOESM1_ESM.docx]

Table S1 The genome-wide sequencing information of samples

| Sample | Raw Base(bp) | Clean Base(bp) | Effective Rate(%) | Error Rate(%) | Q20(%) | Q30(%) | GC Content(%) |
| --- | --- | --- | --- | --- | --- | --- | --- |
| SHXY19 | 13082495400 | 13051621500 | 99.76 | 0.03 | 97.59 | 92.22 | 43.66 |
| SHXY24 | 12704806800 | 12674313000 | 99.76 | 0.04 | 96.93 | 90.05 | 44.59 |
| SHXY25 | 13417743900 | 13352546100 | 99.51 | 0.03 | 97.2 | 91.84 | 45.16 |
| SHXY16 | 13152796200 | 13121684400 | 99.76 | 0.04 | 97.04 | 90.75 | 42.48 |
| SHXY3 | 12899452649 | 12185240400 | 99.77 | 0.03 | 97.13 | 91.08 | 42.34 |
| SHXY11 | 14150687400 | 14024281500 | 99.11 | 0.03 | 97.54 | 92.11 | 47.72 |
| SHXY30 | 16620557400 | 16378187400 | 98.54 | 0.03 | 97.19 | 91.24 | 44.28 |
| SHXY17 | 13020996400 | 12277554600 | 99.61 | 0.03 | 97.5 | 92 | 46.35 |
| SHXY7 | 11353771800 | 11329569000 | 99.79 | 0.03 | 97.18 | 90.85 | 44.57 |
| SHXY20 | 11977260900 | 11944224000 | 99.72 | 0.03 | 97.76 | 92.77 | 45.2 |
| SHXY5 | 14082322700 | 13191132300 | 98.12 | 0.03 | 97.44 | 91.93 | 44.08 |
| SHXY29 | 13826446700 | 13720074900 | 99.56 | 0.03 | 97.43 | 91.97 | 43.98 |
| SHXY12 | 17775147700 | 17148096900 | 99.74 | 0.04 | 96.96 | 90.49 | 42.33 |
| SHXY6 | 13372553100 | 13351752900 | 99.84 | 0.04 | 97.03 | 90.35 | 41.9 |
| SHXY18 | 12585630600 | 12515093400 | 99.44 | 0.03 | 97.38 | 91.54 | 45.61 |
| SHXY22 | 11569536300 | 11536629900 | 99.72 | 0.03 | 97.53 | 92 | 44.15 |
| SHXY2 | 11071285100 | 9453244800 | 99.55 | 0.03 | 97.69 | 93.22 | 44.31 |
| SHXY1 | 14880948600 | 14811636600 | 99.53 | 0.03 | 97.16 | 91.73 | 44.56 |
| SHXY10 | 13777932600 | 13751164200 | 99.81 | 0.03 | 97.02 | 90.31 | 43.73 |
| SHXY8 | 12035184600 | 11880846300 | 98.72 | 0.03 | 97.48 | 91.98 | 47.62 |
| SHXY4 | 10825798149 | 11389938300 | 99.78 | 0.03 | 97.47 | 92.7 | 42.15 |
| SHXY23 | 10976926200 | 10944131700 | 99.7 | 0.03 | 97.53 | 91.97 | 45.16 |
| SHXY26 | 12095932200 | 12065082300 | 99.74 | 0.04 | 96.94 | 90.53 | 42.42 |
| SHXY15 | 11454159100 | 11590632600 | 99.55 | 0.03 | 97.12 | 90.96 | 43.68 |
| SHXY9 | 15201829200 | 15147561900 | 99.64 | 0.03 | 97.5 | 91.89 | 45.88 |
| SHXY13 | 14796457500 | 14631667200 | 98.89 | 0.03 | 96.74 | 89.91 | 42.73 |
| SHXY27 | 13323470400 | 13251989400 | 99.46 | 0.03 | 97.27 | 92.06 | 44.45 |
| SHXY21 | 17115858900 | 17079771300 | 99.79 | 0.03 | 97.24 | 91 | 42.1 |
| SHXY28 | 15660885000 | 15615517200 | 99.71 | 0.03 | 97.09 | 91.58 | 42.5 |
| SHXY14 | 11404576500 | 11537413500 | 99.68 | 0.04 | 96.64 | 89.67 | 44.06 |
| TF1 | 18399879300 | 18355887000 | 99.76 | 0.02 | 97.9 | 93.98 | 42.12 |
| TF2 | 10735246600 | 11017959000 | 99.58 | 0.03 | 97.26 | 91.47 | 44.37 |
| TF13 | 15426330300 | 15388091700 | 99.75 | 0.04 | 96 | 89.26 | 42.38 |
| TF11 | 14753135700 | 14724957600 | 99.81 | 0.03 | 97.41 | 91.66 | 42.62 |
| TF4 | 12108901200 | 12049920600 | 99.51 | 0.04 | 96.45 | 88.99 | 46.7 |
| TF15 | 13327966600 | 13254108600 | 99.61 | 0.03 | 97.19 | 91.62 | 44.5 |
| TF8 | 11173796700 | 11756097000 | 99.8 | 0.04 | 96.54 | 89.73 | 42.55 |
| TF9 | 11042724700 | 9746113200 | 99.72 | 0.04 | 96.7 | 89.53 | 44.25 |
| TF14 | 15525639900 | 15476732100 | 99.68 | 0.03 | 97.54 | 92.03 | 46.22 |
| TF6 | 10932428100 | 10886088600 | 99.6 | 0.04 | 96.78 | 90.19 | 44.24 |
| TF10 | 16262948700 | 16217293200 | 99.72 | 0.03 | 97.44 | 92.65 | 42.02 |
| TF7 | 10919002200 | 9747640200 | 99.58 | 0.03 | 96.74 | 90.1 | 44.12 |
| TF12 | 17904493500 | 17870784000 | 99.81 | 0.03 | 97.66 | 93.24 | 42.03 |
| TF3 | 12278413600 | 9619059600 | 99.63 | 0.04 | 95.87 | 86.9 | 42.92 |
| TF5 | 12385744200 | 12362020800 | 99.81 | 0.04 | 96.28 | 88.53 | 42.33 |
| TF23 | 16694098800 | 16613047800 | 99.51 | 0.03 | 96.97 | 91.03 | 45.24 |
| TF30 | 20921304000 | 20803762500 | 99.44 | 0.03 | 97.59 | 92.97 | 44.06 |
| TF25 | 17367177000 | 16848971700 | 98.26 | 0.03 | 97.68 | 92.72 | 42.77 |
| TF18 | 15131982900 | 15081376800 | 99.67 | 0.03 | 97.64 | 92.42 | 45.72 |
| TF26 | 17081151300 | 17050491300 | 99.82 | 0.03 | 97.73 | 93.45 | 42.02 |
| TF21 | 20585755500 | 19654384900 | 97.97 | 0.03 | 97.17 | 91.17 | 43.19 |
| TF16 | 15295750200 | 15165051300 | 99.15 | 0.03 | 97.8 | 93.02 | 48.74 |
| TF24 | 13436111100 | 13374461100 | 99.54 | 0.03 | 97.32 | 92.23 | 45.06 |
| TF27 | 18987837000 | 18948658500 | 99.79 | 0.03 | 97.69 | 92.56 | 42.74 |
| TF22 | 10900304600 | 9621680700 | 97.89 | 0.03 | 97.4 | 92.11 | 44.03 |
| TF19 | 15056422500 | 14981473200 | 99.5 | 0.03 | 97.15 | 91.89 | 44.84 |
| TF20 | 11243355300 | 10633694700 | 98.02 | 0.03 | 97.5 | 92.16 | 43.77 |
| TF28 | 16721715300 | 16691623200 | 99.82 | 0.03 | 97.69 | 92.44 | 42.28 |
| TF29 | 11620669200 | 11598804600 | 99.81 | 0.03 | 97.69 | 92.47 | 42.4 |
| TF17 | 14593041000 | 14171051400 | 97.96 | 0.03 | 97.53 | 92.09 | 42.84 |
| WN26 | 13152826949 | 9392341200 | 99.78 | 0.04 | 96.45 | 89.03 | 41.94 |
| WN27 | 11272957800 | 8947219200 | 99.81 | 0.04 | 96.53 | 89.38 | 43.06 |
| WN28 | 14375870100 | 14355565200 | 99.86 | 0.03 | 97.43 | 92.03 | 42.25 |
| WN29 | 11103065100 | 11086961400 | 99.85 | 0.03 | 97.49 | 92.04 | 42.16 |
| WN30 | 12905653200 | 12886916100 | 99.85 | 0.03 | 97.56 | 92.31 | 42.17 |
| WN1 | 15277051200 | 15252275400 | 99.84 | 0.03 | 96.76 | 90.4 | 42.21 |
| WN2 | 17396075100 | 17346147900 | 99.71 | 0.03 | 96.67 | 90.59 | 41.61 |
| WN3 | 21198262700 | 21004311300 | 99.78 | 0.03 | 97.08 | 90.71 | 42.41 |
| WN4 | 16266709400 | 15896689800 | 99.84 | 0.04 | 96.41 | 88.97 | 42.13 |
| WN5 | 28828568900 | 27381967800 | 99.76 | 0.04 | 96.78 | 90 | 42.07 |
| WN6 | 12943227300 | 12921570600 | 99.83 | 0.03 | 97.34 | 91.75 | 42.23 |
| WN7 | 10834311500 | 10898036100 | 99.85 | 0.04 | 96.3 | 88.7 | 42.06 |
| WN8 | 16244115000 | 16182526800 | 99.62 | 0.03 | 96.71 | 90.59 | 41.78 |
| WN9 | 17360141700 | 16901079600 | 99.77 | 0.03 | 97.24 | 91.35 | 41.84 |
| WN10 | 12967528000 | 12724359900 | 99.84 | 0.03 | 97.34 | 91.81 | 42.26 |
| WN11 | 10782293400 | 10753495200 | 99.73 | 0.03 | 96.25 | 89.31 | 42.97 |
| WN12 | 17189799800 | 16832925000 | 99.83 | 0.04 | 96.03 | 88 | 43.03 |
| WN13 | 19397354100 | 19343591400 | 99.72 | 0.03 | 97.38 | 92.37 | 42.06 |
| WN14 | 12867708900 | 12757035300 | 99.83 | 0.04 | 96.37 | 88.79 | 42.1 |
| WN15 | 19458902600 | 18814276200 | 99.8 | 0.04 | 96.64 | 89.52 | 41.3 |
| WN16 | 16226894900 | 16145137200 | 99.82 | 0.04 | 95.95 | 87.77 | 41.84 |
| WN17 | 14893972200 | 14868991200 | 99.83 | 0.03 | 97.7 | 92.87 | 42.27 |
| WN18 | 16903255800 | 16862568600 | 99.76 | 0.03 | 96.2 | 89.22 | 42.45 |
| WN19 | 15844876500 | 15458318700 | 99.82 | 0.03 | 97.69 | 92.81 | 42.15 |
| WN20 | 11700665700 | 11663961300 | 99.69 | 0.03 | 97.14 | 91.16 | 42.07 |
| WN21 | 15977934600 | 15940610700 | 99.77 | 0.03 | 96.6 | 90.22 | 41.75 |
| WN22 | 18916761449 | 18064483800 | 99.4 | 0.04 | 96.23 | 88.69 | 48.19 |
| WN23 | 13879600500 | 13855876500 | 99.83 | 0.03 | 97.44 | 92.11 | 42.22 |
| WN24 | 11326517800 | 11428043100 | 99.83 | 0.03 | 96.74 | 90.44 | 42.12 |
| WN25 | 11790504600 | 11769954000 | 99.83 | 0.04 | 96.65 | 89.51 | 41.47 |
| CS1 | 12363849000 | 12340250100 | 99.81 | 0.03 | 97.69 | 92.79 | 42.25 |
| CS3 | 10849921200 | 10831032300 | 99.83 | 0.03 | 97.75 | 92.85 | 42.2 |
| CS2 | 12641563200 | 12617957100 | 99.81 | 0.03 | 97.84 | 93.16 | 42.22 |
| CS30 | 12514316700 | 12497223900 | 99.86 | 0.03 | 97.35 | 91.77 | 42.23 |
| CS29 | 11597517300 | 11577018900 | 99.82 | 0.03 | 97.28 | 91.58 | 42.23 |
| CS28 | 11980119000 | 11962319100 | 99.85 | 0.03 | 97.6 | 92.53 | 42.3 |
| CS27 | 14154030100 | 13126763400 | 99.85 | 0.04 | 96.53 | 89.42 | 42.11 |
| CS26 | 12415905000 | 12397269300 | 99.85 | 0.03 | 97.34 | 91.61 | 42.07 |
| CS25 | 14305920300 | 14283192900 | 99.84 | 0.03 | 97.69 | 92.72 | 42.14 |
| CS24 | 13956606100 | 13004933100 | 99.83 | 0.03 | 97.55 | 92.27 | 42.2 |
| CS23 | 11293538700 | 11277696600 | 99.86 | 0.03 | 97.56 | 92.35 | 42.25 |
| CS22 | 10840940700 | 10821179400 | 99.82 | 0.03 | 97.6 | 92.53 | 42.39 |
| CS21 | 25156015100 | 24620232600 | 99.76 | 0.04 | 96.73 | 89.82 | 42.13 |
| CS20 | 13155836100 | 13127157600 | 99.78 | 0.03 | 97.47 | 92.28 | 42.36 |
| CS19 | 14317766700 | 14294750400 | 99.84 | 0.03 | 97.41 | 92.01 | 42.33 |
| CS18 | 14581353300 | 14557699800 | 99.84 | 0.03 | 97.86 | 93.31 | 42.33 |
| CS17 | 10986333800 | 10519227900 | 99.83 | 0.04 | 96.27 | 88.67 | 42.16 |
| CS16 | 13612533600 | 13585647000 | 99.8 | 0.03 | 97.73 | 93.06 | 42.28 |
| CS15 | 12303826500 | 12283220700 | 99.83 | 0.03 | 97.61 | 92.53 | 42.67 |
| CS14 | 14045764800 | 14022808800 | 99.84 | 0.03 | 97.66 | 92.69 | 42.48 |
| CS13 | 12209246100 | 12187591800 | 99.82 | 0.03 | 97.5 | 92.18 | 42.19 |
| CS12 | 11084416800 | 11064182100 | 99.82 | 0.03 | 97.47 | 92.16 | 42.3 |
| CS11 | 13100709300 | 13080309000 | 99.84 | 0.03 | 97.42 | 91.98 | 42.17 |
| CS10 | 11533720900 | 11442298500 | 98.12 | 0.04 | 96.28 | 88.92 | 42.28 |
| CS9 | 22741517600 | 22194277500 | 99.75 | 0.04 | 96.84 | 90.03 | 42.25 |
| CS8 | 12908367300 | 12883538400 | 99.81 | 0.03 | 97.27 | 91.56 | 42.32 |
| CS7 | 11583182200 | 11809010700 | 99.76 | 0.04 | 96.25 | 88.61 | 42.22 |
| CS6 | 13471779600 | 13440166800 | 99.77 | 0.03 | 97.56 | 92.53 | 43.88 |
| CS5 | 13462156800 | 13437436200 | 99.82 | 0.03 | 97.41 | 91.92 | 42.4 |
| CS4 | 11179647900 | 11155494600 | 99.78 | 0.03 | 97.75 | 93.08 | 42.25 |
| PD1 | 15611282700 | 15573119100 | 99.76 | 0.03 | 96.08 | 88.77 | 42.58 |
| PD2 | 13672056700 | 13424535900 | 99.81 | 0.04 | 96.22 | 88.37 | 43.02 |
| PD3 | 22677966800 | 22355806800 | 99.78 | 0.04 | 96.69 | 89.66 | 42.95 |
| PD4 | 13392267600 | 13357635300 | 99.74 | 0.03 | 97.38 | 91.86 | 45.15 |
| PD5 | 15720921900 | 15679016400 | 99.73 | 0.03 | 96.77 | 90.69 | 42.55 |
| PD6 | 20813772800 | 19184277300 | 99.82 | 0.03 | 97.16 | 91.07 | 43.35 |
| PD7 | 16849821700 | 16727826300 | 99.84 | 0.03 | 97.5 | 92.2 | 42.78 |
| PD8 | 13167833300 | 12548990700 | 99.77 | 0.04 | 96.43 | 88.9 | 42.98 |
| PD9 | 16383803100 | 15951988500 | 99.32 | 0.03 | 96.88 | 90.55 | 48.48 |
| PD10 | 11326380900 | 11308702500 | 99.84 | 0.03 | 96.96 | 90.69 | 42.57 |
| PD11 | 11059307400 | 11040760200 | 99.83 | 0.03 | 97.2 | 91.29 | 42.79 |
| PD12 | 14447470200 | 14428958700 | 99.87 | 0.03 | 97.3 | 91.48 | 42.05 |
| PD13 | 12050005200 | 11733714300 | 99.84 | 0.03 | 97.31 | 91.56 | 42.98 |
| PD14 | 13696637400 | 13675383000 | 99.84 | 0.03 | 97.23 | 91.37 | 43.15 |
| PD15 | 12192379200 | 12139298400 | 99.56 | 0.03 | 97.3 | 91.59 | 43.88 |
| PD16 | 14299952100 | 14268118500 | 99.78 | 0.03 | 97.27 | 91.53 | 43.31 |
| PD17 | 14400567000 | 14373018000 | 99.81 | 0.03 | 97.36 | 91.84 | 43.12 |
| PD18 | 11862114000 | 11842223100 | 99.83 | 0.03 | 97.13 | 91.12 | 43.06 |
| PD19 | 15561487700 | 15027020100 | 99.63 | 0.04 | 95.62 | 86.71 | 42.72 |
| PD20 | 20720073300 | 20308498324 | 99.84 | 0.04 | 97.62 | 82.71 | 41.72 |
| PD21 | 11614538400 | 11594071200 | 99.82 | 0.03 | 97.38 | 91.85 | 43.03 |
| PD22 | 10821017700 | 10804544400 | 99.85 | 0.03 | 97.21 | 91.14 | 42.86 |
| PD23 | 11424689700 | 11408898900 | 99.86 | 0.03 | 97.23 | 91.31 | 41.58 |
| PD24 | 12350235300 | 12328361400 | 99.82 | 0.03 | 97.45 | 92.03 | 42.75 |
| PD25 | 10753541100 | 10730123400 | 99.78 | 0.03 | 97.47 | 92.7 | 42.15 |
| PD26 | 11516838400 | 10120599300 | 99.4 | 0.04 | 96.23 | 88.69 | 48.19 |
| PD27 | 11476865249 | 11422850100 | 99.73 | 0.03 | 97.12 | 91.05 | 43.31 |
| PD28 | 11504347000 | 9989949900 | 99.63 | 0.03 | 97.28 | 91.2 | 44.9 |
| PD29 | 13890671400 | 13707210300 | 98.68 | 0.03 | 97.06 | 90.86 | 44.03 |
| PD30 | 18223163700 | 18174408000 | 99.73 | 0.03 | 96 | 88.55 | 42.77 |
| WM1 | 11595093300 | 11572887900 | 99.81 | 0.03 | 97.47 | 92.21 | 43.51 |
| WM2 | 13050934000 | 12728520900 | 99.84 | 0.03 | 97.44 | 92.06 | 41.51 |
| WM3 | 13413372900 | 13390983600 | 99.83 | 0.03 | 97.42 | 91.98 | 42.23 |
| WM4 | 13462474500 | 13439746500 | 99.83 | 0.03 | 97.22 | 91.34 | 42.13 |
| WM5 | 14339194500 | 14310094500 | 99.8 | 0.03 | 97.59 | 92.52 | 42.11 |
| WM6 | 12044781000 | 12027658500 | 99.86 | 0.03 | 97 | 90.68 | 41.95 |
| WM7 | 13858635300 | 13837288500 | 99.85 | 0.03 | 96.97 | 90.67 | 42.03 |
| WM8 | 16236632700 | 16158409800 | 99.52 | 0.03 | 96.73 | 90.75 | 41.8 |
| WM9 | 10894617900 | 10833034800 | 99.43 | 0.03 | 97.48 | 92.19 | 41.94 |
| WM10 | 13934382000 | 13895628600 | 99.72 | 0.03 | 96.09 | 88.91 | 41.88 |
| WM11 | 12593974200 | 12405243900 | 98.5 | 0.03 | 97.31 | 91.61 | 43.66 |
| WM12 | 15482012100 | 15445703400 | 99.77 | 0.04 | 95.72 | 88.18 | 41.74 |
| WM13 | 12605077800 | 12581769900 | 99.82 | 0.03 | 97.62 | 92.64 | 42.84 |
| WM14 | 11620004400 | 11597584200 | 99.81 | 0.03 | 97.59 | 92.56 | 41.73 |
| WM15 | 11449358100 | 11431077000 | 99.84 | 0.03 | 97.34 | 91.82 | 41.64 |
| WM16 | 20210070500 | 19580629500 | 99.8 | 0.04 | 96.97 | 90.48 | 41.15 |
| WM17 | 14215493400 | 14192570400 | 99.84 | 0.03 | 97.45 | 92.14 | 41.36 |
| WM18 | 12151560600 | 12131086800 | 99.83 | 0.03 | 97.41 | 92.06 | 41.26 |
| WM19 | 15613449300 | 15579586200 | 99.78 | 0.03 | 96.31 | 89.51 | 42.07 |
| WM20 | 10754807649 | 9974217600 | 99.82 | 0.03 | 97.46 | 92.19 | 42.06 |
| WM21 | 11812042800 | 11793574800 | 99.84 | 0.03 | 97.49 | 92.24 | 41.25 |
| WM22 | 17093024300 | 16293442500 | 99.77 | 0.03 | 97.12 | 91.02 | 41.55 |
| WM23 | 16839433400 | 16313115300 | 99.81 | 0.03 | 97.41 | 92.05 | 41.77 |
| WM24 | 20455526000 | 19862072100 | 99.78 | 0.04 | 96.45 | 89.03 | 41.94 |
| WM25 | 12639459000 | 12619683600 | 99.84 | 0.03 | 97.44 | 92.03 | 41.81 |
| WM26 | 14165635700 | 13453050300 | 99.86 | 0.04 | 96.5 | 89.12 | 40.42 |
| WM27 | 12789229200 | 12770619000 | 99.85 | 0.03 | 97.72 | 92.8 | 41.48 |
| WM28 | 12770649600 | 12750005400 | 99.84 | 0.03 | 97.7 | 92.8 | 41.41 |
| WM29 | 14163678900 | 14129947800 | 99.76 | 0.04 | 96.02 | 88.14 | 41.23 |
| WM30 | 12017913000 | 12000324000 | 99.85 | 0.03 | 97.27 | 91.5 | 41.54 |
| WM31 | 13160275200 | 13138744500 | 99.84 | 0.03 | 97.62 | 92.64 | 41.34 |
| CSWG1 | 11811692700 | 11791523400 | 99.83 | 0.03 | 97.94 | 93.59 | 41.55 |
| CSWG2 | 10984447500 | 10963876500 | 99.81 | 0.03 | 97.42 | 92.15 | 41.36 |
| CSWG3 | 12832525500 | 12806907300 | 99.8 | 0.03 | 97.17 | 91.44 | 41.27 |
| CSWG4 | 19145425100 | 18187039800 | 99.74 | 0.03 | 97.43 | 91.61 | 43.3 |
| CSWG5 | 15828097000 | 14967858000 | 99.74 | 0.04 | 94.67 | 84.25 | 40.89 |
| CSWG6 | 13426327200 | 13400171100 | 99.81 | 0.03 | 97.72 | 92.96 | 42.39 |
| CSWG7 | 12641894400 | 12620863200 | 99.83 | 0.03 | 97.41 | 92.03 | 42.28 |
| CSWG8 | 12440141100 | 12415150500 | 99.8 | 0.03 | 97.4 | 92.11 | 41.86 |
| CSWG9 | 12445767300 | 12421846500 | 99.81 | 0.03 | 97.78 | 93.1 | 41.72 |
| CSWG10 | 13366404600 | 13338351600 | 99.79 | 0.03 | 97.47 | 92.31 | 41.95 |
| CSWG11 | 14163450600 | 14132205300 | 99.78 | 0.03 | 98.03 | 93.84 | 41.39 |
| CSWG12 | 14489728200 | 14462671500 | 99.81 | 0.03 | 97.43 | 92.12 | 41.59 |
| CSWG13 | 12652514700 | 12633182100 | 99.85 | 0.03 | 97.5 | 92.12 | 41.18 |
| CSWG14 | 11181610500 | 11163759900 | 99.84 | 0.03 | 96.81 | 90.47 | 41.62 |
| CSWG15 | 16877847300 | 16851202800 | 99.84 | 0.03 | 96.97 | 91.06 | 41.43 |
| CSWG16 | 17442280200 | 17412458400 | 99.83 | 0.03 | 97.22 | 91.74 | 41.3 |
| CSWG17 | 16783613400 | 16747836000 | 99.79 | 0.03 | 97.17 | 91.64 | 42 |
| CSWG18 | 15210216600 | 15179576400 | 99.8 | 0.03 | 97.17 | 91.62 | 42.16 |
| CSWG19 | 17286471300 | 17248488900 | 99.78 | 0.03 | 97.37 | 92.15 | 42.84 |
| CSWG20 | 16509984000 | 16437852600 | 99.56 | 0.03 | 95.84 | 89.29 | 42.69 |
| CSWG21 | 17656873200 | 17618100300 | 99.78 | 0.03 | 96.77 | 90.66 | 41.73 |
| CSWG22 | 15098190300 | 15067932600 | 99.8 | 0.03 | 96.16 | 88.85 | 41.38 |
| CSWG23 | 15207429300 | 15177854700 | 99.81 | 0.03 | 96.55 | 90.07 | 41.5 |
| CSWG24 | 15695699700 | 15661005300 | 99.78 | 0.03 | 96.1 | 88.93 | 41.79 |
| CSWG25 | 13758275400 | 13737953700 | 99.85 | 0.04 | 96.19 | 88.69 | 41.11 |
| CSWG26 | 15725009700 | 15695081100 | 99.81 | 0.03 | 96.38 | 89.55 | 41.14 |
| CSWG27 | 15036107100 | 15004343100 | 99.79 | 0.03 | 96.36 | 89.68 | 41.38 |
| CSWG28 | 15790695600 | 15755961600 | 99.78 | 0.03 | 95.89 | 88.56 | 41.24 |
| CSWG29 | 13454353800 | 13429977600 | 99.82 | 0.03 | 96.35 | 89.5 | 41.06 |
| CSWG30 | 17054046600 | 17011554000 | 99.75 | 0.03 | 96.29 | 89.56 | 41.88 |
| QD1 | 14980526100 | 14946076800 | 99.77 | 0.03 | 96.25 | 89.31 | 42.02 |
| QD2 | 11959296300 | 11938383900 | 99.83 | 0.03 | 97.17 | 91.26 | 42.48 |
| QD4 | 11346232800 | 11328570900 | 99.84 | 0.03 | 97.38 | 91.78 | 42.33 |
| QD3 | 12060536400 | 12040395600 | 99.83 | 0.03 | 97.35 | 91.59 | 42.09 |
| QD5 | 12977313600 | 12540319800 | 99.83 | 0.03 | 97.5 | 92.17 | 42.25 |
| QD6 | 14068276900 | 13911044100 | 99.81 | 0.03 | 97.4 | 91.97 | 42.53 |
| QD7 | 13567794900 | 13539031200 | 99.79 | 0.03 | 97.8 | 93.14 | 42.43 |
| QD8 | 13536852300 | 13512882000 | 99.82 | 0.03 | 97.5 | 92.16 | 42.32 |
| QD9 | 12545579700 | 12524490600 | 99.83 | 0.03 | 97.51 | 92.29 | 42.38 |
| QD10 | 16232651449 | 16088443800 | 99.85 | 0.03 | 97.6 | 92.44 | 42.27 |
| QD11 | 11254429070 | 11681564100 | 99.84 | 0.04 | 96.32 | 88.67 | 42.16 |
| QD12 | 12278867100 | 12257286900 | 99.82 | 0.03 | 97.74 | 92.89 | 42.2 |
| QD13 | 19486766949 | 19286088900 | 99.84 | 0.04 | 96.32 | 88.67 | 42.16 |
| QD14 | 11463529800 | 11440434600 | 99.8 | 0.03 | 97.58 | 92.39 | 42.36 |
| QD15 | 13601597400 | 13577753400 | 99.82 | 0.03 | 97.45 | 92.02 | 42.26 |
| QD16 | 23095628200 | 22968313800 | 99.73 | 0.03 | 97.12 | 91.05 | 43.31 |
| QD17 | 12141888600 | 12123716700 | 99.85 | 0.03 | 97.5 | 92.14 | 42.35 |
| QD18 | 15003878400 | 14962694400 | 99.73 | 0.03 | 96.56 | 90.19 | 42.06 |
| QD19 | 10915654800 | 10888801800 | 99.75 | 0.03 | 96.53 | 89.78 | 42.06 |
| QD20 | 13995998700 | 13744878900 | 99.82 | 0.04 | 95.65 | 86.86 | 42.04 |
| QD21 | 14044411800 | 14014195200 | 99.78 | 0.03 | 97.52 | 92.29 | 42.36 |
| QD22 | 13343430900 | 13318490100 | 99.81 | 0.03 | 97.59 | 92.5 | 42.27 |
| QD23 | 13380960000 | 13358281500 | 99.83 | 0.03 | 97.53 | 92.32 | 42.27 |
| QD24 | 11786259000 | 11767200600 | 99.84 | 0.03 | 97.51 | 92.12 | 42.14 |
| QD25 | 11609439000 | 11591185800 | 99.84 | 0.03 | 97.59 | 92.41 | 42.2 |
| QD26 | 12893450000 | 12019251600 | 99.77 | 0.03 | 97.13 | 91.08 | 42.34 |
| QD27 | 12638282400 | 11835674100 | 99.77 | 0.03 | 97.06 | 90.81 | 42.31 |
| QD28 | 11151325200 | 11132224200 | 99.83 | 0.03 | 97.14 | 91.1 | 42.03 |
| QD29 | 21628380700 | 21545193000 | 99.75 | 0.04 | 96.8 | 90.11 | 43.3 |
| QD30 | 11094334500 | 11071244100 | 99.79 | 0.03 | 97.62 | 92.6 | 42.42 |
| YS1 | 14932315500 | 14901002400 | 99.79 | 0.04 | 95.62 | 87.74 | 41.87 |
| YS2 | 15022535400 | 14981166900 | 99.72 | 0.03 | 96.47 | 90 | 43.36 |
| YS3 | 13429588500 | 13400126700 | 99.78 | 0.03 | 96.56 | 90.19 | 42.06 |
| YS4 | 16019059500 | 15982842300 | 99.77 | 0.03 | 96.59 | 90.23 | 41.85 |
| YS5 | 15192823500 | 15163067100 | 99.8 | 0.03 | 96.52 | 90.06 | 41.22 |
| YS6 | 15481188000 | 15452294700 | 99.81 | 0.03 | 96.53 | 89.81 | 41.12 |
| YS7 | 11908302600 | 11885988900 | 99.81 | 0.03 | 96.44 | 89.73 | 41.42 |
| YS8 | 19556509200 | 19523409600 | 99.83 | 0.03 | 97.04 | 91.22 | 41.12 |
| YS9 | 12958067700 | 12939177300 | 99.85 | 0.03 | 96.93 | 90.81 | 41.63 |
| YS10 | 16309192800 | 16260136500 | 99.7 | 0.03 | 96.03 | 88.62 | 41.69 |
| YS11 | 16892454600 | 16841194800 | 99.7 | 0.03 | 96.58 | 90.3 | 42.37 |
| YS12 | 14175695100 | 14130223500 | 99.68 | 0.03 | 96.73 | 90.51 | 42.03 |
| YS13 | 20897450300 | 20738255400 | 99.82 | 0.03 | 97.46 | 92.19 | 42.06 |
| YS16 | 19344112749 | 18709203300 | 99.63 | 0.03 | 97.28 | 91.2 | 44.9 |
| YS14 | 15743352300 | 15720687300 | 99.86 | 0.03 | 97.06 | 91.17 | 41.35 |
| YS15 | 15707211000 | 15681838200 | 99.84 | 0.03 | 96.38 | 89.48 | 41.19 |
| YS17 | 22102913619 | 21242585100 | 99.86 | 0.04 | 96.5 | 89.12 | 40.42 |
| YS18 | 16042979400 | 16009688700 | 99.79 | 0.03 | 96.81 | 90.48 | 43.11 |
| YS19 | 14039732700 | 13995092400 | 99.68 | 0.03 | 96.53 | 90.11 | 42.58 |
| YS20 | 15630211200 | 15595820100 | 99.78 | 0.03 | 96.6 | 90.13 | 42.23 |
| YS21 | 14279314800 | 14248886700 | 99.79 | 0.03 | 96.31 | 89.41 | 42.55 |
| YS22 | 14583777000 | 14549537700 | 99.77 | 0.03 | 95.72 | 87.83 | 43.25 |
| YS23 | 14286970200 | 14252027400 | 99.76 | 0.03 | 96.31 | 89.41 | 43.38 |
| YS24 | 14618943300 | 14580472200 | 99.74 | 0.03 | 96.14 | 89 | 42.05 |
| YS25 | 14781410100 | 14749059900 | 99.78 | 0.03 | 96.34 | 89.49 | 42.16 |
| YS26 | 11384166900 | 10097842500 | 99.74 | 0.04 | 96.77 | 89.87 | 43.59 |
| YS27 | 16906323100 | 16618291800 | 99.83 | 0.03 | 97.25 | 91.46 | 43.19 |
| YS28 | 13295545500 | 13276964400 | 99.86 | 0.03 | 97.31 | 91.68 | 41.72 |
| YS29 | 11751225900 | 11731386600 | 99.83 | 0.03 | 97.35 | 91.78 | 42.8 |
| XY22 | 17059776300 | 17020033500 | 99.77 | 0.03 | 96.44 | 89.75 | 42.28 |
| XY21 | 11983104300 | 11818273200 | 98.62 | 0.03 | 96.9 | 90.2 | 43.59 |
| XY23 | 18051434100 | 18002091300 | 99.73 | 0.03 | 96.73 | 90.64 | 42.38 |
| XY20 | 11516642100 | 11496088800 | 99.82 | 0.03 | 97.52 | 92.23 | 42.56 |
| XY19 | 12518199000 | 12494818200 | 99.81 | 0.03 | 97.67 | 92.72 | 42.28 |
| XY18 | 12443783100 | 12423581700 | 99.84 | 0.03 | 97.74 | 92.91 | 42.42 |
| XY17 | 10741934549 | 9772297400 | 99.81 | 0.03 | 97.4 | 91.97 | 42.53 |
| XY16 | 10745934849 | 11717118000 | 99.83 | 0.03 | 97.5 | 92.17 | 42.25 |
| XY15 | 14351289000 | 14318167500 | 99.77 | 0.03 | 96.71 | 90.47 | 42.27 |
| XY30 | 14126534900 | 13358364300 | 99.83 | 0.03 | 97.11 | 90.88 | 41.86 |
| XY29 | 11400238500 | 11383117800 | 99.85 | 0.03 | 97.35 | 91.78 | 42.33 |
| XY28 | 14649013800 | 14622079500 | 99.82 | 0.03 | 97.66 | 92.67 | 42.43 |
| XY27 | 13940434800 | 13907425200 | 99.76 | 0.04 | 96.17 | 88.68 | 41.69 |
| XY26 | 11427884700 | 11408297400 | 99.83 | 0.03 | 97.46 | 92.03 | 42.48 |
| XY25 | 11687512200 | 11670775800 | 99.86 | 0.03 | 97.48 | 92.11 | 42.6 |
| XY24 | 15453924000 | 15412856100 | 99.73 | 0.03 | 96.44 | 89.84 | 42.64 |
| XY14 | 15898888500 | 15864615300 | 99.78 | 0.03 | 97.28 | 91.89 | 42.16 |
| XY13 | 13198797000 | 13178156700 | 99.84 | 0.03 | 96.93 | 90.97 | 42.36 |
| XY12 | 18955642600 | 18655435500 | 99.85 | 0.03 | 97.6 | 92.44 | 42.27 |
| XY11 | 14006769600 | 13988111400 | 99.87 | 0.03 | 97.39 | 91.77 | 42.13 |
| XY10 | 10995363600 | 10979790300 | 99.86 | 0.03 | 97.85 | 93.19 | 42.18 |
| XY9 | 11688001200 | 11670464400 | 99.85 | 0.03 | 97.53 | 92.28 | 42.25 |
| XY8 | 15804127149 | 15491489700 | 98.86 | 0.03 | 97.3 | 91.5 | 43.46 |
| XY7 | 11822228700 | 11796225900 | 99.78 | 0.03 | 97.53 | 92.39 | 42.48 |
| XY6 | 12444775800 | 12421247400 | 99.81 | 0.03 | 97.45 | 92.07 | 42.36 |
| XY5 | 13877894400 | 13847042100 | 99.78 | 0.03 | 97.91 | 93.41 | 42.25 |
| XY4 | 12440181000 | 12407902500 | 99.74 | 0.03 | 97.75 | 93 | 42.88 |
| XY3 | 12402879000 | 12380651400 | 99.82 | 0.03 | 97.9 | 93.41 | 42.4 |
| XY2 | 13065786600 | 13037707800 | 99.79 | 0.03 | 97.64 | 92.59 | 42.45 |
| XY1 | 19050968100 | 18777727800 | 99.73 | 0.03 | 97.31 | 91.53 | 42.16 |

1) Effective Rate (%) was calculated as Clean Base/Raw Base. 2) Q20、Q30 (%): The proportion of bases with Phred values greater than 20 and 30 in the total base. 3) GC content (%): The proportion of GC in the total base number in the sequencing data.

Table S2 The mapping results of genome-wide sequencing data

| Sample | Clean reads | Mapped reads | Mapping rate(%) | Coverage 1X(%) | Coverage 4X(%) | Coverage 10X(%) | Coverage 20X(%) | Average depth |
| --- | --- | --- | --- | --- | --- | --- | --- | --- |
| SHXY19 | 87010810 | 86385938 | 99.28 | 96.21 | 93.77 | 65.05 | 5.53 | 12.18 |
| SHXY24 | 84495420 | 83792019 | 99.17 | 96.19 | 92.75 | 59.01 | 7.06 | 11.87 |
| SHXY25 | 89016974 | 87970731 | 98.82 | 96.37 | 92.1 | 51.45 | 5.42 | 11.02 |
| SHXY16 | 87477896 | 86915244 | 99.36 | 96.66 | 93.56 | 68.55 | 4.78 | 12.25 |
| SHXY3 | 81234936 | 80290723 | 98.84 | 96.65 | 89.28 | 41.77 | 2.55 | 9.58 |
| SHXY11 | 93495210 | 92131624 | 98.54 | 96.51 | 87.37 | 50.31 | 15.07 | 12.62 |
| SHXY30 | 109187916 | 108184657 | 99.08 | 96.35 | 94.45 | 76.27 | 17.66 | 14.83 |
| SHXY17 | 81850364 | 80861928 | 98.79 | 96.27 | 89.49 | 45.15 | 6.26 | 10.55 |
| SHXY7 | 75530460 | 74869439 | 99.12 | 96.54 | 90.26 | 49.78 | 4.45 | 10.55 |
| SHXY20 | 79628160 | 78855623 | 99.03 | 96.12 | 90.74 | 51.89 | 6.77 | 11.16 |
| SHXY5 | 87940882 | 87164795 | 99.12 | 96.25 | 93.12 | 63.45 | 7.9 | 12.31 |
| SHXY29 | 91467166 | 90410051 | 98.96 | 96.79 | 90.73 | 46.61 | 2.75 | 11.71 |
| SHXY12 | 114320646 | 113574868 | 99.35 | 96.86 | 95.03 | 86.29 | 19.45 | 15.86 |
| SHXY6 | 89011686 | 88435509 | 99.35 | 96.57 | 93.53 | 70.45 | 5.51 | 12.51 |
| SHXY18 | 83433956 | 82560185 | 98.95 | 96.18 | 90.91 | 50.31 | 7 | 11.12 |
| SHXY22 | 76910866 | 76306781 | 99.21 | 96.13 | 92.04 | 52.93 | 3.71 | 10.84 |
| SHXY2 | 63021632 | 62348825 | 98.93 | 96.36 | 84.75 | 27.63 | 1.02 | 7.98 |
| SHXY1 | 98744244 | 97568474 | 98.81 | 96.98 | 93.53 | 65 | 9.32 | 12.65 |
| SHXY10 | 91674428 | 90986139 | 99.25 | 96.74 | 93.44 | 67.88 | 8.91 | 12.79 |
| SHXY8 | 72266675 | 71215335 | 98.55 | 95.89 | 80.59 | 34.72 | 7.05 | 9.52 |
| SHXY4 | 75932922 | 75058714 | 98.85 | 96.53 | 88.08 | 37.7 | 1.78 | 9.07 |
| SHXY23 | 72960878 | 72271670 | 99.06 | 96.04 | 89.27 | 45.64 | 4.63 | 10.28 |
| SHXY26 | 80433882 | 79882483 | 99.31 | 96.63 | 92.97 | 61.85 | 2.78 | 11.33 |
| SHXY15 | 77270884 | 76377566 | 98.84 | 94.26 | 64.71 | 7.93 | 0.48 | 5.41 |
| SHXY9 | 100983746 | 99822384 | 98.85 | 96.86 | 92.32 | 63.94 | 17.81 | 13.92 |
| SHXY13 | 97544448 | 96780950 | 99.22 | 96.77 | 94.08 | 73.91 | 10.16 | 13.46 |
| SHXY27 | 88346596 | 87342463 | 98.86 | 96.37 | 92.75 | 53.62 | 4.21 | 11 |
| SHXY21 | 113865142 | 113158559 | 99.38 | 96.23 | 94.76 | 86.48 | 19.66 | 16 |
| SHXY28 | 104103448 | 103318527 | 99.25 | 96.38 | 94.88 | 81.67 | 10.28 | 14.37 |
| SHXY14 | 76916090 | 76176332 | 99.04 | 96.4 | 84.75 | 26.44 | 0.96 | 7.85 |
| TF11 | 98166384 | 97461440 | 99.28 | 96.73 | 94.24 | 75.39 | 8.72 | 13.53 |
| TF4 | 80332804 | 79248873 | 98.65 | 96.35 | 86.4 | 44.95 | 9.4 | 10.88 |
| TF15 | 88360724 | 87515189 | 99.04 | 96.98 | 93.84 | 61.16 | 4.55 | 11.58 |
| TF8 | 78373980 | 77685571 | 99.12 | 96.83 | 92.82 | 54.35 | 2.6 | 10.66 |
| TF9 | 64974088 | 64364129 | 99.06 | 96.55 | 89.01 | 39.6 | 1.74 | 9.19 |
| TF14 | 103178214 | 101953916 | 98.81 | 96.77 | 91.3 | 63.14 | 20.25 | 14.21 |
| TF6 | 72573924 | 71849725 | 99.00 | 96.26 | 84.33 | 25.47 | 1 | 7.77 |
| TF10 | 108115288 | 107176338 | 99.13 | 96.84 | 94.71 | 79.96 | 14.52 | 14.78 |
| TF7 | 64984268 | 64466209 | 99.20 | 96.59 | 90.61 | 37.61 | 0.99 | 8.97 |
| TF12 | 119138560 | 118136453 | 99.16 | 96.9 | 95.19 | 84.65 | 22.7 | 16.3 |
| TF3 | 64127064 | 63614176 | 99.20 | 96.58 | 90.01 | 36.46 | 0.91 | 8.8 |
| TF5 | 82413472 | 81715071 | 99.15 | 96.58 | 92.88 | 61.88 | 2.96 | 11.38 |
| TF13 | 102587278 | 101693520 | 99.13 | 96.84 | 94.61 | 78.02 | 10.81 | 14.12 |
| TF2 | 73453060 | 72676546 | 98.94 | 96.81 | 90.38 | 43.18 | 2.44 | 9.67 |
| TF1 | 122372580 | 121287226 | 99.11 | 96.91 | 95.17 | 84.46 | 24.7 | 16.59 |
| TF23 | 110753652 | 109347160 | 98.73 | 96.54 | 94.28 | 69.35 | 14.84 | 14.01 |
| TF30 | 138691750 | 137222011 | 98.94 | 96.59 | 95.41 | 86.28 | 28.52 | 17.25 |
| TF25 | 112326478 | 111375069 | 99.15 | 97.03 | 95.29 | 82.62 | 20.02 | 15.56 |
| TF18 | 100542512 | 99450342 | 98.91 | 96.34 | 92.7 | 65.07 | 17.19 | 13.92 |
| TF26 | 113669942 | 112781692 | 99.22 | 96.26 | 94.86 | 85.62 | 16.51 | 15.69 |
| TF21 | 144362566 | 141704206 | 99.12 | 97.52 | 96.77 | 95.37 | 88.52 | 36.65 |
| TF16 | 101100342 | 99390553 | 98.31 | 96.02 | 86.38 | 50.44 | 18.03 | 13.64 |
| TF24 | 89163074 | 87992099 | 98.79 | 96.21 | 89.81 | 40.89 | 3 | 9.71 |
| TF27 | 126324390 | 125304779 | 99.27 | 96.21 | 93.96 | 64.39 | 3.14 | 11.73 |
| TF22 | 64144538 | 63451183 | 98.92 | 96.11 | 87.6 | 29.34 | 1.09 | 8.28 |
| TF19 | 99876488 | 98588535 | 98.86 | 96.16 | 89.67 | 40.95 | 2.63 | 9.57 |
| TF20 | 70891298 | 70237642 | 99.08 | 96.67 | 90.23 | 44.7 | 2.22 | 9.77 |
| TF28 | 111277488 | 110544549 | 99.34 | 96.27 | 94.82 | 85.16 | 15.65 | 15.42 |
| TF29 | 77325364 | 76841019 | 99.37 | 96.05 | 93.22 | 56.6 | 1.79 | 10.77 |
| TF17 | 94473676 | 93735059 | 99.22 | 96.98 | 94.26 | 71.88 | 8.27 | 13.08 |
| WN26 | 62615608 | 62129574 | 99.22 | 96.37 | 89.34 | 37.59 | 0.76 | 8.9 |
| WN27 | 59648128 | 59196156 | 99.24 | 95.78 | 89.45 | 30.27 | 0.61 | 8.33 |
| WN28 | 95703768 | 95025569 | 99.29 | 96.16 | 94.32 | 76.95 | 7.34 | 13.41 |
| WN29 | 73913076 | 73362206 | 99.25 | 96.46 | 91.68 | 53 | 1.66 | 10.33 |
| WN30 | 85912774 | 85364785 | 99.36 | 96.06 | 93.83 | 68.48 | 3.72 | 12.08 |
| WN1 | 101681836 | 100840642 | 99.17 | 96.3 | 94.7 | 81.38 | 9.75 | 14.2 |
| WN2 | 115640986 | 114669559 | 99.16 | 96.31 | 94.78 | 86.43 | 18.74 | 15.89 |
| WN3 | 140028742 | 138959883 | 99.24 | 97.26 | 95.94 | 89.65 | 46.2 | 19.33 |
| WN4 | 105977932 | 105003373 | 99.08 | 97.18 | 95.33 | 80.89 | 13.51 | 14.59 |
| WN5 | 182546452 | 181412487 | 99.38 | 97.22 | 96.12 | 93.79 | 77.36 | 25.36 |
| WN6 | 86143804 | 85525976 | 99.28 | 95.94 | 93.76 | 68.67 | 3.62 | 12.11 |
| WN7 | 72653574 | 72103859 | 99.24 | 96.01 | 92.69 | 49.37 | 1.21 | 10.07 |
| WN8 | 107883512 | 106856809 | 99.05 | 96.9 | 94.8 | 80.55 | 14.01 | 14.7 |
| WN9 | 112673864 | 112002119 | 99.40 | 96.36 | 94.83 | 86.64 | 18.74 | 15.86 |
| WN10 | 84829066 | 84223368 | 99.29 | 96.26 | 94.03 | 67.51 | 3.26 | 11.95 |
| WN11 | 71689968 | 70902016 | 98.90 | 96.82 | 91.65 | 46.82 | 1.33 | 9.79 |
| WN12 | 112219500 | 111406350 | 99.28 | 96.89 | 95.02 | 86.22 | 18.51 | 15.71 |
| WN13 | 128957276 | 127899123 | 99.18 | 97.13 | 95.66 | 87.49 | 31.88 | 17.45 |
| WN14 | 85046902 | 84459398 | 99.31 | 96.8 | 93.45 | 66.75 | 4.07 | 11.93 |
| WN15 | 125428508 | 124161322 | 98.90 | 97.13 | 94.41 | 71.28 | 7.74 | 17.15 |
| WN16 | 107634248 | 106458433 | 98.91 | 97.26 | 95.4 | 79.08 | 15.61 | 14.69 |
| WN17 | 99126608 | 98429212 | 99.30 | 96.23 | 94.54 | 79.5 | 8.66 | 13.88 |
| WN18 | 112417124 | 111290018 | 99.00 | 97.13 | 95.3 | 82.57 | 16.72 | 15.21 |
| WN19 | 103055458 | 102375654 | 99.34 | 96.92 | 94.88 | 82.05 | 11.66 | 14.43 |
| WN20 | 77759742 | 77238561 | 99.33 | 96.04 | 93.2 | 58.52 | 1.98 | 10.95 |
| WN21 | 106270738 | 105376863 | 99.16 | 96.97 | 94.82 | 79.67 | 13.67 | 14.47 |
| WN22 | 120429892 | 119127488 | 98.92 | 97.04 | 95.19 | 82.11 | 18.82 | 15.43 |
| WN23 | 92372510 | 91690394 | 99.26 | 96.74 | 93.93 | 72.49 | 6.44 | 12.87 |
| WN24 | 76186954 | 75445401 | 99.03 | 96.96 | 93.4 | 54.17 | 1.81 | 10.54 |
| WN25 | 78466360 | 77980548 | 99.38 | 95.9 | 92.8 | 60.37 | 2.26 | 11.1 |
| CS1 | 82268334 | 81627550 | 99.22 | 96.59 | 92.99 | 63.21 | 3.21 | 11.5 |
| CS30 | 83314826 | 82727936 | 99.30 | 96.65 | 93.15 | 64.52 | 3.49 | 11.65 |
| CS29 | 77180126 | 76661001 | 99.33 | 95.96 | 93.09 | 58.21 | 1.99 | 10.88 |
| CS28 | 79748794 | 79196478 | 99.31 | 95.99 | 93.4 | 61.45 | 2.37 | 11.24 |
| CS27 | 87511756 | 86871533 | 99.27 | 96.83 | 94.16 | 68.91 | 4.37 | 12.18 |
| CS26 | 82648462 | 82042335 | 99.27 | 96.53 | 92.91 | 63.55 | 3.38 | 11.53 |
| CS25 | 95221286 | 94609391 | 99.36 | 96.71 | 94.41 | 76.61 | 7.16 | 13.28 |
| CS24 | 86699554 | 86083044 | 99.29 | 96.71 | 93.43 | 68.17 | 4.56 | 12.14 |
| CS23 | 75184644 | 74659934 | 99.30 | 96.57 | 92.79 | 54.79 | 1.71 | 10.5 |
| CS22 | 72141196 | 71600637 | 99.25 | 96.5 | 91.48 | 50.12 | 1.49 | 10.08 |
| CS20 | 87514384 | 86910529 | 99.31 | 96.06 | 93.85 | 69.8 | 4.54 | 12.31 |
| CS19 | 95298336 | 94643767 | 99.31 | 96.06 | 94.2 | 76.61 | 7.36 | 13.39 |
| CS18 | 97051332 | 96346061 | 99.27 | 96.68 | 94.13 | 75.38 | 8.92 | 13.48 |
| CS21 | 164134884 | 163178565 | 99.42 | 96.54 | 95.42 | 93.18 | 68.21 | 23.01 |
| CS17 | 70128186 | 69603542 | 99.25 | 96.71 | 92.14 | 48.27 | 1.27 | 9.89 |
| CS16 | 90570980 | 89962059 | 99.33 | 96.13 | 94.1 | 72.32 | 5.3 | 12.69 |
| CS15 | 81888138 | 81164295 | 99.21 | 96.17 | 87.32 | 30.57 | 0.66 | 11.38 |
| CS14 | 93485392 | 92807201 | 99.27 | 96.69 | 93.94 | 73.02 | 7.35 | 13.04 |
| CS13 | 81250612 | 80644500 | 99.25 | 96.59 | 92.9 | 62.53 | 2.94 | 11.4 |
| CS12 | 73761214 | 73244846 | 99.30 | 95.93 | 92.68 | 53.04 | 1.56 | 10.4 |
| CS11 | 87202060 | 86596986 | 99.31 | 96.59 | 93.42 | 68.3 | 4.61 | 12.16 |
| CS10 | 76281990 | 75739947 | 99.29 | 96.47 | 93.34 | 56.82 | 1.87 | 10.73 |
| CS9 | 147961850 | 147064091 | 99.39 | 97.13 | 95.86 | 92.17 | 54.13 | 20.69 |
| CS8 | 85890256 | 85274751 | 99.28 | 95.95 | 93.69 | 68.19 | 3.66 | 12.07 |
| CS7 | 78726738 | 78199492 | 99.33 | 96.74 | 93.41 | 60.32 | 2.48 | 11.12 |
| CS6 | 89601112 | 88812730 | 99.12 | 96.27 | 94 | 67.14 | 6.31 | 12.47 |
| CS5 | 89582908 | 88946913 | 99.29 | 96.64 | 93.59 | 69.94 | 5.87 | 12.51 |
| CS4 | 74369964 | 73879149 | 99.34 | 95.96 | 92.69 | 54.09 | 1.66 | 10.47 |
| CS3 | 72206882 | 71742759 | 99.36 | 95.9 | 92.46 | 50.81 | 1.42 | 10.19 |
| CS2 | 84119714 | 83520735 | 99.29 | 96.61 | 93.13 | 64.94 | 3.65 | 11.73 |
| PD1 | 103820794 | 102728610 | 98.95 | 96.95 | 94.6 | 77.69 | 12.18 | 14.18 |
| PD2 | 89496906 | 88749360 | 99.16 | 96.26 | 94.09 | 69.3 | 5.58 | 12.51 |
| PD3 | 149038712 | 148014804 | 99.31 | 96.52 | 95.4 | 92.2 | 53.22 | 20.91 |
| PD4 | 89050902 | 88078560 | 98.91 | 96.77 | 91.79 | 59.87 | 10.59 | 12.35 |
| PD5 | 104526776 | 103523940 | 99.04 | 96.53 | 95.09 | 80.65 | 9.29 | 14.13 |
| PD6 | 127895182 | 126990880 | 99.29 | 96.51 | 95.23 | 89.59 | 33.75 | 18.01 |
| PD7 | 111518842 | 110691435 | 99.26 | 96.97 | 95.09 | 82.45 | 15.78 | 15 |
| PD8 | 83659938 | 83075494 | 99.30 | 96.71 | 93.17 | 63.85 | 4.41 | 11.77 |
| PD9 | 106346590 | 104814973 | 98.56 | 96.48 | 92.33 | 60.3 | 18.84 | 14.46 |
| PD10 | 75391350 | 74826673 | 99.25 | 96.05 | 92.87 | 55.25 | 1.84 | 10.62 |
| PD11 | 73605068 | 73019653 | 99.20 | 96.52 | 91.16 | 52 | 2.13 | 10.33 |
| PD12 | 96193058 | 95509155 | 99.29 | 96.65 | 93.98 | 75.25 | 8.86 | 13.42 |
| PD13 | 78224762 | 77617944 | 99.13 | 95.45 | 76.89 | 12.48 | 0.37 | 10.92 |
| PD14 | 91169220 | 90421307 | 99.18 | 96.67 | 93.29 | 68.82 | 7.77 | 12.72 |
| PD15 | 80928656 | 80176938 | 99.07 | 96.62 | 92 | 57.86 | 4.68 | 11.29 |
| PD16 | 95120790 | 94327172 | 99.17 | 96.76 | 93.25 | 70.32 | 11.19 | 13.31 |
| PD17 | 95820120 | 95016536 | 99.16 | 96.72 | 93.54 | 71.95 | 10.48 | 13.41 |
| PD18 | 78948154 | 78308165 | 99.19 | 96.61 | 92.3 | 57.85 | 3.01 | 11.04 |
| PD19 | 100180134 | 99262689 | 99.08 | 96.87 | 94.32 | 75.31 | 11.69 | 13.87 |
| PD20 | 138133822 | 136939801 | 99.14 | 97.24 | 95.85 | 90.79 | 36.22 | 18.47 |
| PD21 | 77293808 | 76650670 | 99.17 | 96.49 | 91.51 | 55.98 | 2.94 | 10.82 |
| PD22 | 72030296 | 71519207 | 99.29 | 95.92 | 91.94 | 49.61 | 1.69 | 10.16 |
| PD23 | 76059326 | 75587670 | 99.38 | 95.91 | 92.66 | 56.99 | 1.76 | 10.73 |
| PD25 | 71534156 | 70942593 | 99.26 | 95.9 | 91.01 | 38.11 | 0.92 | 9.85 |
| PD26 | 67470662 | 66628549 | 98.75 | 96.65 | 87.5 | 37.03 | 3.57 | 9.3 |
| PD24 | 82189076 | 81557536 | 99.23 | 96.59 | 92.46 | 61.68 | 3.95 | 11.5 |
| PD27 | 76152334 | 75100126 | 98.62 | 96.05 | 86.69 | 40.32 | 7.02 | 10.2 |
| PD28 | 66599666 | 65938283 | 99.01 | 96.66 | 88.59 | 34.93 | 1.36 | 8.76 |
| PD29 | 91381402 | 90477503 | 99.01 | 96.75 | 93.04 | 64.76 | 8.68 | 12.53 |
| PD30 | 121162720 | 119961087 | 99.01 | 96.58 | 95.27 | 87.37 | 23.45 | 16.56 |
| WM1 | 77152586 | 76440490 | 99.08 | 96.66 | 92.09 | 55.05 | 2.79 | 10.76 |
| WM2 | 84856806 | 84230787 | 99.26 | 96.73 | 93.27 | 66.39 | 3.99 | 11.9 |
| WM3 | 75999965 | 75452642 | 99.28 | 96.56 | 92.28 | 56.47 | 1.88 | 10.67 |
| WM4 | 89598310 | 88939461 | 99.26 | 96.7 | 93.74 | 70.44 | 5.32 | 12.51 |
| WM5 | 95400630 | 94693651 | 99.26 | 96.8 | 94.22 | 74.54 | 7.74 | 13.29 |
| WM6 | 80184390 | 79606044 | 99.32 | 95.94 | 92.38 | 49.62 | 1.19 | 11.32 |
| WM7 | 92248590 | 91527621 | 99.22 | 96.7 | 93.92 | 72.83 | 6.52 | 12.89 |
| WM8 | 107722732 | 106749781 | 99.10 | 96.35 | 94.8 | 82.76 | 12.02 | 14.7 |
| WM9 | 72220232 | 71660544 | 99.23 | 96.42 | 91.44 | 50.8 | 1.42 | 10.1 |
| WM10 | 92637524 | 91735348 | 99.03 | 97 | 94.22 | 71.04 | 5.58 | 12.61 |
| WM11 | 82701626 | 82027300 | 99.18 | 96.16 | 92.94 | 61.16 | 5.27 | 11.75 |
| WM12 | 102971356 | 102005149 | 99.06 | 96.38 | 94.7 | 80.76 | 9.52 | 14.11 |
| WM13 | 83878466 | 83206138 | 99.20 | 96.18 | 93.82 | 64.8 | 3.45 | 11.78 |
| WM14 | 77317228 | 76731332 | 99.24 | 96.46 | 92.16 | 57.83 | 2.18 | 10.84 |
| WM15 | 76207180 | 75719135 | 99.36 | 95.94 | 92.72 | 57.57 | 1.85 | 10.79 |
| WM16 | 130537530 | 129701312 | 99.36 | 97.02 | 95.34 | 88.77 | 38.57 | 18.26 |
| WM17 | 94617136 | 93979162 | 99.33 | 96.58 | 93.64 | 73.7 | 8.41 | 13.23 |
| WM18 | 80873912 | 80358293 | 99.36 | 95.86 | 92.8 | 62.55 | 2.79 | 11.38 |
| WM19 | 103863908 | 102910627 | 99.08 | 96.45 | 94.92 | 81.44 | 9.59 | 14.23 |
| WM20 | 66494784 | 65910063 | 99.12 | 93.94 | 61.24 | 4.59 | 0.49 | 4.97 |
| WM21 | 78623832 | 78148073 | 99.39 | 95.8 | 92.51 | 60.13 | 2.33 | 11.08 |
| WM22 | 108622950 | 107870072 | 99.31 | 96.96 | 94.83 | 81.88 | 17.89 | 15.22 |
| WM24 | 132413814 | 131538923 | 99.34 | 97.08 | 95.52 | 90.36 | 39.62 | 18.58 |
| WM25 | 84131224 | 83524831 | 99.28 | 96.58 | 93.06 | 65.48 | 3.71 | 11.79 |
| WM26 | 89687002 | 89012140 | 99.25 | 96.1 | 93.41 | 70.34 | 5.12 | 12.41 |
| WM23 | 108754102 | 108058425 | 99.36 | 96.36 | 94.73 | 84.83 | 15.91 | 15.31 |
| WM27 | 85137460 | 84586678 | 99.35 | 95.98 | 93.42 | 67.49 | 3.68 | 11.98 |
| WM28 | 85000036 | 84409313 | 99.31 | 96.51 | 92.87 | 65.69 | 4.25 | 11.89 |
| WM29 | 94199652 | 93532860 | 99.29 | 96.71 | 93.73 | 72.24 | 7.85 | 12.98 |
| WM30 | 80002160 | 79492155 | 99.36 | 95.92 | 93.06 | 62.11 | 2.41 | 11.29 |
| WM31 | 87591630 | 87048497 | 99.38 | 96.06 | 93.52 | 70.04 | 4.62 | 12.35 |
| CSWG1 | 78610156 | 78108645 | 99.36 | 95.9 | 92.86 | 59.76 | 2.16 | 11.06 |
| CSWG2 | 73092510 | 72580374 | 99.30 | 96.4 | 91.29 | 52.92 | 1.73 | 10.32 |
| CSWG3 | 85379382 | 84794116 | 99.31 | 96.44 | 92.59 | 65.74 | 4.65 | 11.94 |
| CSWG4 | 121246932 | 120379861 | 99.28 | 97.05 | 95.51 | 88.03 | 27.62 | 17 |
| CSWG5 | 99785720 | 99024353 | 99.24 | 96.43 | 94.6 | 79.51 | 8.93 | 13.83 |
| CSWG6 | 89334474 | 88699960 | 99.29 | 96.2 | 94.17 | 71.53 | 4.68 | 12.55 |
| CSWG7 | 84139088 | 83538131 | 99.29 | 96.09 | 93.77 | 66.56 | 3.22 | 11.85 |
| CSWG8 | 82767670 | 82123728 | 99.22 | 96.53 | 92.79 | 63.32 | 3.32 | 11.55 |
| CSWG9 | 82812310 | 82247555 | 99.32 | 96.02 | 93.35 | 64.73 | 2.97 | 11.64 |
| CSWG10 | 88922344 | 88308435 | 99.31 | 96.08 | 93.81 | 70.69 | 4.6 | 12.49 |
| CSWG11 | 94214702 | 93616158 | 99.36 | 96 | 93.67 | 75.33 | 7.17 | 13.22 |
| CSWG12 | 96417810 | 95815453 | 99.38 | 96.06 | 93.94 | 77.15 | 8.01 | 13.54 |
| CSWG13 | 84221214 | 83677771 | 99.35 | 96.41 | 92.47 | 65 | 4.25 | 11.8 |
| CSWG14 | 74425066 | 73865922 | 99.25 | 96.27 | 91.45 | 54.7 | 1.76 | 10.48 |
| CSWG15 | 112341352 | 111461741 | 99.22 | 96.77 | 94.61 | 81.84 | 19.34 | 15.41 |
| CSWG16 | 116083056 | 115313624 | 99.34 | 96.22 | 94.52 | 86.14 | 21.66 | 16.17 |
| CSWG17 | 111652240 | 110669564 | 99.12 | 96.92 | 95.04 | 82.86 | 18.53 | 15.44 |
| CSWG18 | 101197176 | 100354907 | 99.17 | 96.28 | 94.64 | 80.62 | 9.7 | 14.15 |
| CSWG19 | 114989926 | 113987281 | 99.13 | 96.87 | 94.99 | 83.19 | 21.68 | 15.86 |
| CSWG20 | 109585684 | 108491104 | 99.00 | 96.17 | 93.91 | 64.76 | 3.71 | 12.11 |
| CSWG21 | 117454002 | 116302996 | 99.02 | 97.08 | 95.31 | 84.09 | 20.9 | 15.88 |
| CSWG22 | 100452884 | 99557948 | 99.11 | 96.91 | 94.32 | 76 | 9.92 | 13.69 |
| CSWG23 | 101185698 | 100350619 | 99.17 | 96.3 | 94.35 | 79.06 | 8.82 | 13.86 |
| CSWG24 | 104406702 | 103441100 | 99.08 | 97 | 94.65 | 78.33 | 11.89 | 14.21 |
| CSWG25 | 91586358 | 90945443 | 99.30 | 96.55 | 93.13 | 70.9 | 7.31 | 12.79 |
| CSWG26 | 104633874 | 103704920 | 99.11 | 96.94 | 94.42 | 77.47 | 12.79 | 14.2 |
| CSWG27 | 100028954 | 99114069 | 99.09 | 96.9 | 94.2 | 75.51 | 9.76 | 13.61 |
| CSWG28 | 105039744 | 104174674 | 99.18 | 96.36 | 94.35 | 80.72 | 12.1 | 14.44 |
| CSWG29 | 89533184 | 88840338 | 99.23 | 96.23 | 93.6 | 69.39 | 4.71 | 12.31 |
| CSWG30 | 113410360 | 112438858 | 99.14 | 96.52 | 95.13 | 85.99 | 15.98 | 15.51 |
| XY23 | 120013942 | 118711345 | 98.93 | 96.72 | 91.14 | 44.6 | 1.13 | 16.22 |
| XY30 | 89055762 | 88444000 | 99.31 | 96.77 | 94.05 | 70.53 | 5.24 | 12.47 |
| XY29 | 75887452 | 75338525 | 99.28 | 95.92 | 92.97 | 55.71 | 1.74 | 10.68 |
| XY28 | 97480530 | 96776690 | 99.28 | 96.15 | 94.42 | 77.95 | 7.79 | 13.66 |
| XY27 | 92716168 | 91923572 | 99.15 | 96.29 | 94.31 | 72.76 | 5.14 | 12.72 |
| XY26 | 76055316 | 75505074 | 99.28 | 95.95 | 93 | 55.58 | 1.79 | 10.71 |
| XY25 | 77805172 | 77251194 | 99.29 | 96.61 | 92.46 | 57.8 | 2.35 | 10.89 |
| XY24 | 102752374 | 101669906 | 98.95 | 97.09 | 94.9 | 77.09 | 10.47 | 13.93 |
| XY22 | 113466890 | 112417956 | 99.08 | 97.05 | 95.24 | 82.97 | 17.92 | 15.43 |
| XY21 | 78788488 | 78119267 | 99.15 | 96.07 | 92.53 | 56.76 | 3.93 | 11.15 |
| XY20 | 76640592 | 76069205 | 99.25 | 96.57 | 92.3 | 56 | 2.06 | 10.71 |
| XY19 | 83298788 | 82716285 | 99.30 | 96.03 | 93.66 | 64.88 | 3.01 | 11.68 |
| XY17 | 51815316 | 51487547 | 98.77 | 85.59 | 45.25 | 3.34 | 0.26 | 4.26 |
| XY16 | 78114120 | 77606760 | 99.35 | 95.94 | 92.04 | 45.25 | 1.08 | 9.66 |
| XY18 | 82823878 | 82251496 | 99.31 | 96.04 | 93.64 | 64.35 | 2.96 | 11.62 |
| XY15 | 95454450 | 94532165 | 99.03 | 97 | 94.45 | 73.33 | 6.72 | 13.01 |
| XY14 | 105764102 | 104899740 | 99.18 | 96.82 | 94.76 | 80.59 | 13.97 | 14.67 |
| XY13 | 87854378 | 87136679 | 99.18 | 96.7 | 93.62 | 68.4 | 4.7 | 12.23 |
| XY12 | 124369570 | 123507758 | 99.31 | 97 | 95.28 | 89.53 | 29.85 | 17.4 |
| XY7 | 77066631 | 76464319 | 99.22 | 96.56 | 92.26 | 56.65 | 2.2 | 10.78 |
| XY11 | 93254076 | 92599142 | 99.30 | 96.09 | 94.1 | 74.82 | 6.47 | 13.09 |
| XY10 | 73198602 | 72680881 | 99.29 | 96.5 | 91.74 | 51.95 | 1.56 | 10.24 |
| XY9 | 77803096 | 77210866 | 99.24 | 96.52 | 92.39 | 57.89 | 2.23 | 10.86 |
| XY8 | 103276598 | 102265109 | 99.02 | 96.98 | 94.46 | 72.66 | 6.28 | 12.88 |
| XY6 | 82808316 | 82251334 | 99.33 | 96.04 | 93.6 | 64.26 | 2.94 | 11.62 |
| XY5 | 92313614 | 91689700 | 99.32 | 96.11 | 94.14 | 73.63 | 5.83 | 12.93 |
| XY4 | 82719350 | 82101994 | 99.25 | 96.67 | 92.89 | 62.42 | 3.71 | 11.55 |
| XY3 | 82537676 | 82020598 | 99.37 | 96.01 | 93.55 | 63.72 | 2.95 | 11.56 |
| XY2 | 86918052 | 86268267 | 99.25 | 96.62 | 93.36 | 67.2 | 4.71 | 12.14 |
| XY1 | 125184852 | 124445659 | 99.41 | 96.43 | 95.03 | 89.08 | 27.61 | 17.16 |
| YS1 | 99340016 | 98349289 | 99.06 | 96 | 89.72 | 30.76 | 0.62 | 13.59 |
| YS2 | 99874446 | 98837760 | 98.96 | 96.51 | 94.91 | 75.57 | 8.9 | 13.6 |
| YS3 | 89334178 | 88434937 | 98.99 | 96.95 | 93.98 | 68.28 | 4.39 | 12.2 |
| YS4 | 106552282 | 105563221 | 99.07 | 97.02 | 94.82 | 79.67 | 12.97 | 14.5 |
| YS5 | 101087114 | 100120910 | 99.04 | 96.9 | 94.28 | 76.05 | 10.68 | 13.77 |
| YS6 | 103015298 | 102136837 | 99.20 | 96.02 | 91.79 | 49.46 | 1.31 | 14.08 |
| YS7 | 79239926 | 78587811 | 99.18 | 96.21 | 93.2 | 58.39 | 1.98 | 10.94 |
| YS8 | 130156064 | 129243733 | 99.30 | 96.28 | 94.69 | 89.11 | 35.7 | 18.07 |
| YS9 | 86261182 | 85636814 | 99.28 | 96.65 | 93.3 | 67.42 | 4.39 | 12.05 |
| YS10 | 108400910 | 107464659 | 99.14 | 96.44 | 94.87 | 83.63 | 12.92 | 14.82 |
| YS11 | 112274632 | 111155389 | 99.00 | 96.51 | 95.2 | 85.09 | 14.47 | 15.26 |
| YS12 | 94201490 | 93362130 | 99.11 | 96.44 | 94.68 | 74.46 | 5.28 | 12.93 |
| YS13 | 138255036 | 137218599 | 99.25 | 96.6 | 95.21 | 91.14 | 44.03 | 19.22 |
| YS14 | 104804582 | 104048573 | 99.28 | 96.68 | 94.15 | 79.03 | 14.77 | 14.51 |
| YS15 | 104545588 | 103728686 | 99.22 | 96.79 | 94.19 | 77.24 | 12.94 | 14.04 |
| YS16 | 124728022 | 123477935 | 99.00 | 96.54 | 95.27 | 85.36 | 15.13 | 15.44 |
| YS17 | 141617234 | 140032444 | 98.88 | 96.63 | 95.5 | 90.42 | 29.5 | 17.72 |
| YS18 | 106731258 | 105796951 | 99.12 | 96.88 | 94.46 | 78.16 | 17.43 | 14.84 |
| YS19 | 93300616 | 92203570 | 98.90 | 96.99 | 93.72 | 62.96 | 3.06 | 12.59 |
| YS20 | 103972134 | 102971354 | 99.04 | 95.99 | 94.18 | 78.18 | 10.53 | 13.98 |
| YS21 | 94992578 | 93939707 | 98.93 | 96.3 | 84.26 | 19.78 | 0.52 | 12.88 |
| YS22 | 96996918 | 96012558 | 98.99 | 96.51 | 94.8 | 74.09 | 7.79 | 13.31 |
| YS23 | 95013516 | 94092074 | 99.03 | 96.46 | 94.4 | 70.91 | 8.19 | 13.07 |
| YS24 | 97203148 | 96269966 | 99.04 | 96.93 | 94.18 | 73.85 | 8.21 | 13.25 |
| YS25 | 98327066 | 97410144 | 99.07 | 96.99 | 94.42 | 74.98 | 8.52 | 13.4 |
| YS26 | 67318950 | 66816328 | 99.25 | 96.65 | 91.49 | 43.64 | 1.17 | 9.5 |
| YS27 | 110788612 | 109984256 | 99.27 | 96.42 | 94.89 | 84 | 18.57 | 15.58 |
| YS28 | 88513096 | 87856053 | 99.26 | 96.61 | 93.35 | 69.36 | 5.39 | 12.38 |
| YS29 | 78209244 | 77596968 | 99.22 | 96.53 | 92.01 | 57.93 | 2.82 | 10.98 |
| QD1 | 99640512 | 98715606 | 99.07 | 96.98 | 94.61 | 76.14 | 8.83 | 13.54 |
| QD2 | 79589226 | 78975974 | 99.23 | 96.6 | 92.7 | 60.09 | 2.59 | 11.14 |
| QD3 | 80269304 | 79719199 | 99.31 | 96.02 | 93.37 | 62.1 | 2.41 | 11.29 |
| QD4 | 75523806 | 74973666 | 99.27 | 96.57 | 92.19 | 55.72 | 1.88 | 10.59 |
| QD5 | 83602132 | 83023108 | 99.31 | 96.17 | 93.77 | 65.95 | 3.14 | 11.76 |
| QD6 | 92740294 | 92002991 | 99.20 | 96.8 | 93.92 | 72.64 | 6.96 | 12.95 |
| QD7 | 90260208 | 89606884 | 99.28 | 96.15 | 94.16 | 71.89 | 4.92 | 12.65 |
| QD8 | 90085880 | 89459541 | 99.30 | 96.12 | 94.09 | 72.41 | 5.15 | 12.65 |
| QD9 | 83496604 | 82893665 | 99.28 | 96.05 | 93.68 | 65.27 | 3.05 | 11.71 |
| QD10 | 107256292 | 106572718 | 99.36 | 96.26 | 94.67 | 83.94 | 14.21 | 15.02 |
| QD11 | 77877094 | 77197539 | 99.13 | 96.56 | 92.37 | 56.56 | 2.01 | 10.76 |
| QD12 | 81715246 | 81073210 | 99.21 | 96.61 | 92.92 | 62.58 | 3.03 | 11.4 |
| QD13 | 128573926 | 127144385 | 98.89 | 97.27 | 95.65 | 83.75 | 28.34 | 16.93 |
| QD14 | 76269564 | 75749337 | 99.32 | 96.03 | 93.08 | 56.6 | 1.86 | 10.75 |
| QD15 | 90518356 | 89841965 | 99.25 | 96.67 | 93.75 | 71.1 | 5.86 | 12.63 |
| QD16 | 153122092 | 152040727 | 99.29 | 97.19 | 96.03 | 92.56 | 57 | 21.3 |
| QD17 | 80824778 | 80295479 | 99.35 | 96.04 | 93.48 | 62.61 | 2.63 | 11.38 |
| QD18 | 99751296 | 98884797 | 99.13 | 96.44 | 94.82 | 78.44 | 7.73 | 13.65 |
| QD19 | 72592012 | 72028830 | 99.22 | 96.09 | 91.31 | 38.43 | 0.8 | 9.07 |
| QD20 | 91632526 | 90944718 | 99.25 | 96.23 | 94.37 | 73.22 | 4.96 | 12.74 |
| QD21 | 93427968 | 92688386 | 99.21 | 96.72 | 93.95 | 72.84 | 6.88 | 13.02 |
| QD22 | 88789934 | 88075747 | 99.20 | 96.68 | 93.63 | 69.28 | 5.01 | 12.37 |
| QD23 | 89055210 | 88427840 | 99.30 | 96.09 | 94.01 | 71.32 | 4.55 | 12.5 |
| QD24 | 78448004 | 77902996 | 99.31 | 96.58 | 92.53 | 59.14 | 2.39 | 10.99 |
| QD25 | 77274572 | 76749714 | 99.32 | 95.99 | 93.15 | 57.96 | 2 | 10.89 |
| QD26 | 80128344 | 79613129 | 99.36 | 96.59 | 93.67 | 62.6 | 2.48 | 11.32 |
| QD27 | 78904494 | 78429553 | 99.40 | 96.15 | 93.52 | 60.87 | 2.22 | 11.18 |
| QD28 | 74214828 | 73659041 | 99.25 | 96.5 | 91.85 | 53.83 | 1.66 | 10.39 |
| QD29 | 143634620 | 142596581 | 99.28 | 97.15 | 95.93 | 91.83 | 49.28 | 20.04 |
| QD30 | 73808294 | 73255292 | 99.25 | 96.55 | 91.81 | 52.27 | 1.6 | 10.29 |
| RJF1 | 177458494 | 167338617 | 94.30 | 96.93 | 94.77 | 77.99 | 13.94 | 14.71 |
| RJF10 | 107074266 | 104408896 | 97.51 | 97.4 | 94.52 | 67.7 | 4.88 | 12.43 |
| RJF11 | 100610416 | 98374717 | 97.78 | 97.38 | 94.09 | 62.99 | 3.21 | 11.71 |
| RJF2 | 136399602 | 133585398 | 97.94 | 97.52 | 94.53 | 67.57 | 5.41 | 12.33 |
| RJF3 | 186436306 | 180874931 | 97.02 | 96.23 | 94.1 | 76.74 | 28.95 | 16.66 |
| RJF4 | 223851144 | 217852802 | 97.32 | 96.37 | 95.15 | 91.28 | 44.3 | 19.93 |
| RJF5 | 343599651 | 335782509 | 97.72 | 96.63 | 95.72 | 94.47 | 87.97 | 31.41 |
| RJF7 | 102128838 | 100234679 | 98.15 | 96.8 | 94.57 | 66.28 | 3.57 | 12.02 |
| RJF8 | 96633760 | 94541089 | 97.83 | 96.65 | 94.03 | 60.4 | 2.56 | 11.36 |
| RJF9 | 94642100 | 92922579 | 98.18 | 96.83 | 94.37 | 58.98 | 2.17 | 11.18 |

1) Clean reads: The total number of reads of valid sequenced data. 2) Mapped reads: The number of reads mapped to the reference (including both single-end comparison and double-end comparison). 3) Mapping rate: The proportion of the reads number mapped to the reference genome and the total reads number of valid sequenced data. 4) Average depth: The proportion of the total number of bases compared to the reference genome and the whole genome bases. 5) Coverage at least 1X: the percentage of the reference genome with at least one base coverage. 6) Coverage at least 4X: the percentage of the reference genome with at least four base coverage sites. 7) Coverage at least 10X: the percentage of the reference genome with at least ten base coverage sites. 8) Coverage at least 20X: the percentage of the reference genome with at least twenty base coverage.

Table S3 Annotation information of SNPs

| Category | | Number of SNPs | | | | | | | | | | | |
| --- | --- | --- | --- | --- | --- | --- | --- | --- | --- | --- | --- | --- | --- |
| Breed | | All | CS | CSWG | PD | QD | WM | WN | XY | YS | SHXY | TF | RJF |
| Upstream | | 114264 | 101057 | 96590 | 97242 | 99687 | 98739 | 105062 | 91667 | 99226 | 80864 | 97722 | 90657 |
| Extron | UTR3 | 69619 | 61152 | 58546 | 58215 | 60441 | 59851 | 63687 | 55280 | 60023 | 46070 | 57472 | 55528 |
|  | UTR5 | 12673 | 11151 | 10576 | 10647 | 10871 | 10928 | 11565 | 10080 | 10914 | 8787 | 10693 | 9862 |
|  | UTR5; UTR3 | 368 | 322 | 319 | 303 | 333 | 314 | 339 | 295 | 319 | 251 | 323 | 281 |
|  | Stop gain | 297 | 255 | 249 | 239 | 245 | 237 | 262 | 235 | 240 | 202 | 244 | 209 |
|  | Stop loss | 18 | 15 | 13 | 12 | 14 | 15 | 15 | 15 | 15 | 8 | 11 | 14 |
|  | Synonymous | 79045 | 69729 | 66076 | 66625 | 68752 | 67986 | 72434 | 63407 | 68134 | 54210 | 66671 | 61611 |
|  | Non-synonymous | 30825 | 26262 | 24960 | 25060 | 25903 | 25880 | 27788 | 23806 | 25571 | 20280 | 25321 | 22252 |
|  | unknown | 12 | 10 | 12 | 7 | 11 | 11 | 12 | 6 | 11 | 6 | 10 | 3 |
| Intronic  Splicing | | 3853625 | 3417661 | 3277257 | 3262658 | 3369912 | 3363458 | 3545703 | 3101606 | 3364282 | 2613145 | 3223904 | 3181498 |
|  |  | 208 | 179 | 175 | 174 | 176 | 183 | 189 | 171 | 184 | 145 | 167 | 172 |
| Downstream | | 137980 | 121921 | 116772 | 116440 | 120548 | 119269 | 126611 | 110640 | 120123 | 95044 | 116128 | 111229 |
| Upstream/Downstream | | 7929 | 7051 | 6621 | 6794 | 6975 | 6816 | 7290 | 6381 | 6887 | 5611 | 6840 | 6123 |
| Intergenic | | 3008733 | 2653908 | 2554442 | 2545026 | 2620468 | 2616912 | 2760128 | 2408817 | 2621506 | 2075954 | 2532272 | 2463498 |
| Other | | 829444 | 731023 | 706603 | 700710 | 723115 | 720534 | 760540 | 666247 | 723103 | 565898 | 694221 | 682712 |
| Het | | 2469103 | 2297615 | 2251257 | 1863185 | 2280332 | 2334250 | 2438481 | 3700818 | 2053043 | 2484282 | 2279923 | 2132988 |
| Hom | | 5556070 | 4598547 | 4138170 | 4951688 | 4685895 | 4369444 | 4934939 | 2512139 | 3259627 | 2952425 | 4504662 | 4333704 |
| Hom/Het | | 2.2502 | 2.0014 | 1.8382 | 2.6576 | 2.0549 | 1.8719 | 2.0238 | 0.6788 | 1.5877 | 1.1884 | 1.9758 | 2.0318 |
| Ts | | 5924255 | 5241609 | 5035241 | 5016895 | 5173418 | 5159618 | 5444288 | 4760250 | 5167879 | 4060018 | 4977493 | 4865368 |
| Tv | | 2220785 | 1960087 | 1883970 | 1873256 | 1934033 | 1931515 | 2037337 | 1778403 | 1932659 | 1506457 | 1854506 | 1820281 |
| Ts/Tv | | 2.667 | 2.674 | 2.672 | 2.678 | 2.674 | 2.671 | 2.672 | 2.676 | 2.673 | 2.695 | 2.683 | 2.672 |
| Total | | 8145040 | 7201696 | 6919211 | 6890151 | 7107451 | 7091133 | 7481625 | 6538653 | 7100538 | 5566475 | 6831999 | 6685649 |

1) Total: the total numbers of SNPs. 2) Upstream: SNPs in the 1 kb upstream regions of genes. 3) Extron: SNPs in the Extron regions. 4) Stop gain: SNPs bring termination codon to gene. 5) Stop loss: SNPs delete the termination codon of gene. Intronic: SNPs in the intronic regions. 6) Splicing: SNPs in the intron regions which are 2 bp near the exon/intron boundary. 7) Downstream: SNPs in the 1 kb downstream regions of genes. Upstream/Downstream: SNPs in the regions which are both belonged to 1 kb upstream regions of genes and 1 kb downstream regions of genes. 8) Intergenic: SNPs in the intergenic regions. 9) Heterozygote. 10) Homozygous.

Table S4 Annotation information of specific SNPs in each breed

| Category | | Number of specific SNPs | | | | | | | | | | |
| --- | --- | --- | --- | --- | --- | --- | --- | --- | --- | --- | --- | --- |
| Breed | | CS | CSWG | PD | QD | WM | WN | XY | YS | SHXY | TF | RJF |
| Upstream | | 7705 | 6414 | 7301 | 7445 | 6595 | 7822 | 5989 | 7460 | 3561 | 5829 | 6479 |
| Extron | UTR3 | 4300 | 3589 | 4079 | 4270 | 3512 | 4328 | 3347 | 4140 | 1773 | 3012 | 3634 |
|  | UTR5 | 791 | 679 | 768 | 753 | 671 | 824 | 635 | 789 | 374 | 607 | 646 |
|  | UTR5; UTR3 | 30 | 23 | 24 | 25 | 21 | 27 | 18 | 25 | 9 | 24 | 18 |
|  | Stop gain | 47 | 48 | 43 | 44 | 36 | 44 | 33 | 40 | 28 | 39 | 35 |
|  | Stop loss | 3 | 2 | 2 | 3 | 4 | 4 | 4 | 3 | 4 | 1 | 5 |
|  | Synonymous | 4542 | 3719 | 4475 | 4541 | 3748 | 4652 | 3597 | 4426 | 1964 | 3302 | 3803 |
|  | Non-synonymous | 2682 | 2350 | 2680 | 2746 | 2431 | 2823 | 2233 | 2628 | 1445 | 2131 | 2011 |
|  | unknown | 5 | 6 | 1 | 5 | 5 | 6 | - | 5 | - | 4 | - |
| Intronic  Splicing | | 219595 | 182627 | 212817 | 216191 | 185152 | 223238 | 167398 | 213572 | 90158 | 153418 | 198299 |
|  |  | 16 | 16 | 14 | 14 | 13 | 15 | 14 | 17 | 8 | 10 | 18 |
| Downstream | | 8643 | 7267 | 8326 | 8529 | 7220 | 8804 | 6702 | 8454 | 3831 | 6287 | 7568 |
| upstream/downstream | | 666 | 591 | 644 | 671 | 573 | 670 | 539 | 638 | 351 | 543 | 543 |
| Intergenic | | 179620 | 152879 | 176031 | 178984 | 153067 | 182942 | 138031 | 176205 | 79319 | 127880 | 160258 |
| Other | | 45966 | 39155 | 44171 | 46188 | 38940 | 46893 | 34933 | 45141 | 18443 | 31782 | 41670 |
| Het | | 93760 | 96590 | 108499 | 118795 | 97801 | 104032 | 150886 | 102109 | 76131 | 72449 | 96038 |
| Hom | | 358841 | 268790 | 346664 | 341150 | 283116 | 370858 | 196444 | 240055 | 120718 | 258715 | 315172 |
| Hom/Het | | 3.8272 | 2.7828 | 3.195 | 2.8718 | 2.8948 | 3.5648 | 1.3019 | 2.3510 | 1.5857 | 3.5710 | 3.2817 |
| Ts | | 336284 | 281950 | 327101 | 333217 | 283712 | 341924 | 256670 | 328375 | 140509 | 235878 | 302180 |
| Tv | | 138327 | 117415 | 134275 | 137192 | 118276 | 141168 | 106803 | 135168 | 60759 | 98991 | 122807 |
| Ts/Tv | | 2.431 | 2.401 | 2.436 | 2.428 | 2.398 | 2.422 | 2.403 | 2.429 | 2.312 | 2.382 | 2.46 |
| Total | | 474611 | 399365 | 461376 | 470409 | 401988 | 483092 | 363473 | 463543 | 201268 | 334869 | 424987 |

Table S5 The pairwise Fst, DR and Nm results

| **DR\Fst** | WN | SHXY | XY | CS | TF | WM | PD | CSWG | RJF | QD | YS |
| --- | --- | --- | --- | --- | --- | --- | --- | --- | --- | --- | --- |
| WN |  | **0.154246** | 0.041278 | 0.048166 | 0.128688 | 0.055927 | 0.010209 | 0.041058 | 0.051205 | 0.038801 | 0.062682 |
| SHXY | 0.098616 |  | 0.142681 | **0.170089** | **0.161155** | **0.151001** | 0.0894 | **0.16569** | 0.141945 | 0.142406 | **0.176756** |
| XY | 0.061165 | 0.11435 |  | 0.059093 | 0.123461 | 0.059814 | 0.019462 | 0.056084 | 0.061758 | 0.050777 | 0.073905 |
| CS | 0.047149 | 0.104314 | 0.063769 |  | **0.150933** | 0.06951 | 0.029411 | 0.065223 | 0.075517 | 0.066185 | 0.088188 |
| TF | 0.079672 | 0.113693 | 0.099674 | 0.083425 |  | 0.137172 | 0.075677 | 0.146669 | 0.137787 | 0.12581 | **0.157358** |
| WM | 0.042449 | 0.102989 | 0.068272 | 0.05801 | 0.085807 |  | 0.022812 | 0.064647 | 0.072462 | 0.057751 | 0.084598 |
| PD | 0.060883 | 0.120497 | 0.076606 | 0.062083 | 0.105665 | 0.071063 |  | 0.022794 | 0.030429 | 0.022655 | 0.041164 |
| CSWG | 0.050598 | 0.105781 | 0.066986 | 0.054214 | 0.089619 | 0.056995 | 0.060821 |  | 0.070901 | 0.053568 | 0.081429 |
| RJF | 0.067121 | **0.123142** | 0.081767 | 0.067801 | 0.10918 | 0.075273 | 0.076951 | 0.070597 |  | 0.071863 | 0.092856 |
| QD | 0.052121 | 0.113326 | 0.067702 | 0.051852 | 0.097119 | 0.060806 | 0.063686 | 0.058111 | 0.067988 |  | 0.083302 |
| YS | 0.050345 | 0.110944 | 0.065892 | 0.051089 | 0.090664 | 0.057293 | 0.063442 | 0.053588 | 0.064381 | 0.051264 |  |
| **Nm** | WN | SHXY | XY | CS | TF | WM | PD | CSWG | RJF | QD | YS |
| WN |  | 1.370783 | 5.806538 | 4.940414 | 1.692681 | 4.220108 | **24.23758** | 5.838944 | 4.632355 | 6.193157 | 3.738414 |
| SHXY |  |  | 1.502163 | 1.219821 | 1.301302 | 1.405616 | 2.546423 | 1.258838 | 1.511248 | 1.50554 | 1.164379 |
| XY |  |  |  | 3.980632 | 1.774927 | 3.929635 | 12.59533 | 4.207627 | 3.798091 | 4.673469 | 3.132731 |
| CS |  |  |  |  |  | 3.346629 | 8.25015 | 3.582976 | 3.060516 | 3.5273 | 2.584838 |
| TF |  |  |  |  |  | 1.572523 | 3.053496 | 1.45452 | 1.564394 | 1.737122 | 1.338738 |
| WM |  |  |  |  |  |  | 10.70918 | 3.617158 | 3.200079 | 4.07894 | 2.705147 |
| PD |  |  |  |  |  |  |  | 10.71766 | 7.965843 | 10.78523 | 5.823297 |
| CSWG |  |  |  |  |  |  |  |  | 3.276027 | 4.416924 | 2.820174 |
| RJF |  |  |  |  |  |  |  |  |  | 3.22885 | 2.442355 |
| QD |  |  |  |  |  |  |  |  |  |  | 2.751115 |

Table S6 The number of positive selective genes found by different methods

| Breed | Fst＆θπ | XP-CLR | XP-EHH | Overlap in all methods |
| --- | --- | --- | --- | --- |
| CS | 1106 | 2515 | 3353 | 391 |
| CSWG | 1444 | 2447 | 3296 | 488 |
| PD | 1175 | 2761 | 3625 | 401 |
| QD | 1172 | 2630 | 3450 | 432 |
| YS | 1302 | 2663 | 3542 | 332 |
| WN | 1159 | 2463 | 3349 | 399 |
| XY | 1333 | 2705 | 3412 | 425 |
| YS | 1279 | 2497 | 3475 | 389 |

Table S7 KEGG pathways significantly enriched by putatively selected genes of CS

| Term | ID | P-Value | Input |
| --- | --- | --- | --- |
| Pantothenate and CoA biosynthesis | gga00770 | 3.64E-05 | *BCAT1\|PANK1* |
| p53 signaling pathway | gga04115 | 9.49E-05 | *CASP8\|TNR6\|BID\|CDK6* |
| Vascular smooth muscle contraction | gga04270 | 0.000104978 | *ITPR2\|CALD1\|ACTA\|RBM44* |
| 2-Oxocarboxylic acid metabolism | gga01210 | 0.000369066 | *BCAT1* |
| Valine, leucine and isoleucine degradation | gga00280 | 0.002702194 | *BCAT1\|MCEE* |
| Apoptosis | gga04210 | 0.004927979 | *BID\|**CASP8\|TNR6* |
| Steroid biosynthesis | gga00100 | 0.008095637 | *CP51A\|LICH* |
| Pentose and glucuronate interconversions | gga00040 | 0.012007699 | *AK1BA\|ALDR* |
| Protein export | gga03060 | 0.015121746 | *SPCS1\|SRP54* |
| Calcium signaling pathway | gga04020 | 0.027490118 | *ACHA7\|RYR2\|ITPR2\|GRM5\|PLCZ1\|CLTR2* |
| Galactose metabolism | gga00052 | 0.036930937 | *AK1BA\|ALDR* |
| Fructose and mannose metabolism | gga00051 | 0.036930937 | *AK1BA\|ALDR* |
| Biosynthesis of amino acids | gga01230 | 0.043658917 | *BCAT1* |

Table S8 KEGG pathways significantly enriched by putatively selected genes of CSWG

| Term | ID | P-Value | Input |
| --- | --- | --- | --- |
| Pentose and glucuronate interconversions | gga00040 | 5.06E-05 | *ALDR\|AK1BA* |
| p53 signaling pathway | gga04115 | 9.50E-05 | *CCNB2\|BID* |
| Pantothenate and CoA biosynthesis | gga00770 | 0.000112 | *BCAT1\|PANK4* |
| Oocyte meiosis | gga04114 | 0.000278 | *CCNB2\|PRGR\|2A5G\|PLCZ1* |
| Galactose metabolism | gga00052 | 0.000464 | *ALDR\|AK1BA* |
| Fructose and mannose metabolism | gga00051 | 0.000464 | *ALDR\|AK1BA* |
| Glycerolipid metabolism | gga00561 | 0.00076 | *ALDR\|GLPK\|AK1BA* |
| 2-Oxocarboxylic acid metabolism | gga01210 | 0.000903 | *BCAT1* |
| Pentose phosphate pathway | gga00030 | 0.004056 | *DEOC\|G6PI* |
| Phagosome | gga04145 | 0.010657 | *TBA2\|TBB6\|TBA8\|TBB3\|VATE1\|DYHC1\|PLA2R* |
| Apoptosis | gga04210 | 0.014904 | *BID* |
| Vascular smooth muscle contraction | gga04270 | 0.024412 | *KPCE\|CALD1\|RBM44\|RAMP1\|ACTA* |
| Ubiquitin mediated proteolysis | gga04120 | 0.025133 | *WWP2\|UBE2F\|NEDD4* |
| Gap junction | gga04540 | 0.027366 | *TBA2\|TBB6\|TBA8\|TBB3* |
| Valine, leucine and isoleucine degradation | gga00280 | 0.031568 | *BCAT1* |
| Amino sugar and nucleotide sugar metabolism | gga00520 | 0.03877 | *G6PI\|NEUA* |
| Notch signaling pathway | gga04330 | 0.03877 | *MAML2\|HES5* |
| mRNA surveillance pathway | gga03015 | 0.047639 | *2A5G* |

Table S9 KEGG pathways significantly enriched by putatively selected genes of PD

| Term | ID | P-Value | Input |
| --- | --- | --- | --- |
| Wnt signaling pathway | gga04310 | 0.000273 | *DAAM2\|KCC2G\|CUL1\|GSK3B* |
| Ubiquitin mediated proteolysis | gga04120 | 0.000363 | *UBE2F\|HERC2\|CUL1\|FANCL* |
| Protein processing in endoplasmic reticulum | gga04141 | 0.001099 | *MA1B1\|CUL1\|ERO1B\|MARH6\|MA1A2* |
| Melanogenesis | gga04916 | 0.02002 | *KCC2G\|GSK3B\|ASIP* |
| PPAR signaling pathway | gga03320 | 0.020513 | *PPARG* |
| Regulation of actin cytoskeleton | gga04810 | 0.033765 | *ARHG7\|ACTN2\|FINC\|PROF2* |
| Amino sugar and nucleotide sugar metabolism | gga00520 | 0.042924 | *UAP1L1* |
| Hedgehog signaling pathway | gga04340 | 0.045245 | *GSK3B\|GLI3* |
| ErbB signaling pathway | gga04012 | 0.045675 | *GSK3B\|KCC2G* |
| N-Glycan biosynthesis | gga00510 | 0.047627 | *MA1B1\|MA1A2* |

Table S10 KEGG pathways significantly enriched by putatively selected genes of QD

| Term | ID | P-Value | Input |
| --- | --- | --- | --- |
| Ubiquitin mediated proteolysis | gga04120 | 1.22E-07 | *UBE2F\|SAE2\|HERC2\|HERC4\|NEDD4* |
| Drug metabolism - other enzymes | gga00983 | 0.009953 | *SASB* |
| Cardiac muscle contraction | gga04260 | 0.01535 | *COX7R\|RYR2* |
| Cysteine and methionine metabolism | gga00270 | 0.016401 | *SAHHA* |
| Folate biosynthesis | gga00790 | 0.018037 | *DHPR\|MOCS1* |
| Adherens junction | gga04520 | 0.022689 | *CTNA3\|PARD3* |
| FoxO signaling pathway | gga04068 | 0.040753 | *FBX25\|**TGF-β2* |

Table S11 KEGG pathways significantly enriched by putatively selected genes of WM

| Term | ID | P-Value | Input |
| --- | --- | --- | --- |
| Vascular smooth muscle contraction | gga04270 | 0.001495 | *GNAS\|CAC1S\|ACTA* |
| Gap junction | gga04540 | 0.002348 | *TBB6\|GRM1\|GNAS\|TBB3* |
| ECM-receptor interaction | gga04512 | 0.007727 | *CD44\|LAMA3\|ITA4* |
| Calcium signaling pathway | gga04020 | 0.017638 | *GRM1\|RYR2\|GNAS\|CAC1S\|CLTR2* |
| Folate biosynthesis | gga00790 | 0.01994 | *DHPR\|MOCS1* |
| GnRH signaling pathway | gga04912 | 0.039118 | *MK14\|GNAS\|CAC1S* |
| Lysine degradation | gga00310 | 0.039911 | *PLOD2* |
| N-Glycan biosynthesis | gga00510 | 0.042037 | *ALG13* |
| Dorso-ventral axis formation | gga04320 | 0.042827 | *SPIRE2* |
| Fanconi anemia pathway | gga03460 | 0.046455 | *FAN1\|FANCA* |
| Adrenergic signaling in cardiomyocytes | gga04261 | 0.049282 | *MK14\|GNAS\|RYR2\|CAC1S* |

Table S12 KEGG pathways significantly enriched by putatively selected genes of WN

| Term | ID | P-Value | Input |
| --- | --- | --- | --- |
| Wnt signaling pathway | gga04310 | 0.002389 | *DAAM2\|CNBP1\|GSK3B\|KCC2G* |
| Adherens junction | gga04520 | 0.011071 | *PARD3\|CADH1\|CTNA2* |
| Hedgehog signaling pathway | gga04340 | 0.012464 | *GSK3B\|GLI3* |
| Vascular smooth muscle contraction | gga04270 | 0.013565 | *ADCY1\|ACTA* |
| Fanconi anemia pathway | gga03460 | 0.015264 | *FAN1\|FANCA\|CENPS* |
| Intestinal immune network for IgA production | gga04672 | 0.026265 | *SDF1\|TNFSF13B* |
| Folate biosynthesis | gga00790 | 0.026654 | *DHPR\|MOCS1* |
| p53 signaling pathway | gga04115 | 0.031785 | *IGFBP3\|CDK6\|TNR6* |
| Aminoacyl-tRNA biosynthesis | gga00970 | 0.03666 | *SYFM* |

Table S13 KEGG pathways significantly enriched by putatively selected genes of XY

| Term | ID | P-Value | Input |
| --- | --- | --- | --- |
| Pentose and glucuronate interconversions | gga00040 | 0.000893 | *AKR1A1* |
| Wnt signaling pathway | gga04310 | 0.002389 | *NFAC1\|DAAM2\|WNT8C\|FZD1* |
| MAPK signaling pathway | gga04010 | 0.002995 | *NFAC1\|MKNK1\|KS6A3\|TGF-β2* |
| Glycolysis/Gluconeogenesis | gga00010 | 0.017333 | *AKR1A1* |
| Glycerolipid metabolism | gga00561 | 0.017333 | *AKR1A1* |
| SNARE interactions in vesicular transport | gga04130 | 0.020718 | *STX16\|BET1\|SC22B* |
| Non-homologous end-joining | gga03450 | 0.026654 | *DCR1C* |
| Fatty acid biosynthesis | gga00061 | 0.030387 | *FABD\|SAST* |
| RNA transport | gga03013 | 0.038794 | *EIF1B\|RPP38\|IF1AY\|NUPL2* |
| Salmonella infection | gga05132 | 0.040136 | *DC1I1\|WASL\|DC1L1* |
| Valine, leucine and isoleucine degradation | gga00280 | 0.047168 | *3HIDH* |

Table S14 KEGG pathways significantly enriched by putatively selected genes of YS

| Term | ID | P-Value | Input |
| --- | --- | --- | --- |
| Homologous recombination | gga03440 | 6.90E-05 | *EME1\|RAD52* |
| Ribosome biogenesis in eukaryotes | gga03008 | 0.005098 | *HEAT1\|UTP4\|NOB1* |
| Fanconi anemia pathway | gga03460 | 0.008051 | *FANCL\|EME1* |
| Fatty acid biosynthesis | gga00061 | 0.02141 | *ACSL1* |
| Regulation of actin cytoskeleton | gga04810 | 0.022745 | *ARHG7\|ACTN2\|WASL\|ITAV\|ACM3\|FGF9* |
| FoxO signaling pathway | gga04068 | 0.043839 | *FBX25\|IRS2* |
| Calcium signaling pathway | gga04020 | 0.043964 | *IP3KB\|RYR2\|ACM3\|CAC1G* |

Table S15 KEGG pathways significantly enriched by putatively selected genes of egg production

| Term | ID | P-Value | Input |
| --- | --- | --- | --- |
| MAPK signaling pathway | gga04010 | 1.45E-05 | *RASM\|MK01\|PTN7\|RPGF2\|PP2BB\|PAK1* |
| Regulation of actin cytoskeleton | gga04810 | 0.000768 | *RASM\|PI42A\|MK01\|MYPT2\|PAK1* |
| VEGF signaling pathway | gga04370 | 0.003913 | *NFAC\|MK01\|PP2BB* |
| Homologous recombination | gga03440 | 0.004482 | *TOP3B\|RAD50* |
| Adipocytokine signaling pathway | gga04920 | 0.026436 | *AGRP\|ACSL6* |
| Fanconi anemia pathway | gga03460 | 0.037697 | *TOP3B\|EME2* |
| Non-homologous end-joining | gga03450 | 0.044576 | *RAD50* |

Table S16 KEGG pathways significantly enriched by putatively selected genes of growth performance

| Term | ID | P-Value | Input |
| --- | --- | --- | --- |
| Glycosaminoglycan biosynthesis - chondroitin sulfate/dermatan sulfate | gga00532 | 0.000452 | *XYLT1\|CHSTB* |
| Apoptosis | gga04210 | 0.00128 | *IRAK2\|CASP8\|CFLAR* |
| Ubiquitin mediated proteolysis | gga04120 | 0.001292 | *RHBT1\|VHL\|SMUF2\|FBXW8* |
| Selenocompound metabolism | gga00450 | 0.003289 | *TRXR1* |
| Glycosaminoglycan biosynthesis - heparan sulfate/heparin | gga00534 | 0.009037 | *XYLT1* |
| SNARE interactions in vesicular transport | gga04130 | 0.014455 | *STX2\|USE1* |
| Tight junction | gga04530 | 0.01495 | *RAB3C* |
| Non-homologous end-joining | gga03450 | 0.020674 | *RAD50* |
| Progesterone-mediated oocyte maturation | gga04914 | 0.036911 | *CDK1\|MD1L1\|ADCY8* |
| Gap junction | gga04540 | 0.04642 | *CDK1\|ADCY8\|GCYA1* |

Table S17 KEGG pathways significantly enriched by putatively selected genes of body size

| Term | ID | Input number | P-Value | Input |
| --- | --- | --- | --- | --- |
| Adherens junction | gga04520 | 6 | 1.51E-06 | *PARD3\|TGFR2\|CTNA3* |
| Tight junction | gga04530 | 4 | 0.003002 | *CTNA3\|PARD3* |
| Ubiquitin mediated proteolysis | gga04120 | 4 | 0.003568 | *UBE4B\|BIRC6* |
| Neuroactive ligand-receptor interaction | gga04080 | 5 | 0.009688 | *PARD3\|P2Y13\|P2Y14\|CALCR* |
| Endocytosis | gga04144 | 4 | 0.015634 | *PARD3\|TGFR2\|* |
| Wnt signaling pathway | gga04310 | 3 | 0.022161 | *NFAC1\|FZD1\|CNBP1* |
| Protein processing in endoplasmic reticulum | gga04141 | 3 | 0.036996 | *STT3B\|UBE4B* |
| TGF-beta signaling pathway | gga04350 | 2 | 0.047869 | *TGFR2* |

Table S18 Information for chicken breeds used in the current study

| Breed name | Acronym | Sampling location | Sample size | Attitude(m) | Annual average temperature(℃) |
| --- | --- | --- | --- | --- | --- |
| Changshun green egg chickens | CS | Southeast of Guizhou province of China | 15 male and 15 female | 1200 | 16.3 |
| Qiandongnan xiaoxiang chicken | QD | Southeast of Guizhou province of China | 15 male and 15 female | 613 | 16.5 |
| Puding gaojiao chickens | PD | Central region of Guizhou province of China | 15 male and 15 female | 1395 | 15.5 |
| Xingyi aijiao chickens | XY | Southwest of Guizhou province of China | 15 male and 15 female | 1250 | 16 |
| Chishui black-bone chickens | CSWG | Northeast of Guizhou province of China | 15 male and 15 female | 1000 | 18.1 |
| Wumeng black-bone chickens | WM | Northwest of Guizhou province of China | 15 male and 16 female | 1511 | 14.8 |
| Weining chickens | WN | Northwest of Guizhou province of China | 15 male and 15 female | 2400 | 9 |
| Yaoshan chickens | YS | Southeast of Guizhou province of China | 15 male and 14 female | 400 | 18.3 |
| Tianfu broilers | TF | Near northwest of Guizhou province of China | 15 male and 15 female | 750 | 16.1 |
| Shanghai xinyang layers | SHXY | Near northwest of Guizhou province of China | 15 male and 15 female | 750 | 16.1 |

*The extreme maximum temperature of the area CSWG located > 40 ℃ while the WM and WN were located in alpine regions with the extreme minimum temperature < -8 ℃.

Table S19 The general phenotypic characteristic of RJF, local Guizhou chicken breeds, commercial layer and broiler chicken breeds

| Items | RJF | CS | QD | PD | XY | CSWG | WM | WN | YS | TF | SHXY |
| --- | --- | --- | --- | --- | --- | --- | --- | --- | --- | --- | --- |
| Skin color | White | White | White | White | White | black | black | White | White | White | White |
| Age at 50% egg production rate (day) | 252 | 165 | 180 | 280 | 150 | 180 | 167 | 194 | 142 | — | 143 |
| Egg weight at 300 days of age (g) | 29.15 | 51.8 | 46.3 | 55.8 | 49.2 | 61.7 | 53.2 | 51.9 | 46.5 | — | 63.3 |
| Adult weight (♂) (g) | — | 1720.43 | 1614.38 | 2596.29 | 1912.53 | 2490.92 | 2853.98 | 3026 | 2162.31 | 4591.33 | — |
| Adult weight (♀) (g) | — | 1348.24 | 1415.87 | 2037.36 | 1529.56 | 1948.95 | 2193.21 | 2407.27 | 2035.39 | 3308.23 | — |
| Slaughter rate (♂) (%) | — | 86.56 | 89.21 | 87.45 | 84.99 | 90.09 | 91.53 | 89.27 | 87.58 | 88.05 | — |
| Slaughter rate (♀) (%) | — | 86.09 | 91.2 | 90.4 | 88.66 | 90.51 | 92.28 | 92.38 | 89.96 | 86.73 | — |
| Whole net carcass rate (♂) (%) | — | 71.49 | 76.39 | 75.28 | 73.85 | 79.68 | 76.90 | 75.33 | 75.93 | 78.45 | — |
| Whole net carcass rate (♀) (%) | — | 66.92 | 69.52 | 67.81 | 61.57 | 69.42 | 68.51 | 64.47 | 67.95 | 67.53 | — |
| Half net carcass rate (♂) (%) | — | 75.76 | 80.21 | 79.30 | 78.52 | 83.20 | 84.13 | 81.77 | 79.28 | 84.11 | — |
| Half net carcass rate (♀) (%) | — | 72.68 | 77.63 | 75.11 | 72.07 | 76.37 | 78.45 | 75.85 | 76.24 | 81.72 | — |
| Leg muscle rate (♂) (%) | — | 23.71 | 24.11 | 24.84 | 24.24 | 26.54 | 22.58 | 24.75 | 23.95 | 24.61 | — |
| Leg muscle rate (♀) (%) | — | 21.47 | 18.70 | 20.19 | 19.27 | 20.38 | 17.61 | 19.25 | 18.69 | 20.04 | — |
| Breast muscle rate (♂) (%) | — | 13.49 | 14.67 | 14.39 | 13.33 | 12.93 | 14.15 | 14.88 | 13.55 | 17.91 | — |
| Breast muscle rate (♀) (%) | — | 16.55 | 16.78 | 16.3 | 15.89 | 14.1 | 15.35 | 16.57 | 14.48 | 19.46 | — |
| Body slanting length (♂) (cm) | — | 20.89 | 19.75 | 23.41 | 21.15 | 22.33 | 22.99 | 22.77 | 22.1 | 29.7 | — |
| Body slanting length (♀) (cm) | — | 29.25 | 17.6 | 20.81 | 18.48 | 19.73 | 19.88 | 20.05 | 19.87 | 25.4 | — |
| Chest breadth (♂) (cm) | — | 7.2 | 7.3 | 9.1 | 8.3 | 8.8 | 9.4 | 9.2 | 8.4 | 10.2 | — |
| Chest breadth (♀) (cm) | — | 6.3 | 6.8 | 8.1 | 7.1 | 7.9 | 7.9 | 8.1 | 7.5 | 9.1 | — |
| Chest depth (♂) (cm) | — | 9.0 | 8.7 | 10.1 | 9.6 | 9.2 | 9.7 | 10.2 | 9.2 | 11.0 | — |
| Chest depth (♀) (cm) | — | 8.1 | 7.8 | 8.8 | 8.4 | 8.1 | 8.3 | 8.9 | 8.2 | 9.9 | — |
| Shank length (♂) (cm) | — | 10.1 | 9.4 | 12.5 | 8.5 | 11.2 | 10.9 | 11.2 | 10.8 | 13.6 | — |
| Shank length (♀) (cm) | — | 8.8 | 8.0 | 10.3 | 7.1 | 9.5 | 9.3 | 9.5 | 9.4 | 10.5 | — |
| Shank circumference (♂) (cm) | — | 4.5 | 4.1 | 5.2 | 5.3 | 5.2 | 5.3 | 5.3 | 4.8 | 6.1 | — |
| Shank circumference (♀) (cm) | — | 4 | 3.5 | 4.2 | 4.3 | 4.3 | 4.4 | 4.2 | 4.1 | 4.7 | — |

Note: adult weight, slaughter rate, whole net carcass rate, half net carcass rate, leg muscle rate, breast muscle rate, body slanting length, chest breadth, chest depth, shank length and shank circumference traits all refer to adults. The phenotypic data of RJF were collected from references while those of Guizhou breeds and commercial breeds were evaluated by ourself. “—” indicates unavailable data.

Table S20 residuals and variance of relatedness between populations for TreeMix analysis

| m value | Residuals | Variance of relatedness between populations explained by the model |
| --- | --- | --- |
| 1 | 42.4 | 0.927245195 |
| 2 | 43.1 | 0.944596351 |
| 3 | 23.9 | 0.969395745 |
| 4 | 15.7 | 0.980063813 |
| 5 | 14.2 | 0.985340776 |
| 6 | 9.9 | 0.989074355 |
| 7 | 10.1 | 0.990451848 |
| 8 | 9.3 | 0.991399697 |
| 9 | 7.2 | 0.995726994 |
| 10 | 9.1 | 0.995703854 |

Table S21 Cluster information for Genome-wide selective sweep analysis

| Clusters | Target character | Reference population | Objective population |
| --- | --- | --- | --- |
| Clusters 1 | Domestication | RJF | CS |
| Cluster 2 | Domestication | RJF | CSWG |
| Clusters 3 | Domestication | RJF | PD |
| Cluster 4 | Domestication | RJF | QD |
| Clusters 5 | Domestication | RJF | WM |
| Cluster 6 | Domestication | RJF | WN |
| Clusters 7 | Domestication | RJF | XY |
| Cluster 8 | Domestication | RJF | YS |
| Clusters 9 | Layer feature | CS, CSWG, PD, QD, WM, WN, XY, YS | SHXY |
| Cluster 10 | Broiler feature | CS, CSWG, QD, WM, WN, XY, YS | TF |
| Clusters 11 | Body size | QD, XY | PD |
