# Supplementary material for: Whole-genome sequencing revealed genetic diversity, structure and patterns of selection in Guizhou indigenous chickens
Source: BMC Genomics. 2023 Sep 26;24:570. doi: 10.1186/s12864-023-09621-w (PMC10521574; doi:10.1186/s12864-023-09621-w)
Supplement: Supplementary file 2 — Additional file 2. This file includes Figures S1 to S7. [file 12864_2023_9621_MOESM2_ESM.docx]

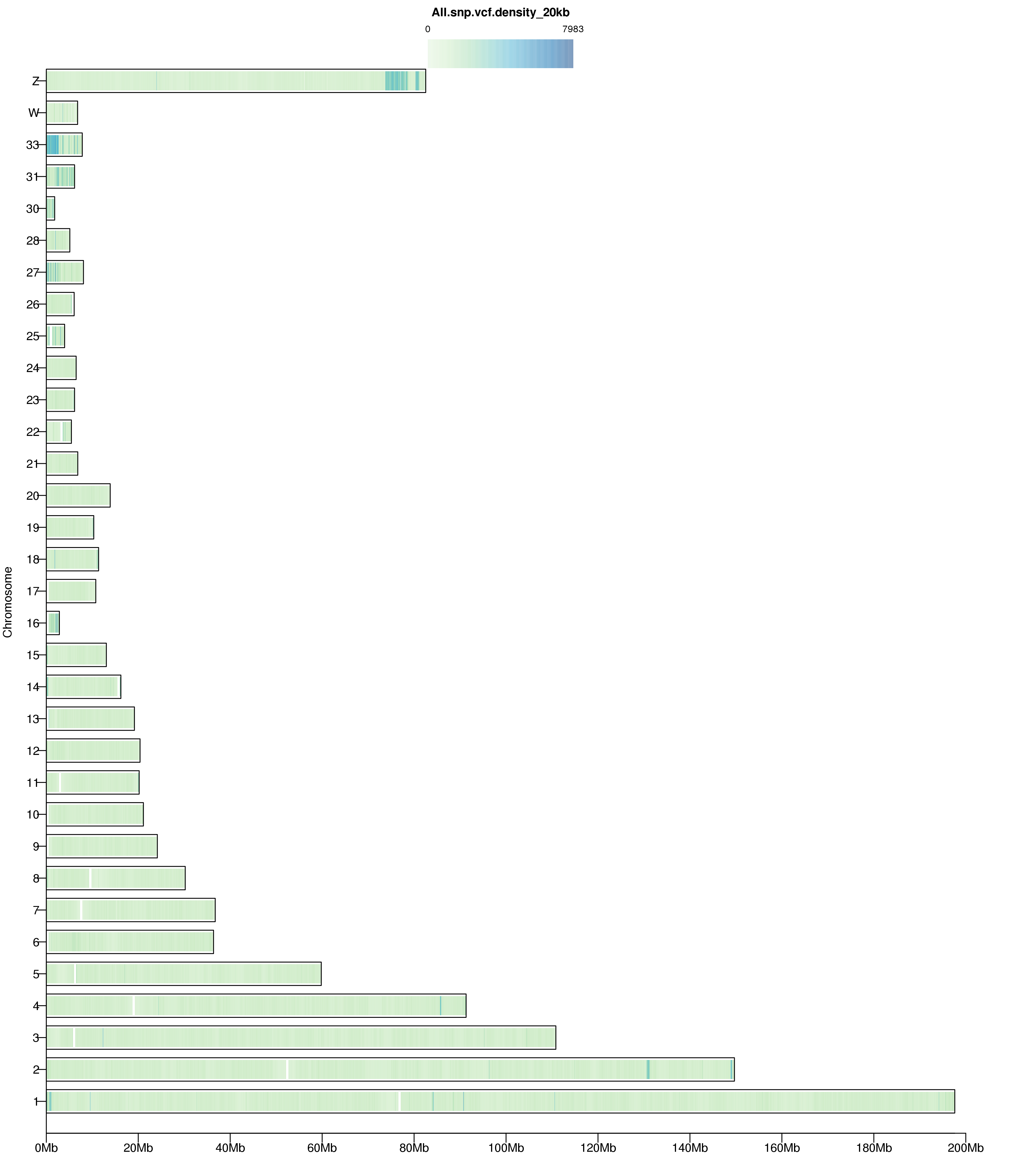


Figure S1 Distribution map of SNPs on chromosome


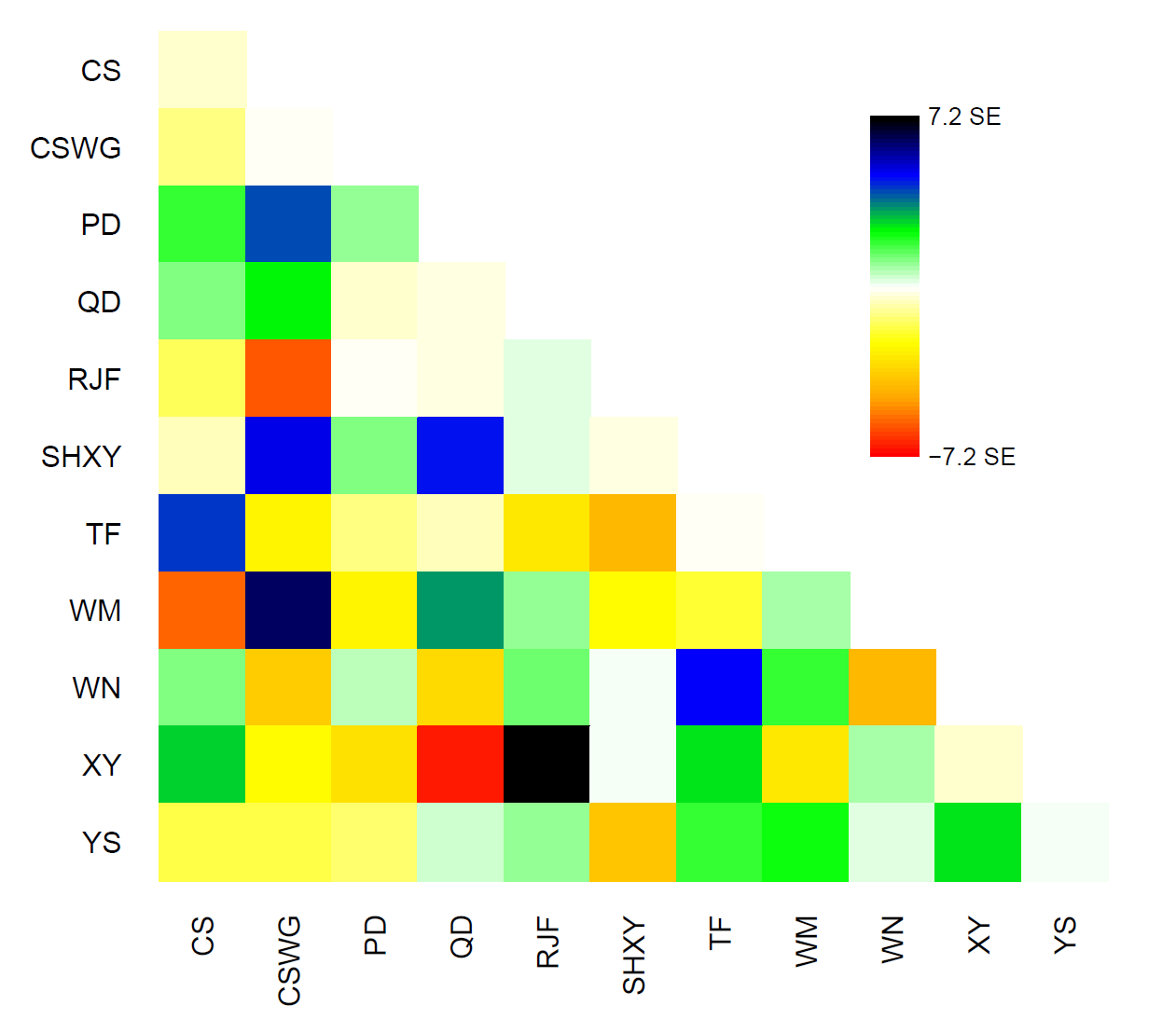


Figure S2 The residual matrix of 9 migration event scenarios


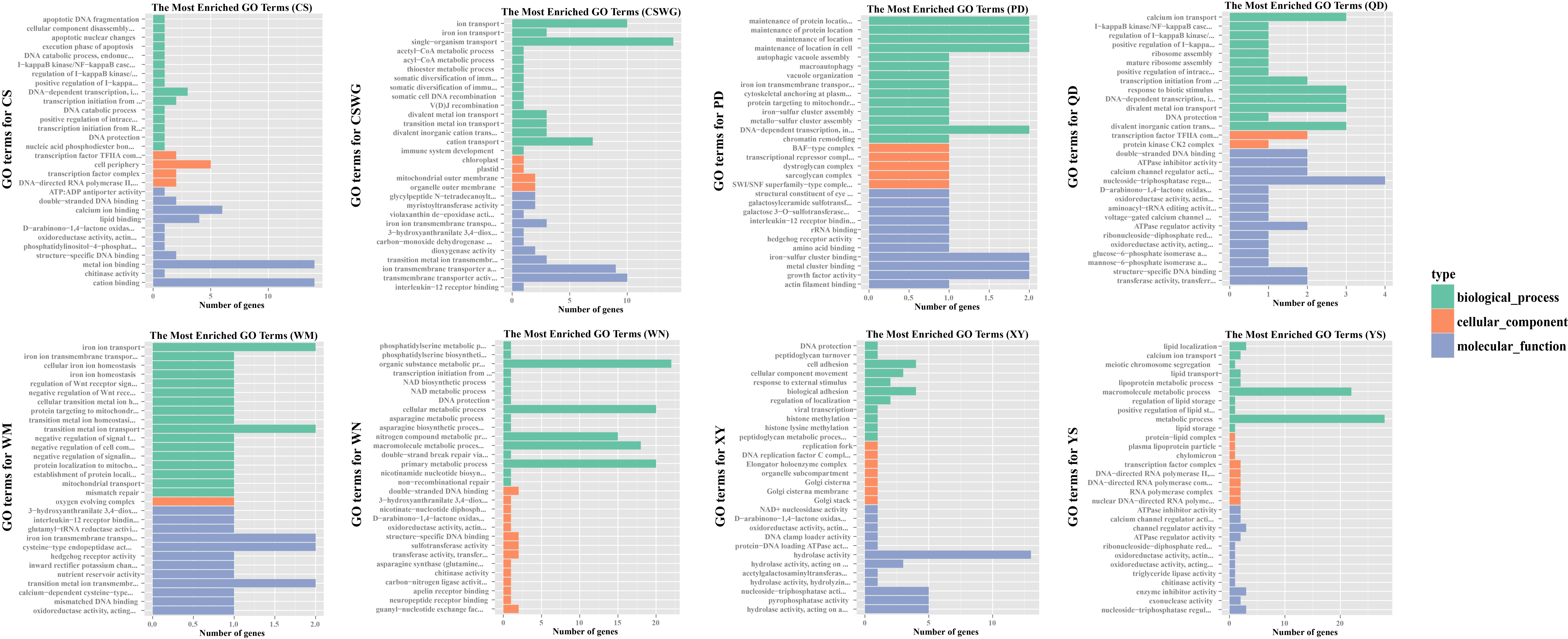


Figure S3 Go terms analysis for each Guizhou indigenous chicken breeds (Terms of top 30)


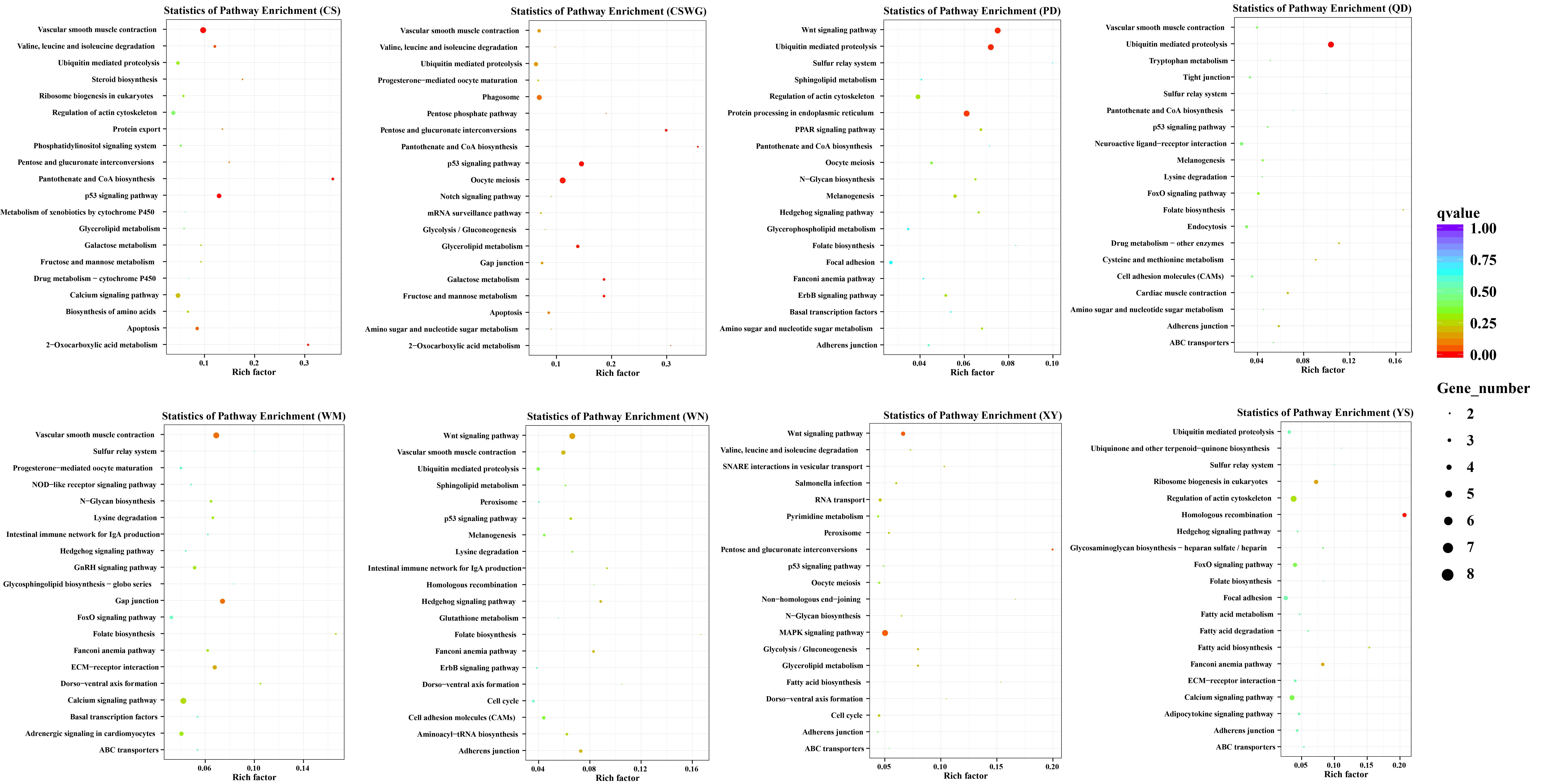


Figure S4 KEGG pathways analysis for each Guizhou indigenous chicken breeds (Terms of top 20) We have got permission to use the KEGG software from the Kanehisa laboratory (http://www.kegg.jp/kegg/kegg1.html).


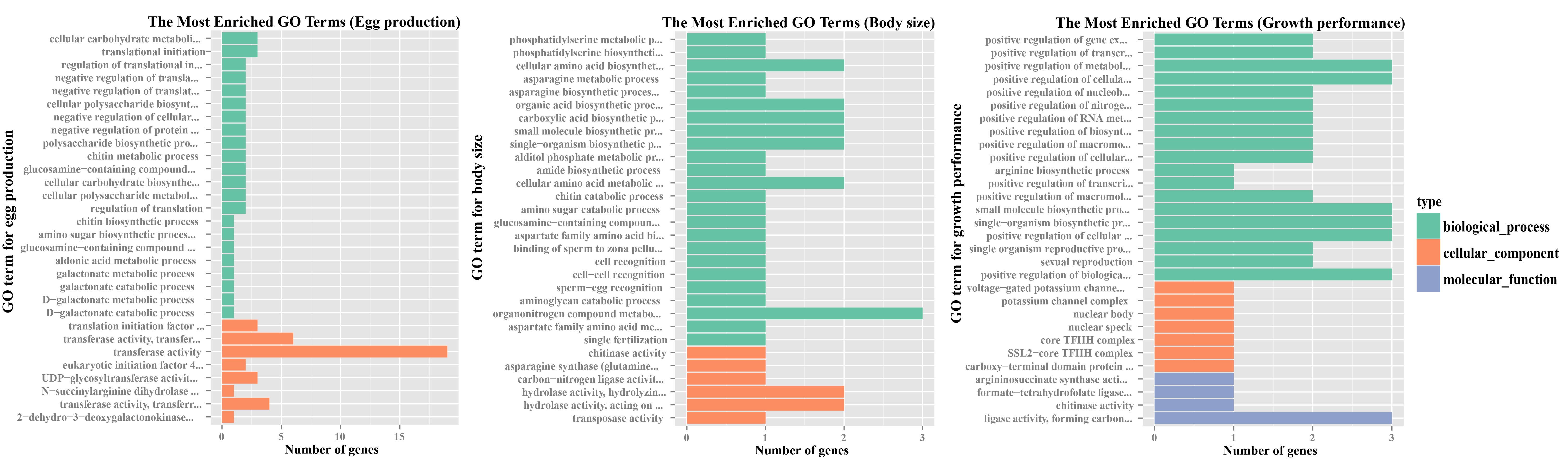


Figure S5 Go terms analysis for different traits (Terms of top 30)


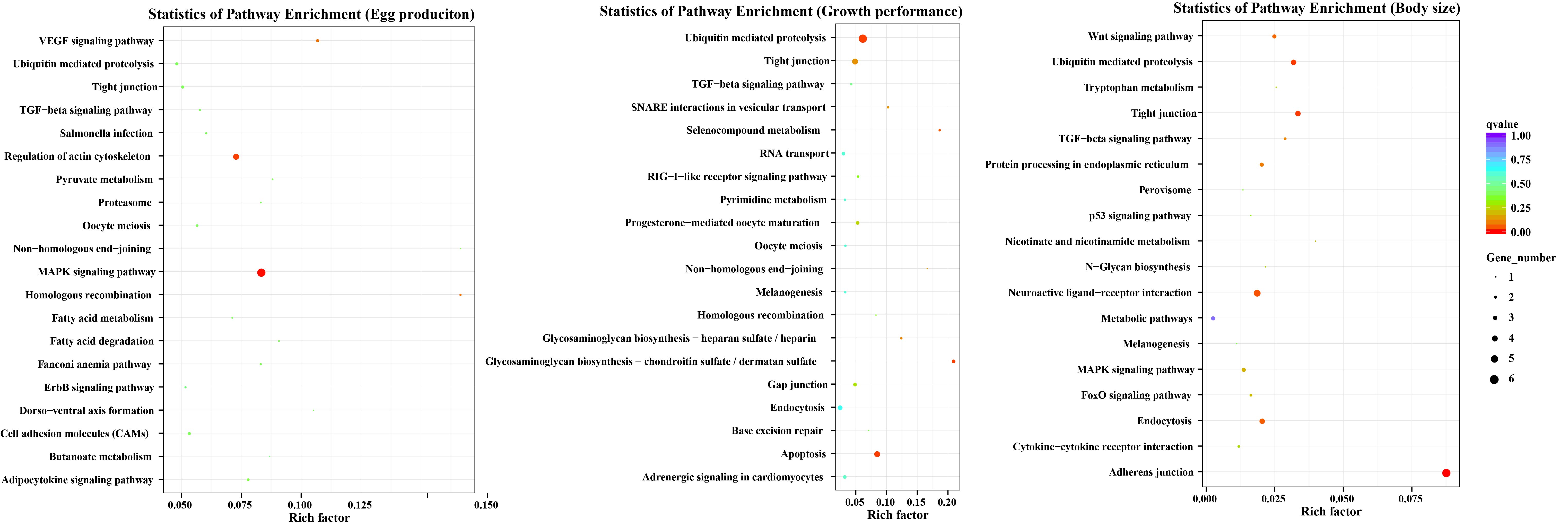


Figure S6 KEGG pathways analysis for different traits (Terms of top 20). We have got permission to use the KEGG software from the Kanehisa laboratory (http://www.kegg.jp/kegg/kegg1.html).


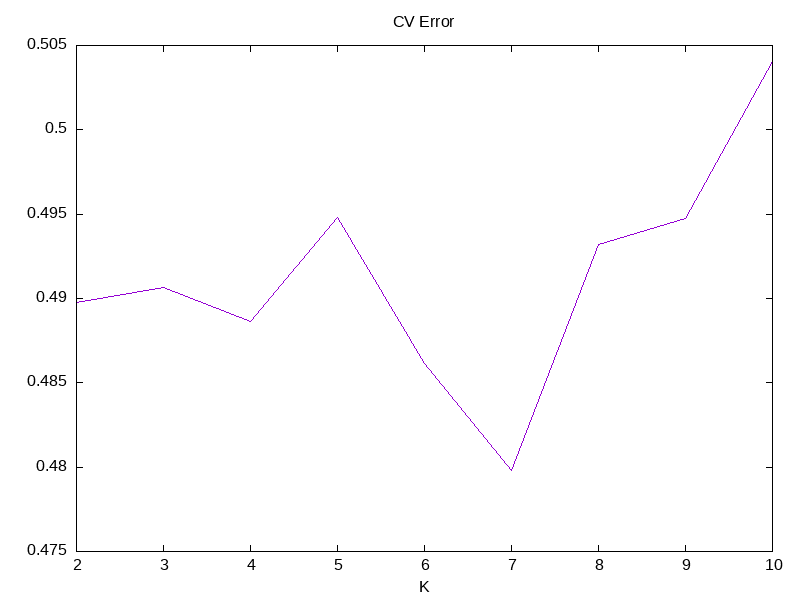


Figure S7 Cross validation error rate for ADMIXTURE study
